# Superconducting coherence boosted by outer-layer metallic screening in multilayered cuprates

**DOI:** 10.1038/s41467-026-75295-z

**Published:** 2026-07-14

**Authors:** Junhyeok Jeong, Kifu Kurokawa, Shiro Sakai, Tomotaka Nakayama, Kotaro Ando, Naoshi Ogane, Soonsang Huh, Matthew D. Watson, Timur K. Kim, Cephise Cacho, Chun Lin, Makoto Hashimoto, Donghui Lu, Takami Tohyama, Kazuyasu Tokiwa, Takeshi Kondo

**Affiliations:** 1 Institute for Solid State Physics, the University of Tokyo, Kashiwa, Japan; 2 Faculty of Science and Technology, Sophia University, Tokyo, Japan; 3 Department of Applied Electronics, Tokyo University of Science, Tokyo, Japan; 4 Diamond Light Source Ltd, Harwell Science and Innovation Campus, Didcot, UK; 5 Stanford Synchrotron Radiation Lightsource, SLAC National Accelerator Laboratory, Menlo Park, CA USA; 6 Department of Applied Physics, Tokyo University of Science, Tokyo, Japan; 7 Trans-scale Quantum Science Institute, The University of Tokyo, Bunkyo-ku, Japan

**Keywords:** Superconducting properties and materials, Electronic properties and materials

## Abstract

In multilayered high-*T*_c_ cuprates, the inner CuO_2_ planes (IPs) are spatially separated from dopant layers and thus remain cleaner than the outer planes (OPs). Here, we investigate five-layer (Cu,C)Ba_2_Ca_4_Cu_5_O_*y*_ (Cu1245) and three-layer Ba_2_Ca_2_Cu_3_O_6_(F,O)_2_ (F0223) using angle-resolved photoemission spectroscopy (ARPES) and uncover an unprecedented situation, in which only the IPs become superconducting while the OPs remain metallic at low temperatures. Our model calculations indicate that over 95% of the OP wavefunction remains confined to OP itself, rendering superconducting proximity negligible. This interlayer decoupling realizes an ideal configuration—a single superconducting CuO_2_ layer screened from the dopant layer-induced disorder by heavily overdoped metallic OPs. The clean CuO_2_ layer exhibits a large superconducting gap with coherent Bogoliubov peaks extending beyond the antiferromagnetic zone boundary, and a widely extended coherent flat band at the Brillouin zone edge. These findings introduce the “degree of screening” as a physical parameter for probing competition between pseudogap and superconductivity, and for bridging typically disordered real materials with the idealized theoretical models.

## Abstract (web summary)

The authors experimentally investigate the electronic structure of (Cu,C)Ba_2_Ca_4_Cu_5_O_y_ and Ba_2_Ca_2_Cu_3_O_6_(F,O)_2_, with five and three CuO_2_ layers in the unit cell, respectively. They find that only the inner planes become superconducting while the outer planes remain metallic and screen the inner planes from the disorder of the dopant layers.

## Introduction

Understanding the microscopic origin of high-temperature superconductivity in cuprates is arguably one of the most central unsolved problems in condensed matter physics. To date, most investigations have concentrated on single- and double-layered cuprates, due to their structural simplicity and relative ease of crystal growth. However, scanning tunneling microscopy (STM)^1,2^ has revealed that CuO_2_ layers in these systems are inevitably disordered due to their close proximity to dopant layers, which introduce random potentials. Such disorder effects hinder direct quantitative comparison with microscopic theories, which generally assume ideal, disorder-free CuO_2_ sheets. Resolving this theory-reality mismatch may bring a breakthrough in formulating a microscopic theory of pairing in high-*T*_c_ cuprate research.

This fundamental problem can be overcome by the multilayered cuprates, in which three or more CuO_2_ planes stack within a single unit cell. In general notation, the CuO_2_ planes in the multilayered system are classified into inner planes (IPs) and outer planes (OPs), with their doping levels (or local potentials) varying with the relative distance to dopant layers. The OPs directly contact the dopant layers, whereas the IPs do not and are protected by adjacent OPs. The elemental-site sensitive nuclear magnetic resonance (NMR) measurement demonstrates that the IPs are flat and clean, maintaining a homogeneous electronic state, which is unattainable in single- and double-layer compounds^3,4^. Above all, we should keep in mind that three-layer cuprates, which achieve the highest superconducting (SC) transition temperatures (*T*_c_) among existing materials^5^, also host clean IPs; thereby, unveiling the electronic properties of IPs and their relationship with OPs holds the key for elucidating the pairing mechanism in the high-*T*_c_ cuprates.

Recently, five- and six-layer cuprates with apical-fluorine structures have been found to exhibit small Fermi pockets in clean inner CuO_2_ layers, which is a long-anticipated electronic structure that had eluded experimental realization^6,7^. Interestingly, while superconductivity occurs in the second inner and outer layers, the innermost layer is reported to remain metallic at the lowest temperatures. However, the SC gap observed in these systems was considerably smaller than that in other cuprates^8–15^, leaving open the possibility that the innermost layer hosts proximity-induced superconductivity, which is too weak to detect. In addition, their underdoped outer layer hosts superconductivity at low carrier density, making it highly susceptible to disorder from the directly adjacent dopant layers. Such a disordered OP environment may hinder the penetration of superconducting proximity effects into the inner layers.

A better understanding of multilayer cuprates may be gained by exploring the inverse configuration: the inner, cleaner layer is superconducting, but the outer layers remain metallic at the lowest temperatures. It is particularly intriguing to consider a scenario in which the IPs open a large SC gap, and the OPs are metallic with highly dense carriers. This naturally realized rare heterostructure offers a unique opportunity to distinguish intertwined effects across multiple layers on enhancing *T*_c_ in cuprates. Most importantly, this rather unique yet simple material setting, in which CuO_2_ layers are shielded by metallic layers with dense mobile carriers, may help bridge the gap between real materials and idealized theoretical models, where disorder effects are usually neglected. Moreover, this specific material system may provide insights into artificial heterostructures combining high-*T*_c_ cuprates and other compounds, such as topological insulators, with the ultimate goal of realizing topological superconductivity^16–19^. This study, therefore, not only allows for the investigation of the properties of multilayer cuprates but also sheds light on interlayer physics^20^.

In this article, we report an unprecedented configuration in cuprates, where superconducting CuO_2_ planes are sandwiched between highly conductive metallic sheets. These metallic sheets effectively screen out disorder from the dopant layers, allowing us to introduce a physical parameter—the “degree of screening”—to access the intrinsic electronic properties of cuprates. In particular, we demonstrate that the inner CuO_2_ planes in a multilayer high-*T*_*c*_ cuprate host the large SC gap, while the heavily overdoped metallic outer layers show no superconducting signals or proximity effect. Our model calculations reveal that the large energy (or doping) difference between layers blocks interlayer coupling, preventing a superconducting proximity between the inner and outer planes. Meanwhile, the highly conductive metallic outer layers shield the inner planes from disorder, significantly enhancing superconducting coherence and superfluid density. Consequently, even a single CuO_2_ layer can exhibit both a large pairing gap and strong phase stiffness when disorder is removed. We detect a coherent flat band near the zone edge, shedding light on the origin of the pseudogap—whether it stems from a charge density wave (CDW), a pair density wave (PDW), or other mechanisms. These findings offer a promising route to address the mismatch between real materials and idealized theoretical models that often neglect disorder effects.

## Results

**Fig. 1:**
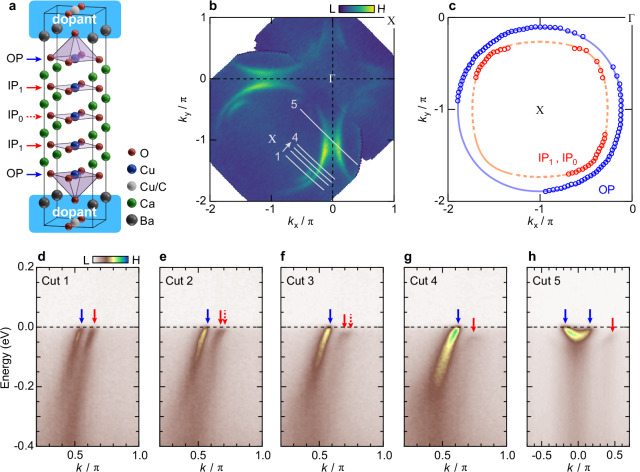
Fermi surface and band dispersions of the five-layer Cu1245. **a** Crystal structure of Cu1245. **b** Fermi surface (FS) of Cu1245 measured by ARPES. Gray lines indicate the momentum cuts used to show the dispersions in (**d**–**h**). **c** Fermi surface determined by the FS map in (**b**). Red and blue circles indicate the Fermi momentum (*k*_F_) points of IP and OP, respectively. Colored lines are the tight-binding fit to the data. **d**–**h** ARPES band dispersions along momentum cuts indicated in (**b**). Blue and red arrows mark the location of *k*_F_ for the OP and IP bands. In Cuts 2 and 3, two bands from IPs are observed.

We begin by presenting the overall electronic structure of Cu1245 with *T*_c_ = 78 K. In multilayered cuprates, carriers in the CuO_2_ planes are distributed from the dopant layers. The doping level, therefore, decreases as the spatial distance from the dopant layers increases^3,4,21,22^. The five-layered Cu1245 (Fig. 1a) consists of three doping-inequivalent CuO_2_ planes: outer planes (OP), and two inner planes (IP_1_ and IP_0_). We observe essentially two Fermi surfaces (FSs), both hole-like and centered at (*π*, *π*) (Fig. 1b). According to the above discussion, the larger FS is primarily from the OPs, while the smaller one originates mainly from the IPs. Although a slight band splitting between IP_0_ and IP_1_ is expected, it is too small to resolve. This may arise not only from limited equipment resolution but also from unavoidable surface roughness due to the lack of a natural cleavage plane in Cu1245. This causes spectral broadening, hindering the identification of the band separation for IP_0_ and IP_1_. Thereby, we treat IP_1_ and IP_0_ together as a single “IP" band. The Fermi momenta *k*_F_ extracted from the FS mapping are plotted in Fig. 1c, with the red and blue circles for IP and OP, respectively. From the FS volume of OP, we estimate a hole-carrier concentration of *p*(OP) ~ 28%. In contrast, the IP band forms a truncated Fermi arc, within which coherent peaks are particularly sharp and clear.

Figure 1d–h display the ARPES band dispersions from the nodal to the antinodal region. The IP bands marked by red arrows show a band structure typical of cuprates with a *d*-wave SC gap. While the nodal dispersion crosses the *E*_F_ with no gap, an SC gap opens off the node and increases toward the antinode. Near the antinodal region, the Bogoliubov back-bending becomes unclear since coherent peaks are strongly suppressed due to the development of the pseudogap^23,24^. This makes it difficult to determine the *k*_F_ from the gap minimum (dashed line in Fig. 1c).

**Fig. 2:**
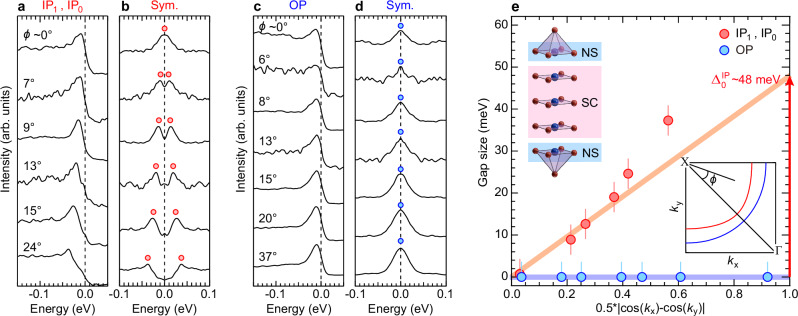
Superconducting gaps in the five-layer Cu1245. **a** EDCs of IP taken at various *k*_F_ points. *ϕ* is the angle from the node, as indicated in the right-bottom inset of (**e**). **b** Symmetrized EDCs of (**a**). Colored circles indicate SC coherent peak positions. **c**,**d** Similar measurement as (**a**,**b**), respectively, but for the OP band. **e** Summary of the SC gap sizes of Cu1245, plotted as a function of *d*-wave form. The top-left inset summarizes our results for the Cu1245 sample: IP is under SC state, while OP is under normal state (NS).

Strikingly, the OP band (blue arrows) shows very different behavior from the IPs. The band dispersion crosses the *E*_F_ along the entire FS. The contrasting results of OPs are more clearly demonstrated in Fig. 2c, d, where the raw EDCs at *k*_F_ and those symmetrized across *E*_F_ are plotted for the different Fermi angle *ϕ* (right-bottom inset in Fig. 2e). Interestingly, OP is gapless all along the FS. This is in stark contrast to a *d*-wave-like gap behavior typical for underdoped cuprates, observed in IP (Fig. 2a, b).

Figure 2e summarizes the gap magnitude for both layers plotted against the *d*-wave form, ${\Delta }_{0}| \cos ({k}_{{{{\rm{x}}}}})-\cos ({k}_{{{{\rm{y}}}}})| /2$, where Δ_0_ is the extrapolated gap to the antinode. IP features a clear *d*-wave superconducting property, having a large ${\Delta }_{0}^{{{{\rm{IP}}}}} \sim 48\,{{{\rm{meV}}}}$. A deviation from the monotonic *d*-wave structure near the antinode is a hallmark of the two-gap states due to the presence of the pseudogap typical in underdoped cuprates^10,23–26^. On the other hand, OP shows no gap, which extends to the antinodal region. Given that the OP layer is heavily overdoped (*p*(OP) ~ 28%), and nearly constant peak width at the Fermi energy (*E*_F_) along wide proportions of FS (Fig. 2c, d), this no-gap feature indicates that OPs are in a normal metallic state, without a notable proximity effect induced from IPs. We note here that the current situation differs from Bi_2_Sr_2_Ca_2_Cu_3_O_10+*δ*_ (Bi2223), which exhibits significant interlayer mixing due to Bogoliubov band hybridization^14,27,28^: The superconducting OP band causes Bogoliubov back-bending. This results in a band crossing with the IP band, inducing significant interlayer mixing. This is not the case in the current compound, since the OP band is metallic at low temperatures.

**Fig. 3:**
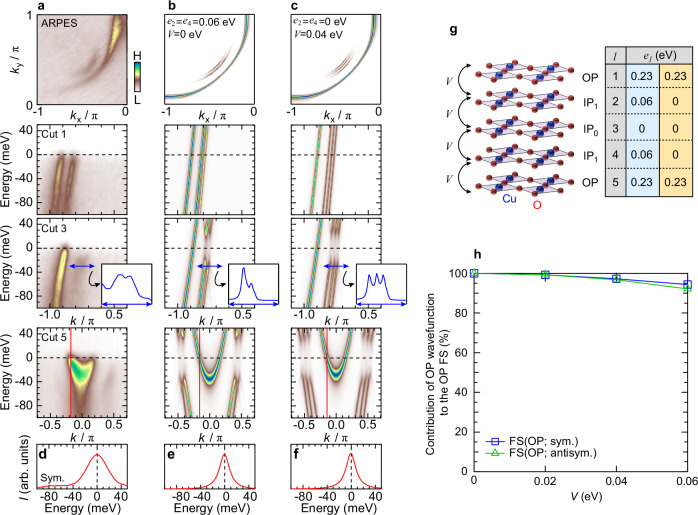
Model calculations for the five-layer Cu1245. **a** Enlarged plots of ARPES data of Cu1245 shown in Fig. 1. The first row corresponds to FS mapping. The second, third, and fourth rows correspond to Cut 1, 3, and 5 of Figs. 1d, f, h, respectively. The inset of Cut 3 shows an MDC curve along the blue arrow. **b** Model calculations based on (**a**), by setting IP_1_ potential *e*_2_ = *e*_4_ = 0.06 eV, and zero interlayer hopping *V* = 0 eV. **c** Similar model calculations with (**b**), but setting zero IP_1_ potential *e*_2_ = *e*_4_ = 0 eV, and interlayer hopping *V* = 0.04 eV. For Cut 3 of (**a**–**c**), MDC along a blue arrow is extracted in the inset. **d** Symmetrized EDC taken at *k*_F_ from the OP band in Cut 5. This is the same curve as Fig. 2d (37^∘^). **e**,**f** Similar to (**d**), but for the model calculations of (**b**,**c**). **g** Schematic illustration for the model calculation. *V* and *e*_*l*_ are the interlayer hopping and layer-dependent electronic potential, respectively. Right table shows *e*_*l*_ values set to obtain (**b**,**c**). **h** The weight of the OP wavefunction contribution to the FS of OP itself. Since there are two equivalent OPs in the five-layer system, we obtain two different low-energy states for the FS. (sym. and antisym.). *e*_*l*_ are set to the same value as those in (**c**).

To quantify the absence of the interlayer SC proximity effect, we perform a tight-binding model calculation on the five-layer structure. We find that two factors are important for the proximity effect: the layer-dependent potential *e*_*l*_ (depending on the distance from the dopant layers) and the interlayer hopping *V* (Fig. 3g). Although both parameters lead to band separation in energy, their physical roles differ distinctly: *V* yields interlayer wavefunction mixing, while *e*_*l*_ difference between layers *δe* suppresses it. A variety of *V*, *e*_*l*_ combinations can reproduce the ARPES data. However, we can estimate the upper bound of *V* from the data and demonstrate that such a *V* value is not large enough to open a measurable energy gap in the OP band by the proximity effect from the superconducting IPs (see the Supplementary NOTE 2 for more details).

To address this, we display in Fig. 3a the ARPES data of the FS and the band dispersions for Cuts 1, 3, and 5 shown in Fig. 1. Calculated results are shown alongside each panel. In both cases in Fig. 3b, c, the SC gap is set to ${\Delta }_{0}^{{{{\rm{IP}}}}} \sim 48\,{{\rm{meV}}}$ for IP and zero for OP. Now, we simulate the bands using two specific combinations for parameters *V* and *e*_*l*_. In Fig. 3b, we set *V* = 0 and choose appropriate *e*_*l*_ for each layer to reproduce the data, as listed in the table of Fig. 3g. The *e*_*l*_ for IP_0_ and IP_1_ can be particularly guided by the data of Cut 3, which clearly shows a splitting, as demonstrated by extracting the momentum distribution curve (MDC) along the blue arrow in the inset. In this case, the wavefunction mixing is zero simply because the interlayer hopping is set to zero. As expected, the OP spectra show no indication of gap opening (Fig. 3e).

The upper bound of *V* yielding the interlayer mixing can be found as follows. For this purpose, we focus on the splitting of the IP_1_ and IP_0_ bands, which is relatively small. Now, we set the potential difference *δe* between IP_1_ and IP_0_ to zero, and reproduce their spectral splitting by choosing a value of *V* (insets of Cut 3 in Fig. 3). We find that a *V* value, which reproduces the data, lies between 0.02 and 0.04 eV. Using the larger *V* (0.04 eV), the calculated band splitting of IPs exceeds the experimental one, as demonstrated in the inset of Fig. 3c, which shows an MDC along the blue arrow. Notably, the calculations show triple bands splitting, which is not observed in the ARPES data. Most importantly, even the overestimated *V* (0.04 eV) does not open a gap in the OP band (Fig. 3f). This indicates that the SC proximity effect from IPs to the OP band is negligible in the real material.

In Fig. 3h, we estimate the percentage of the wavefunction confinement within OP with respect to *V* values. We note that the OP has two low-energy eigenstates, since there are two equivalent sheets of the outer layer in the crystal structure (Fig. 1a). As a result, we get two different low-energy states for the *k*_F_ points of OP: symmetric (sym.) and antisymmetric (antisym.). Figure 3h displays both results. Even for the upper bound *V* (0.04 eV), both results show that more than 95% of the spectral weight for the OP band is derived from the OP itself without significant wavefunction mixing from IPs. We note this OP-isolation is mainly due to a large *e*_*l*_ difference between OP and IPs. This suggests that a large energy (or doping) difference between layers suppresses wavefunction mixing, rendering the SC proximity effect negligible in the five-layer Cu1245.

**Fig. 4:**
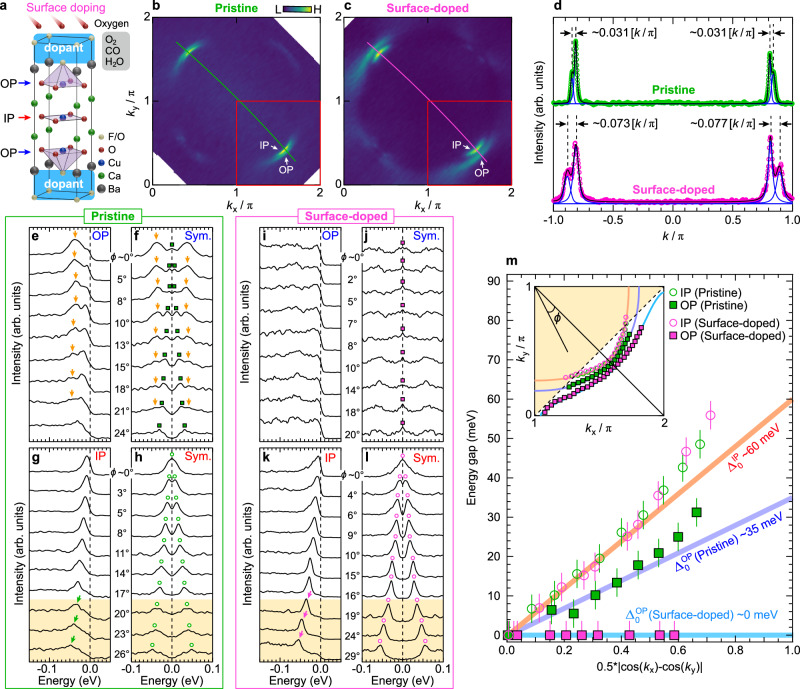
Fermi surface mapping and superconducting gap in the pristine and surface-doped three-layer F0223. **a** Crystal structure of F0223. We control the doping level of the sample by exposing it to a small air leak, which introduces oxygen to the sample surface and introduces hole carriers. **b**,**c** FS mappings for the pristine and the surface-doped sample, respectively. **d** MDC curves of the pristine and the surface-doped sample, taken along the colored line in (**b**,**c**), respectively. Black lines are fits obtained by linear backgrounds and the Voigt functions (blue lines). **e**,**f** Raw and symmetrized EDCs taken at various *k*_F_ points of the OP band in the pristine sample, respectively. The peaks indicated by orange arrows originate from the IP spectra. Colored squares indicate SC coherent peak positions. **g**,**h** Raw and symmetrized EDCs taken at various *k*_F_ points of the IP band in the pristine sample, respectively. Colored circles indicate SC coherent peak positions. **i**,**j** Similar to (**e**,**f**), but for the surface-doped sample. **k**,**l** Similar to (**g**,**h**), but for the surface-doped sample. Colored arrows in (**g**,**k**) mark EDCs at the *k*_F_ points beyond the antiferromagnetic zone boundary (AFZB) marked by a yellow shade in the inset of (**m**) toward the antinode. **m** Summary of the IP and OP gap magnitudes in the pristine and surface-doped sample, plotted as a function of *d*-wave form. Colored lines represent fits to the data near the node and are used to extrapolate the SC gap magnitude at the antinode (Δ_0_). The inset shows the determined Fermi surfaces and their tight-binding fit (colored lines). Errors of *k*_F_ are smaller than the symbol size.

The five-layer Cu1245 enabled us to conclude that interlayer hopping between the heavily overdoped OP and the underdoped IP can be sufficiently weak to render their interlayer mixing negligible. However, the presence of mutually coupled three IPs (two IP_1_s and one IP_0_) obscures the intrinsic properties of a clean, isolated CuO_2_ plane. Moreover, it has been reported that the IPs in Cu1245 are more sensitive to disorder effects than those in other multilayered systems^29^. To avoid these complexities, we now shift our focus to the three-layer F0223. This compound corresponds to *n* = 3 in apical-Fluorine cuprates ${{{{\rm{Ba}}}}}_{2}{{{{\rm{Ca}}}}}_{n-1}{{{{\rm{Cu}}}}}_{n}{{{{\rm{O}}}}}_{2n}{({{{{\rm{F}}}}}_{y}{{{{\rm{O}}}}}_{1-y})}_{2}$. Note that small Fermi pockets were observed in the lightly doped, clean IPs for the five-layer case (*n* = 5)^6^. The three-layer F0223 has the simplest multilayer structure with IPs, in which a single IP is sandwiched between two OPs in the unit cell (Fig. 4a). This compound is therefore ideally suited for the current study. Typically, three-layer cuprates have the highest *T*_c_ among series compositions^5^. A widely accepted view for explaining the particularly high *T*_c_ is that the underdoped IP hosts a large pairing gap but suffers from strong phase fluctuations, whereas the overdoped OP compensates for this by providing phase stiffness^30–32^. Although intriguing, and supported by experiment^14,27,28^, this scenario premises a hybridization between OP and IP^31,32^. An intriguing question is what happens if this interlayer hybridization is negligibly small, and the IP essentially remains isolated in the three-layer structure; this will be addressed below.

Figure 4b presents the FS mapping of pristine F0223 with *T*_c_ = 100 K, showing two spectral branches of IP and OP, which lie close to each other but are clearly separated. We find that both IP and OP open the SC gap (Fig. 4e–h, m), similar to those in optimally doped Bi2223: ${\Delta }_{0}^{{{{\rm{IP}}}}} \sim 60\,{{{\rm{meV}}}}$, and ${\Delta }_{0}^{{{{\rm{OP}}}}} \sim 35\,{{{\rm{meV}}}}$^14,33^. Yet, a clear difference is observed: Not only the IP band but also the OP band shows a two-gap behavior, deviating from the monotonic *d*-wave symmetry (Fig. 4m). The spectra with a larger energy scale exhibit a broad lineshape indicative of the pseudogap near the antinode (Fig. 4g, green arrows). This indicates that both IP and OP bands are located in the underdoped regime, being consistent with the lower *T*_c_=100 K of the current sample than *T*_c_ ~ 120 K for optimally doped F0223^3,34^.

For both IP and OP, the FSs show arc-like features: Coherent, sharp peaks are observed only in a limited region near the node, and broad spectra characteristic of a pseudogap are observed outside that arc region^23,24,35^. This makes it difficult to estimate doping levels accurately from the FS areas. The momentum splitting between IP and OP is resolvable, yet is notably smaller than that in optimally doped Bi2223^14,33^. This indicates that the doping-level difference between IP and OP in the present F0223 sample is smaller than that of optimally doped Bi2223. Arguments in Fig. 3 further suggest that the interlayer coupling likely exceeds that in optimally doped Bi2223.

To suppress interlayer coupling (or band hybridization) and reproduce the situation of an isolated clean IP, we perform surface doping on F0223 and increase the doping-level difference between the IP and OP. The widely used technique for surface doping employs alkali-metal deposition^7,36,37^, which, however, reduces hole doping in the underdoped regime. For hole-doping in cuprates, in-situ ozone annealing has been demonstrated to be effective^9,38^. In this study, instead, we used a rather simple yet robust method: exposing the cleaved surface to a small air leak ( ~ 3 × 10^−9^ Torr) in the measurement vacuum chamber. This method effectively increases the doping level toward the overdoped side, as previously demonstrated^39^.

In contrast to the pristine sample, the FS mapping of the surface-doped sample reveals a clearer separation between the IP and OP (Fig. 4c). In Fig. 4d, we extract momentum distribution curves (MDCs) along the nodal cuts indicated in Fig. 4b, c and fit them with Voigt functions to quantify the *k*_F_ splitting^40^. After surface doping, the separation between the IP and OP increases by more than a factor of two. Interestingly, however, the IP peak positions remain nearly unchanged, indicating that only the OP is significantly doped. This is further supported by full extraction of *k*_F_ points along the FS within the red box shown in Fig. 4b, c (inset of Fig. 4m). The OP band shifts significantly to the overdoped region. This lifts the saddle point at (*π*, 0) above *E*_F_, causing the Lifshitz transition from the original hole-like topology centered at (*π*, *π*) to an electron-like topology centered at (0, 0)^11,38,41^. Consequently, the doping level (*p*) estimated from the FS area is drastically increased from the underdoped regime (*p*(OP) ~ 8%) to the heavily overdoped regime (*p*(OP) ~ 30%), which typically lies outside the superconducting *T*_c_ dome^42^. In contrast, the IP band remains nearly unchanged between the pristine and surface-doped samples, indicating that any change in the IP doping level is small. The discrepancy in doping between OPs and IPs can be naturally explained by their multilayered structure. The surface doping primarily influences the dopant layer; consequently, the OPs adjacent to it are predominantly doped (see Supplementary Note 3 and Note 4 for more discussion).

We find that the layer-selective doping also alters the layer dependence of the SC gap. Figure 4i–l plot the raw and symmetrized EDCs for OP and IP at various *k*_F_ points of the surface-doped sample. Notably, the OP gap becomes entirely closed (Fig. 4j). This indicates that the OP becomes a heavily overdoped normal metallic state, similarly to the OPs of the five-layer Cu1245. By contrast, the IP gap remains robust and nearly identical between the pristine and surface-doped samples (Fig. 4h, l). Figure 4m presents the extracted SC gap magnitude as a function of the *d*-wave form. The extrapolated IP gap to antinode (${\Delta }_{0}^{{{{\rm{IP}}}}}$) reaches  ~ 60 meV, which is comparable to the IP gaps of other three-layered cuprates, such as Bi2223 and HgBa_2_Ca_2_Cu_3_O_8+*δ*_ (Hg1223), which has the highest *T*_c_ in cuprates (*T*_c_ = 130 K)^14,28,33,43^. In stark contrast, the energy gap in OP, which is ${\Delta }_{0}^{{{{\rm{OP}}}}} \sim 35\,{{{\rm{meV}}}}$ in the pristine sample, vanishes in the doped sample. Notably, both the gap magnitude and the FS topology remain unchanged in IP, indicating that surface doping has little effect on the doping level of the IP (see Supplementary Note 3, 4 for more discussion).

Importantly, despite minimized doping variation in the IP, the SC coherence peaks in the IP band become significantly sharper in the surface-doped sample (Fig. 4g, k). In contrast, the spectrum of OP becomes broadened, even though it is in a heavily overdoped condition. This indicates that the sample surface is damaged during the surface doping process; however, it also demonstrates that the IP is protected from the disorder transmitted through the OP, and the sharpening of the IP spectra should be intrinsic. Notably, the MDC peaks of the pristine sample are sharper than those of the surface-doped sample (Fig. 4d). The surface degradation may have reduced the spectral quality of the IP, to some extent, in the surface-doped sample. Considering such adverse conditions, this intrinsic sharpening is particularly striking. Here, we note that well-defined quasiparticle peaks remain observable, while broadened, in the surface-doped OP (Supplementary Note 6). This indicates that the OP is not severely damaged to the extent that would suppress superconductivity; the gap closure after surface doping is not due to a surface-damage effect but to intrinsic doping.

In the heavily underdoped cuprates, it is established that the coherent peaks, observed around the nodal region, vanish across the antiferromagnetic zone boundary (AFZB) toward the antinode. This has been revealed by both ARPES^8,10,23,24,26,44^ and STM measurements^45^. Notably, this behavior is also observed in the band of the underdoped inner CuO_2_ plane in the optimally doped three-layered cuprates^28,43^, including Hg1223 with the highest *T*_c_ in cuprates (*T*_c_=130 K)^43^. This feature is also confirmed in the underdoped IP band of the current pristine sample (colored arrows in Fig. 4g). Intriguingly, however, the surface-doped IP exhibits coherent, sharp peaks in the same momentum region (colored arrows in Fig. 4l).

One may wonder whether a small increase in the carrier concentration of the IP, induced by surface doping, could be responsible for the enhanced coherence. To explore this, we compare the IP of F0223 to that of Bi2223, a more familiar trilayer cuprate^28^ (Supplementary Fig. 6). As shown in the Fermi surface plots (Supplementary Fig. 6a), the IP of Bi2223 has a substantially higher carrier concentration than the IP of the surface-doped F0223, yet its coherence peak rapidly disappears upon crossing the AFZB (Supplementary Fig. 6b).

This comparison indicates that a subtle increase in carrier concentration is insufficient to account for the enhanced coherence observed in the surface-doped IP. Therefore, our observations suggest that metallic screening by the OP layers plays a central role in enhancing the coherence in IP. While this supports our interpretation, F0223 and Bi2223 are distinct material systems, and their IPs may not behave identically with doping. A more definitive test would involve devising a method to tune the IP doping while keeping the OP carrier concentration fixed within a single material and tracking the resulting evolution of coherence. However, such control is currently challenging with our surface treatment technique and remains a subject for future study.

With the metallic screening mechanism in mind, the enlarged Bogoliubov band (or an extended portion of the Fermi surface that contributes to superconductivity) implies a substantial increase in superfluid density; therefore, the SC phase stiffness is strengthened. Consequently, a large pairing gap (${\Delta }_{0}^{{{{\rm{IP}}}}} \sim 60\,{{{\rm{meV}}}}$) and strong phase stiffness are simultaneously realized in the single CuO_2_ sheet.

It is known that even a single-layer cuprate can reach a high *T*_c_ of  ~ 100 K, as exemplified in HgBa_2_CuO_4+*δ*_ (Hg1201)^46^. Notably, Δ_0_ of Hg1201 is only  ~ 40 meV^15^, which is 1.5 times smaller than that of the IP of F0223 (${\Delta }_{0}^{{{{\rm{IP}}}}}\, \sim 60\,{{{\rm{meV}}}}$). Further importantly, while Hg1201 is known as a relatively clean system with minimal disorder, the IP in our surface-doped samples may actually be even cleaner. This is supported by NMR measurements on Hg1201^4^ and F0223^34^, showing that the NMR spectral peak of IP for F0223 is sharper than that of Hg1201. This indicates that the pristine IP of F0223 is less disordered than that of Hg1201. Therefore, the surface-doped IP of F0223 is expected to reside in a cleaner environment, as the metallic OP layers provide enhanced screening of disorder. This may be manifested by more enhanced coherence observed in IP of F0223 than in Hg1201^15^, as evidenced by sharp quasiparticle peaks across the entire Fermi surface.

Although the *T*_c_ of the surface-doped state is not determined, these features open the possibility that the system with a single clean IP shielded by metallic layers with dense carriers may be capable of reaching a *T*_c_ well above 100 K. Note that more heavily doped samples of F0223 indeed show *T*_c_ ~ 120 K^3,34^. Although they are polycrystalline, they may be in a condition similar to that of our surface-doped state. The synthesis of a single crystal with such a high doping level has not yet been achieved; if successful, it would allow both *T*_c_ and ARPES measurements, offering a compelling direction for future research.

**Fig. 5:**
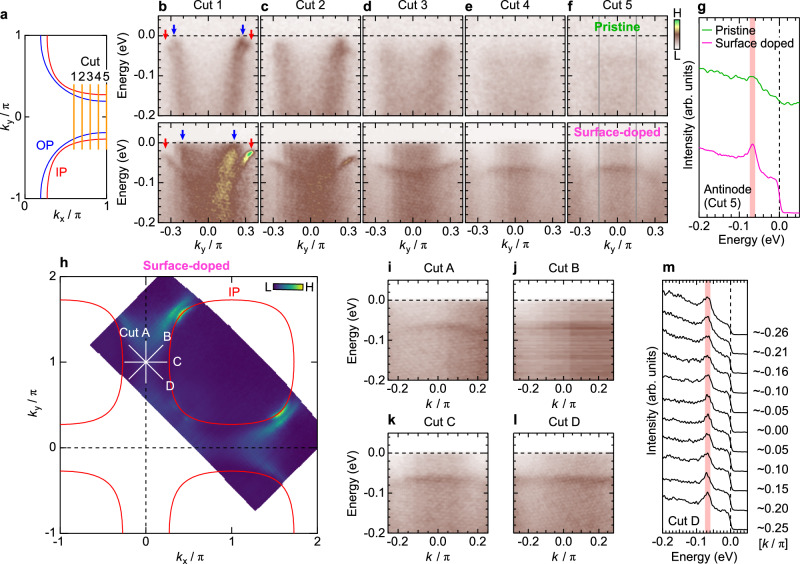
Emergence of widely extended coherent flat band in the surface-doped three-layer F0223. **a** Schematic FS of three-layered F0223. Orange lines indicate the momentum cuts used for (**b**–**f**). **b**–**f** Comparison of the band dispersions of the pristine (upper row) and the surface-doped (bottom row) sample, along the momentum cuts indicated in (**a**). IP and OP bands are marked by red and blue arrows in (**b**), respectively. **g** Integrated EDCs of the pristine and the surface-doped F0223 at the antinode. The integrated momentum window is chosen to [-0.15, +0.15] *k*_*y*_/*π* (gray lines in (**f**)). Each curve is normalized by the energy window of [-0.2, 0] eV. **h**, Fermi surface of the surface-doped F0223. Red line indicates a FS contour of IP estimated by the tight-binding method. **i**–**l** ARPES band dispersions obtained from the momentum cut indicated in (**h**) (Cut A to D; white lines). **m** EDCs, corresponding to panel (**l**). Coherent peaks are marked by a red line, exhibiting a flat band.

The simplest setting of a single CuO_2_ layer per unit cell without disorder will provide a model case to compare the microscopic theory for the pairing mechanism, which does not include the disorder effect. We further observe a pronounced change in the IP band, particularly near the antinode, due to the SC coherence enhancement. Figure 5b–f compare the energy-momentum dispersions for both the pristine and surface-doped crystals, taken along the momentum cuts labeled as Cuts 1–5 (orange lines in Fig. 5a). In the pristine sample, as one moves from Cut 1 toward Cut 5 (approaching the antinode), both the IP and OP bands become blurred and weak in intensity, reflecting the pseudogap-induced spectral damping characteristic of underdoped cuprates.

In contrast, the IP band in the surface-doped sample remains sharp and well-defined even at the antinode (Cut 5). Meanwhile, the OP band shifts above *E*_F_ around (*π*, 0), consistent with a Lifshitz transition into the heavily overdoped regime^11,38,41^. Interestingly, the band dispersion of the coherent peak is much shallower than that before doping (for example, compare panels for Cut 3 in Fig. 5d). This indicates that the coherent peak dispersion is highly renormalized. To quantify the surface-doping effect on the IP band, we integrated the EDCs over a defined momentum window (marked by gray lines in Fig. 5f) for both samples and compared them in Fig. 5g. In the pristine sample, only a hump-like feature is observed around −65 meV in the IP band. In the surface-doped sample, a sharp peak with almost the same width as that of the SC coherent peak near the node emerges at the same energy position as the hump in the pristine sample. Remarkably, the coherent peaks show a very flat band near the Brillouin zone edge. This is clearly observed along the momentum cut on the zone boundary (Fig. 5f).

We found that the coherent flat band is extended two-dimensionally over a wide momentum range around (*π*, 0). Figure 5h shows the FS mapping of the surface-doped F0223. We select four particular momentum cuts (Cuts A–D, indicated by white lines) centered at (*π*, 0) and extract the corresponding energy-momentum dispersions in Fig. 5i–l. These clearly exhibit coherent peaks and isotropic flatness; the coherent flat band of IP extends two-dimensionally over a wide momentum space around (*π*, 0). This demonstrates that SC coherent electrons at (*π*, 0) are spatially localized.

One might consider that the flat band is induced by surface degradation and is therefore unrelated to the intrinsic electronic properties of cuprates. We note that this possibility is unlikely, given our observation that the flat band is already present in the pristine samples. Although the intensity is very weak in the pristine sample, a flat-band-like feature similar to that of the surface-doped IP can be identified at the same binding energy. This feature becomes more evident in the curvature plots, which enhance the visibility of spectral peaks (Supplementary Note 8). Notably, the peak position remains unchanged upon surface doping, while coherence, as reflected in a higher peak intensity and a narrower linewidth, is greatly enhanced. We therefore conclude that the coherent flat band near (*π*, 0) in the surface-doped IP is not due to an extrinsic effect but an intrinsic feature of cuprates, which becomes particularly pronounced when disorder effects in the CuO_2_ layers are suppressed.

These findings suggest that spatially localized coherent electrons emerge only when disorder is effectively screened from the dopant layer—specifically, by locating the CuO_2_ plane between metallic layers with dense mobile carriers—thereby mitigating the pseudogap-induced spectral damping. These results may reflect a competition between electron coherence and the incoherent density wave, such as CDW^35,47–51^ and PDW^52–54^, and its sensitivity to the disorder induced by the dopant layer. Previous studies have revealed competition through variations of the doping level in the CuO_2_ layer^8,55^. Our results offer an approach to investigating competition by tuning the screening of the CuO_2_ layer. Importantly, this approach may realize an ideal system that can be directly compared with an idealized theory, free from disorder effects. This provides a promising avenue for identifying the origin of the pseudogap—a key issue for understanding the microscopic physics underlying underdoped cuprates.

## Discussion and outlook

In this study, we demonstrate, using crystals of five-layer cuprates, that a large doping (or potential) difference between CuO_2_ layers substantially suppresses interlayer hybridization, leading to a nearly complete decoupling of their electronic states, in which the IPs remain superconducting while the OPs are metallic, with no detectable SC proximity effect in the metallic layers. This conclusion is supported by our model calculations, which show negligible interlayer band mixing.

This observation prompted us to develop a physically motivated strategy to explore how interlayer decoupling influences the superconducting properties of cuprates via in situ surface doping. We thereby realized an ideal manifestation of this mechanism in a three-layer system: single inner CuO_2_ layers exhibiting a large SC gap—standing as one of the largest in the cuprate family—sandwiched between metallic sheets with dense mobile carriers that effectively screen disorder from the dopant layers. The strategy behind our results is an interlayer decoupling, achieved through excess doping of the outer layers, which creates a large doping-level (or potential) difference between layers and prevents orbital hybridization.

This system offers an experimentally accessible, nearly ideal platform for directly validating long-standing microscopic theories of pairing, which have been hindered by disorder. The large doping (or potential) difference between layers localizes the electronic wavefunctions within each CuO_2_ layer, minimizing proximity-induced mixing. Intriguingly, we find that heavily overdoped metallic OPs significantly enhance the SC coherence in the IP, overcoming the damping effect of the pseudogap states. This enables a simultaneous realization of strong pairing and strong phase stiffness in a single CuO_2_ layer. Estimating the *T*_c_ of the surface-doped state—or, if successfully synthesized, the *T*_c_ of single crystals under similar conditions—will be a key future step to further validate and quantitatively refine the present conclusions and their implications for multilayer cuprates.

Coherent flat bands around the Brillouin zone edge demonstrate that the spatially localized coherent electrons with a specific momentum (*π*, 0) emerge in a screened, clean CuO_2_ plane. These findings may indicate the partial development of strongly bound coherent pairs among (*π*, 0) electrons, competing with the pseudogap state. The results demonstrate that the purity of IPs can be tuned by the metallic conductivity of OPs, thereby introducing the degree of screening as a physical parameter in cuprate research. This parameter would be connected to other quantities. One possible quantity is the width of the energy-gap distribution obtained from the gap-map histogram in STM measurements^1^, which is known to be sensitive to disorder^2^. If an inner layer is effectively screened from the random potential induced by the dopant layer, the electronic states in that layer should become more homogeneous. In such a clean layer, the inhomogeneity of the gap state in the local density of states (LDOS) would likely be suppressed or even eliminated. Specifically, the histogram of the gap map would become sharper. The width of this gap distribution, therefore, could serve as a measure reflecting the “degree of screening”. Another relevant quantity is the width of the quasiparticle peak observed by ARPES, which is proportional to the electron scattering rate and thus should be sensitive to disorder. As a more homogeneous state is realized, the scattering rate decreases, leading to a sharper quasiparticle peak. Therefore, the peak width (or scattering rate) provides another possible quantitative indicator of the “degree of screening”. This research direction may help resolve the reality-theory mismatch, which has long been a major obstacle to constructing microscopic pairing mechanisms in high-*T*_c_ cuprates.

Our results provide several further implications. In the study of artificial heterostructures, superconductors—including cuprates—are often used as a substrate source to induce SC proximity effects^19,56^. The present results illustrate that not only the SC coherence length, as frequently argued, but also the degree of orbital hybridization between the superconductor and other materials (such as topological insulators) is crucial^57^. Our findings also suggest an explanation for why Y-Ba-Cu-O compounds are relatively clean systems among double-layered cuprates, as revealed by the sharp peak in NMR spectra^4^. This is mainly because the CuO_2_ layers are sandwiched by metallic CuO chains^58,59^, which can effectively screen disorder effects.

We also point out that the SC gap in Y-Ba-Cu-O emerges in the chains only at specific momentum points where the two-dimensional FS of the CuO_2_ layers intersects with the one-dimensional FS of the CuO chains^60^. This occurs precisely because the electrostatic potentials of the CuO_2_ layers and CuO chains align, allowing the hybridization between them. As a result, the proximity effect between the CuO_2_ layers and the chains is activated, leading to the emergence of an SC gap in the momentum region where the hybridized bands intersect. Our results open an avenue for designing disorder-resilient, high-*T*_c_ materials based on engineered electronic screening and control of hybridization.

## Methods

The single crystals of the five-layer compound Cu1245 (*T*_c_ = 78 K) and the three-layer F0223 (*T*_c_ = 100 K) were grown by a high-pressure method. Specifically, they were grown between 1000 ^∘^C and 1200 ^∘^C under a pressure of 4.5 GPa without an intentional flux^61^. Out-of-plane X-ray diffraction (XRD) measurements were carried out in the Bragg-Brentano geometry on the plate-like single crystals. Only (00*l*) reflections corresponding to a single *c*-axis lattice parameter were observed, demonstrating the absence of mixed-layer phases and confirming the phase purity of the samples. The measurements also confirmed that the *c*-axis was oriented perpendicular to the crystal surface. The *T*_c_ was determined by the onset of SC diamagnetism using a dc SQUID magnetometer. ARPES experiments were carried out at the beamline I05 of Diamond Light Source and the beamline BL5-2 of Stanford Synchrotron Radiation Lightsource. Cu1245 and pristine F0223 were in-situ cleaved and measured under a vacuum better than 8 × 10^−11^ Torr. Surface doping of F0223 was achieved by exposing the cleaved crystal surface to the air leak of  ~ 3 × 10^−9^ Torr in the ARPES measurement chamber^39^. ARPES measurements were performed using a photon energy of *hν* = 55 eV. The sample temperature was maintained at the lowest temperature (*T* ~ 7 K) during the surface-doping procedure and all subsequent measurements. The total energy resolution was set to  ~ 12 meV.

## Supplementary information

Supplementary Information

Transparent Peer Review file

## Data Availability

The data that support the findings of this study are available from the corresponding authors upon request.
