# Supplementary material for: Superconducting coherence boosted by outer-layer metallic screening in multilayered cuprates
Source: Nat Commun. 2026 Jul 14;17:8630. doi: 10.1038/s41467-026-75295-z (PMC13487180; doi:10.1038/s41467-026-75295-z)
Supplement: Supplementary file 1 [file 41467_2026_75295_MOESM1_ESM.pdf]

Supplementary Information: Superconducting coherence boosted by outer-layer metallic screening in multilayered cuprates

Junhyeok Jeong,[1] Kifu Kurokawa,[1] Shiro Sakai,[2] Tomotaka Nakayama,[3] Kotaro Ando,[3] Naoshi Ogane,[3] Soonsang Huh,[1] Matthew D. Watson,[4] Timur K. Kim,[4] Cephise Cacho,[4] Chun Lin,[5] Makoto Hashimoto,[5] Donghui Lu,[5] Takami Tohyama,[6] Kazuyasu Tokiwa,[3,*] and Takeshi Kondo[1,7,†]

[1] *Institute for Solid State Physics, the University of Tokyo, Kashiwa, Chiba 277-8581, Japan*

[2] *Faculty of Science and Technology, Sophia University, Tokyo 102-8554, Japan*

[3] *Department of Applied Electronics, Tokyo University of Science, Tokyo 125-8585, Japan*

[4] *Diamond Light Source Ltd, Harwell Science and Innovation Campus, Didcot, OX110DE, U.K.*

[5] *Stanford Synchrotron Radiation Lightsource, SLAC National Accelerator Laboratory, 2575 Sand Hill Road, Menlo Park, CA 94025, U.S.A.*

[6] *Department of Applied Physics, Tokyo University of Science, Tokyo 125-8585, Japan*

[7] *Trans-scale Quantum Science Institute, The University of Tokyo, Bunkyo-ku, Tokyo 113-0033, Japan*

(Dated: July 7, 2026)

[*] Corresponding author: Mail address: tokiwa@rs.tus.ac.jp

[†] Corresponding author: Mail address: kondo1215@issp.u-tokyo.ac.jp

NOTE 1: Superconducting critical temperatures of the single crystals used in our study, determined by the Meissner effect

In this study, we use single crystals of the five-layer cuprates (Cu,C)$Ba_2Ca_4Cu_5O_y$ (Cu1245) and three-layer cuprates $Ba_2Ca_2Cu_3O_6(F,O)_2$ (F0223) with superconducting (SC) critical temperatures (T_c) of 78 K and 100 K, respectively. To determine the T_c values, we measured the magnetic susceptibilities by a dc SQUID magnetometer (Quantum Design MPMS). Supplementary Figs. 1a, b show the magnetic susceptibility curves for the single crystals of Cu1245 and F0223 we used for ARPES measurements. The T_c are estimated as $T_c = 78$ K and $T_c = 100$ K, respectively, from the onset of the SC diamagnetism (marked by the colored arrows).

NOTE 2: Model calculations to evaluate the superconducting proximity effect

In the main text, we argued that the interlayer coupling between the outer (OP) and inner (IP) CuO_2 layers is weak, and consequently, the SC proximity effect from the IPs on the OP is also negligible for a five-layer Cu1245 and a surface-doped three-layer F0223.

We demonstrate this using a tight-binding model calculation for a system with a five-layered structure. The Hamiltonian describing the multilayer system is written as follows [1]:

$$\begin{aligned} H = &\sum_{l=1,\cdots,5} \sum_{\mathbf{k}} \left[\epsilon_l(\mathbf{k}) \sum_{\sigma} c^\dagger_{kl\sigma} c_{kl\sigma} - \Delta_l(\mathbf{k}) c_{kl\uparrow} c_{-kl\downarrow} + \text{h.c.} \right] \\ &+ \sum_{l=1,\cdots,4} \sum_{\mathbf{k}} \left[V_l \sum_{\sigma} c^\dagger_{kl\sigma} c_{k(l+1)\sigma} + \text{h.c.} \right], \end{aligned} \tag{1}$$

where $c^\dagger_{\mathbf{k}l\sigma}(c_{\mathbf{k}l\sigma})$ is a creation (annihilation) operator for electrons with momentum $\mathbf{k}$ and spin σ on the layer l. For the bare band dispersions $\epsilon_l(\mathbf{k})$ we take the tight-binding model, $\epsilon_l(\mathbf{k}) = -2t(\cos k_x + \cos k_y) - 4t' \cos k_x \cos k_y - 2t''(\cos 2k_x + \cos 2k_y) - \mu + e_l$. Here, t, t', and t'' are the in-plane nearest-, second-nearest- and third-nearest-neighbor hoppings, μ is the chemical potential, and e_l is the layer-dependent potential. $\Delta_l(\mathbf{k}) = 0.5\Delta_{l,0}(\cos k_x - \cos k_y)$ is the layer-dependent d-wave SC order parameter, and V_l is the interlayer hopping between the nearest layers. We take fixed values for the parameters except V_l and $e_2(\equiv e_4)$ for the calculations in this study. To reproduce the ARPES data, we set t, t' and t'' as 0.27, -0.078, and 0.026 eV, respectively, and also $e_1 \equiv e_5 = 0.23$ eV for the OP potential and $\mu = -0.15$ eV for the chemical potential. $\Delta_{l,0}$ is chosen to be $\Delta_{2,0} = \Delta_{3,0} = \Delta_{4,0} = 0.048$ eV for IPs and $\Delta_{1,0} \equiv \Delta_{5,0} = 0$ eV for OPs (for the schematic illustration of the model calculation, see Fig. 3(g) in the main text). We also note that a coherent peak of cuprates observed by ARPES is

typically more than 10 meV in width; this applies to both synchrotron-based and laser-based ARPES, so it is not limited by energy resolution but rather reflects the intrinsic broadening. Hence, the spectral peak (EDC peak width) is set to 10 meV for realistic comparison with ARPES results.

In principle, the interlayer electron hopping amplitude V_l may vary from layer to layer. However, we assume that it is uniform across all layers ($V_1 = V_2 = V_3 = V_4 = V$). This is a reasonable approximation because V_l primarily depends on the distance between the CuO_2 planes, and the interplanar distances in the five-layer compound Cu1245 are nearly identical (∼3.2 Å [2]).

The unique feature of multilayer cuprates is that the doping level is different layer to layer, depending on the distance from the dopant layer [3, 4]. The corresponding multiple band splitting is reproduced by either the hopping parameter (V) or the potential (e_l) difference between layers. These parameters yield distinct physical roles: V mixes wavefunctions across layers, while e_l difference (δe_l) tends to localize them in each layer. To understand multilayer electronic states, it is crucial to estimate the upper bounds of V and δe_l and how much the wavefunction of each layer can mix. This estimation is crucial for understanding the role of proximity effect in multilayer cuprates. For this purpose, we set either δe_l or V to zero and estimate the other to reproduce the ARPES data; this provides the upper bound of δe_l and V separately.

First, we obtain the upper bound of δe_l by setting V=0. Supplementary Fig. 2 displays our model calculations for the case of zero interlayer hopping ($V = 0$ eV), with varying potential offsets e_2 ($\equiv e_4$). As $e_{l=2,4}$ increases, the IP_1 band progressively shifts away from the IP_0 band. We find that $e_{l=2,4} = 0.06$ eV provides the best agreement with the band splitting for IPs observed by ARPES (Supplementary Fig. 2b and main article Fig. 3b). We therefore estimate 0.06 eV as a practical upper bound for the potential difference between IP_0 and IP_1.

Next, we estimate the upper bound for the interlayer hopping by setting $e_{l=2,4} = 0$ eV, as shown in Supplementary Fig. 3. In this scenario, with identical potentials for the inner planes, a finite hopping V produces a splitting of the IP band into three branches. Such distinct branches are not resolved in our ARPES spectra. Therefore, we instead compare the overall separation width of the IP bands with the experimental ARPES linewidths. We find that $V = 0.02$ eV slightly underestimates the band splitting in ARPES data, whereas $V = 0.04$ eV slightly overestimates it. Thus, the actual value for V should be around 0.03 eV. To avoid the possibility of underestimation, we set $V = 0.04$ eV as the practical upper bound for interlayer hopping in our five-layer Cu1245 (Supplementary Fig. 3 and main article Fig. 3c). Even so, the OP band remains gapless at the Fermi level, even at this upper bound. We also tested larger V values. We find that the gapless feature persists up to V=0.08 eV.

In real materials, both V and δe_l are finite, and their combination is important. Here, we model this situation and confirm that even in this more realistic case, the gap in the OP band does not open by the SC proximity effects from the IP layers. For this purpose, we chose an intermediate regime between the two limits discussed above and show the results in Supplementary Fig. 4. More specifically, we fix the IP_1 at $e_2 = e_4 = 0.05$ eV and vary the interlayer hopping V. The best agreement with ARPES is found at $V = 0.01$ eV. The OP band remains gapless even when V is increased beyond the previously established upper bound ($V = 0.04$ eV).

Overall, our model calculations spanning a wide range of $e_{l=2,4}$ and V validate that inducing SC proximity gap on the OP requires an interlayer hopping much larger than the value compatible with experiments. In real materials, a significant potential difference between layers prevents hybridization and lifts the SC proximity effect. It is worth noting that such a condition is naturally realized in multilayer cuprates, where CuO_2 layers are sandwiched between dopant layers.

So far, we have set the antinodal gap size of the superconducting IPs to be 0.048 eV in calculations to match our ARPES results for five-layer Cu1245. We conclude this section by demonstrating that a much larger SC gap of IPs still fails to induce a significant proximity effect to open a gap in the metallic OP band. This argument is especially important because our ARPES experiment reveals a larger gap of 0.06 eV in the three-layer F0223.

To address this, we compare two cases in Supplementary Fig. 5: one is for the IP gap of 0.048 eV as already shown in Supplementary Fig. 3b. Here we additionally plot the one for the IP gap of 0.1 eV, which far exceeds the largest reported SC gap in cuprates (0.06 eV) [1, 5–7], including the gap for our three-layer F0223 sample. For these calculations, we keep the other parameters fixed, the potential difference between IPs as zero ($e_2 = e_4 = 0$ eV), and interlayer hopping as $V = 0.02$ eV. Even under this condition, the OP remains gapless. This indicates that the magnitude of the SC gap has no significant influence on the proximity effect. This finding is consistent with our experimental observations for the three-layer F0223, which shows no energy gap in the OP, and thus exhibits no observable proximity effect (see Fig. 4 in the main text).

NOTE 3: Comparison of F0223 with other trilayer cuprate compounds

In this study, we found an intriguing surface-doping behavior in the trilayer F0223: The OP is substantially doped into the heavily overdoped regime, whereas the IP remains almost unaffected in its doping level. This behavior is distinct from that reported for the three-layer $Bi_2Sr_2Ca_2Cu_3O_{10+\delta}$ (Bi2223) [8]. As explained below, this difference is expected to arise from the unique crystal structure of F0223, which differs from that of Bi2223.

In Bi-based systems such as Bi2223, the Bi-O layers, which serve as charge-reservoir (dopant) layers, are located above and below the apical oxygen sites of the OPs. In contrast, in F0223, the apical oxygen/fluorine layer itself acts as the dopant layer. As a result, the OPs in F0223 are more effectively carrier-doped from the dopant layer than those in Bi2223.

Furthermore, the carrier concentration of each CuO_2 plane increases as its distance from the dopant layer decreases. Therefore, the OPs are generally more heavily doped than IPs. In F0223, this doping imbalance between the OPs and IPs is further enhanced because the OPs are directly bonded to the apical oxygen/fluorine dopant layer. This unique structural configuration naturally explains why F0223 exhibits a larger potential difference between the OP and IP than Bi2223.

That said, we expect that the physics presented in our manuscript applies broadly to all multilayer cuprates, including Bi- and Hg-based compounds. In general, when the OPs—regardless of the compound—are more heavily doped and thus more metallic, their higher carrier mobility results in stronger screening of disorder in IPs. However, in most other cuprates, it is difficult to selectively dope only the outer layers in large amounts without noticeably changing the doping level of the inner layers. In this sense, while the mechanism we discuss is universal to multilayer cuprates, F0223 provides a particularly clear manifestation of intrinsic cuprate physics owing to its unique crystal structure.

Here, we also note that the interlayer relation between OP and IP in the surface-doped F0223 is distinct from that of other trilayer cuprates. The theoretical framework proposed by Kivelson *et al.* [9, 10] suggests that trilayer systems are favorable for achieving high T_c because the weak phase stiffness of underdoped layers and the weak pairing strength of overdoped layers can compensate each other through interlayer coupling. Such coupling has been experimentally observed in the form of Bogoliubov hybridization [1, 11], where band bending in the superconducting state causes a crossing between the OP and IP bands, leading to a mixing of their wavefunctions.

The results of Horio *et al.* [7] indicate that the OP in the Hg-based trilayer system, $HgBa_2Ca_2Cu_3O_{8+\delta}$ (Hg1223) is less disordered than that in Bi2223, resulting in an enhanced superconducting gap. It is therefore natural that T_c is higher in Hg1223 than in Bi2223. In general, the realization of both large phase stiffness and strong pairing strength through interlayer coupling is a well-accepted framework for achieving high T_c in trilayer cuprates.

In our surface-doped samples, however, the OP is in a normal metallic state. Consequently, no Bogoliubov back-bending occurs in the OP band, and the IP cannot hybridize with the OP. As a result, the IP becomes electronically isolated from interlayer coupling with the OPs. Nevertheless, our results demonstrate that even an isolated single IP can satisfy the favorable conditions—namely, strong phase stiffness and large pairing strength—without the need for interlayer coupling.

NOTE 4: Absence of band splitting for OPs in the surface-doped F0223

One might expect that the surface doping in F0223 induces a larger hole concentration in the top OP layer than in the bottom OP layer, and thus that the two OP bands—with different hole concentrations—would appear as distinct split features in ARPES. However, our data show no such splitting. We propose two possible explanations for these observations:

(**1**). ARPES is a surface-sensitive technique characterized by a short photoelectron escape depth. The bottom OP may simply be too deep to emit photoelectrons into the vacuum. This is plausible because the distance from the cleavage plane to the bottom OP is approximately 10.1 Å[12], whereas the probing depth of 55 eV photons is extremely short (∼4.4 Å) [13]. We note, however, that the actual probing depth can vary depending on the material and the chemical composition, so this estimate should be taken as a rough guide.

(**2**). It is also possible that oxygen atoms diffuse not only into the top dopant layer but also into the bottom dopant layer beneath the bottom OP. This would lead to overdoping in both the bottom and top OPs. In this case, the electronic bands from the top and bottom OP layers would overlap, consistent with the absence of band splitting in the ARPES data.

That said, the direct experimental verification of the above proposed possibilities, namely, identifying the oxygen deposition profile with layer selectivity—particularly for the second CuO_2 plane beneath the surface—is challenging. Accordingly, these proposed two scenarios should be regarded as speculative possibilities, rather than experimentally established facts.

Nevertheless, our central conclusion does not rely on resolving these additional bands but is based on the directly measured Fermi surface topology, which robustly demonstrates that the OP is strongly overdoped, while the IP does not show a clear change in doping level within the experimental resolution. Therefore, while our conclusion does not depend on resolving these additional bands, we consider that the absence of OP band splitting should be regarded as a source of uncertainty. Here, we refer to the related ARPES studies on surface-doped samples, which show that doping changes the band but does not introduce an additional band from deeper layers.

Over the years, numerous ARPES studies have utilized surface treatments (such as alkali-metal or oxygen deposition) to tune carrier concentrations in various classes of layered materials, including cuprates [14, 15], iron-based superconductors [16], semiconductors [17], and transition-metal dichalcogenides (TMDs) [18]. However, to the best of our knowledge, distinct band splitting originating from the differently doped deeper layers has not been observed in the vast majority of these studies.

In particular, in a systematic surface potassium-deposition study on the $YBa_2Cu_3O_{6+x}$ (YBCO) system [14], band splitting from the deeper CuO chain is not observed with 110 eV

photons, although a clear change in the FS volume is detected. We note that the distance of the deeper CuO chain from the surface in YBCO (∼11.7 Å) is comparable to our F0223 case. Additionally, in a study utilizing surface oxygen doping on $Bi_2Sr_2CaCu_2O_{8+\delta}$ (Bi2212), band splitting from the bottom CuO_2 layer (or deeper unit cell) is not observed with 21 eV photons [15]. Lastly, in a potassium-deposition study on the surface of $HfTe_2$, a layered TMD, it has been suggested that the surface-deposited potassium atoms diffuse into the bulk (intercalation) [18]. A similar diffusion process may also occur in the oxygen surface-doped case, which could account for the absence of band splitting in *in-situ* surface-doping experiments using potassium and oxygen.

The exact mechanism behind this ubiquitous absence—whether governed by short probing depth, complex matrix element effects, surface disorder broadening, or rapid dopant diffusion—remains an open question in the field of surface-doping ARPES. While systematic photon-energy-dependent measurements would be valuable for investigating this specific surface physics problem, fully resolving this methodological open question is not the focus of our study. Instead, our work focuses on the many-body physics and layer-dependent electronic structures of multilayer cuprates.

Additionally, we note that the earlier ARPES study reported an interlayer band splitting of OPs in the trilayer stack in Bi2223 [6]. However, our data does not show such splitting, not only in the surface-doped samples but also in the pristine samples. We also note that a similar splitting has not been observed in another trilayer cuprate Hg1223 [7]. These results suggest that the coupling strength between OPs varies among different cuprate compounds. The interlayer coupling may also be affected by the carrier concentration of the OPs, as it is expected to be enhanced in overdoped states. This is indeed an interesting topic, and we leave its microscopic mechanism and its implications for a future study.

NOTE 5: Determination of the doping level of the IP in F0223 and its comparison with that of another trilayer cuprate Bi2223

In this study, we find that the doping level of the IP in F0223 changes little upon surface doping. It is unlikely that the carrier doping into the IP induced by surface doping is zero. However, we estimate that the variation is very small, based on tight-binding fits to the Fermi surfaces. This conclusion was reached by determining the Fermi surface from the Fermi arc across the nodal direction. We found no noticeable shift in k_F along the Fermi arc region after surface doping. This analysis excludes the region around $(\pi, 0)$, where k_F cannot be precisely defined, in particular beyond the antiferromagnetic zone boundary (AFZB). Even with this limitation, our conclusion remains valid; namely, the doping level of the IP in F0223 is barely affected by surface doping, as discussed in more detail below.

First, we note that the pristine IP exhibits a large Fermi surface centered at (π, π), rather than a small Fermi pocket centered at $(\pi/2, \pi/2)$, although it shows a Fermi-arc-like feature. A band forming a small Fermi pocket disperses deeply down to binding energies of $\sim$1 eV at $(\pi, 0)$ [19]. In contrast, the present IP-band data show spectral features confined to an energy scale that is more than one order of magnitude smaller, on the order of 0.1 eV, at $(\pi, 0)$. This behavior is consistent with the pseudogap state in cuprates, which is associated with a large Fermi surface. Relatedly, we note that the small Fermi pocket shows a notable k_F shift near AFZB with doping variation [20]. Our current data of F0223, however, do not show this, although it exhibits an arc-like feature. Thus, our data cannot be directly compared with that of the small Fermi pockets.

We argue here that the Fermi surface determined from data near the nodal Fermi-arc region can estimate the variation in doping level with high precision for the IP bands of both the pristine and surface-doped samples, which are in the underdoped regime.

In the overdoped regime of cuprates (*e.g.*, overdoped $Bi_2Sr_2CuO_{6+\delta}$ (Bi2201) reported by Kondo *et al.* [21]), the van Hove singularity near $(\pi, 0)$ lies relatively close to the Fermi level. In this case, the Fermi-surface shape near $(\pi, 0)$ is highly sensitive to small shifts of the Fermi energy associated with doping variations, while the shift around the nodal region is much smaller. In contrast, in the heavily underdoped regime (*e.g.*, underdoped Bi2201 reported by Hashimoto *et al.* [22]), the Fermi surface shows an almost parallel shift upon carrier doping over the entire Brillouin zone; Furthermore, it has been demonstrated that doping evolution in this regime is well described by a rigid-band shift. These observations indicate that the heavily underdoped IP in the present sample can also be understood, to first approximation, within a rigid-band picture. Accordingly, the Fermi surface determined by data around the nodal Fermi-arc region provides a reasonable estimate of the doping variation.

Our conclusion of a very small doping change in IPs by surface doping is consistent with the observation that the superconducting gap near the node remains unchanged. While the superconducting gap evolution near the node in the underdoped regime remains a subject of debate [23–25], several ARPES studies on Bi2212 have revealed that the gap magnitude varies significantly with doping, roughly following the T_c value [24, 25]. Both single-crystal studies [24] and surface treatments via ozone annealing [25] similarly show that the antinodal superconducting gap (Δ_0), obtained by extrapolating the near-nodal gap to the antinode, approximately follows the superconducting dome with doping. In this context, the nearly identical Δ_0 values for the pristine and surface-doped IPs in our F0223 sample (main Fig. 4m) suggest that the doping difference is likely small.

That said, we acknowledge that the strong pseudogap effect and the presence of a flat band near $(\pi, 0)$ make it difficult to determine k_F precisely in the antinodal region, preventing

a highly accurate quantification of the doping difference. Accordingly, one may ask whether a small increase in the carrier concentration of the IP, induced by surface doping, could be responsible for the enhanced coherence. This possibility, however, can be ruled out by a direct comparison with the IP of Bi2223, a more familiar trilayer cuprate [6]. Supplementary Fig. 6a shows the Fermi surface of overdoped Bi2223 with tight-binding fits (colored solid curves). By overlaying the near-nodal k_F points of IP for the pristine and surface-doped F0223 (also shown in the inset of Fig. 4m), it becomes evident that the doping level of both the pristine and surface-doped IP in F0223 is substantially lower than that of the IP in overdoped Bi2223.

Importantly, the IP of Bi2223 has a much higher doping level; yet its coherence peak rapidly disappears upon crossing the AFZB (Supplementary Fig. 6b). This behavior stands in stark contrast to the sharp coherence peak observed in the IP of surface-doped F0223 (Fig. 5m). This comparison between F0223 and Bi2223 strongly supports our conclusion that the enhanced coherence of the underdoped IP in F0223 is driven by metallic screening from the OP layers. Unlike Bi2223—where the IP coherence peak is suppressed across the AFZB, similar to the IP in pristine F0223 and other underdoped cuprates [26, 27]—the surface-doped F0223 retains a robust coherence peak beyond the AFZB and even at $(\pi, 0)$ (Fig. 5m).

We note that the OP bands in Bi2223 exhibit bilayer splitting, and therefore, the doping level is estimated from the average Fermi surface volume. According to Luo *et al.* [6], the doping levels of the two OP bands (OP_1 and OP_2) are estimated to be $p \approx 0.22$ and 0.37, respectively, yielding an average of $p \approx 0.30$, which is comparable to that of the surface-doped F0223 ($p \approx 0.30$). Such a high doping level lies well beyond the edge of the universal T_c dome commonly observed in cuprates. Nevertheless, the OP in Bi2223 remains superconducting even in this heavily overdoped regime. Furthermore, unlike in surface-doped F0223, the IP of Bi2223 does not show enhanced coherence beyond the AFZB. This contrast may originate from the significant interlayer hybridization between IP and OP in Bi2223 [1, 6], which can transmit disorder from the OP to the IP. In contrast, the interlayer coupling in apical-F multilayer cuprates is much weaker, isolating the IP from disorder effects and thereby promoting enhanced coherence.

This line of discussion supports our main conclusion in this article that electronic coherence is well established, even in the heavily underdoped regime, in momentum regions beyond the AFZB, owing to metallic screening by the outer layers. However, it is also true that F0223 and Bi2223 are different materials. To fully clarify this point, further studies would be required; for example, by devising a method to vary IP doping while keeping the OP carrier concentration fixed, and examining the corresponding evolution of coherence in the IP.

NOTE 6: Well-defined quasiparticle peaks observed in the surface-doped OP

It has been demonstrated that exposing the cleaved surface to the small air-leak condition at low temperature significantly increases the hole concentration, as reported by Palczewski *et al.* [15]. Importantly, the evolution of the superconducting gap under such surface treatments shows no evidence of severe surface degradation that could destroy superconductivity.

Nevertheless, the air leak may still cause some degree of surface degradation. To address this concern, we demonstrate below in detail that well-defined quasiparticle peaks remain observable in the surface-doped OP, indicating that the OP is not severely damaged to a level that would suppress superconductivity. Furthermore, we quantitatively evaluate the extent to which the "surface-doped" OP was degraded compared with the "pristine" OP by comparing their spectral peak widths, which correspond to the inverse of the quasiparticle lifetime. We find that the peak of the surface-doped OP is only about 45% broader than that of the pristine OP, and the linewidth remains within the range reported for cuprates in the literature. Therefore, it is unlikely that our surface was severely damaged enough to eliminate superconductivity.

The difficulty in evaluating the spectral peak width in multilayered cuprates arises from the fact that each spectrum is a superposition of multiple states. However, it is possible to extract the intrinsic spectral contribution of each state by fitting a model function, as described below.

The spectral intensity detected by ARPES, $I(k, \omega)$, is proportional to the spectral function $A(k, \omega)$ multiplied by the Fermi-Dirac distribution function $f_{\text{Fermi}}(\omega)$ at measurement temperature, which is then convolved by a Gaussian $f_{\text{Gauss}}(\omega)$ whose width corresponds to the experimental energy resolution. In particular, for the case of three-layer cuprates such as F0223, consisting of IPs and OPs, it is expressed as follows:

$$I(k, \omega) = [A^{\text{IP}}_{\text{coh}}(k, \omega) + A^{\text{OP}}_{\text{coh}}(k, \omega) + A_{\text{inc}}(k, \omega)] \cdot f_{\text{Fermi}}(\omega) \circledast f_{\text{Gauss}}(\omega) \quad (2)$$

Here, $A^{\text{IP}}_{\text{coh}}$ and $A^{\text{OP}}_{\text{coh}}$ are coherent parts for IP and OP, respectively, and modeled as Lorentzians. A_{inc} represents the overall incoherent contribution from both IP and OP, which is modeled as a broad Lorentzian multiplied by an asymmetric term [28].

Supplementary Figs. 7d and 7e overlay the EDCs at the nodal Fermi momentum (k_{F}) point and their corresponding fitting curves for the pristine OP and the surface-doped OP, respectively. We find a background intensity coming from another band, which is not taken into account in the above equation, at high binding energy. In particular, the background intensity in the surface-doped sample is relatively large. Since this additional band is non-dispersive, the spectrum off the k_{F} point can be reliably assumed to be the background

intensity. This is demonstrated in Supplementary Figs. 7a and 7c by overlaying the spectra at k_F and off-k_F, taken along the blue and magenta dotted lines in Supplementary Fig. 7a. The intensity difference between these two spectra is plotted in Supplementary Fig. 7e, which is used for fitting to the above function.

Supplementary Figs. 7d and 7e show the fitting results for pristine and surface-doped samples, respectively. The current focus is the spectral peak of OP, $A^{\mathrm{OP}}_{\mathrm{coh}}(k_\mathrm{F}, \omega)$, in particular, its energy width ($\Delta\varepsilon$), which is proportional to the scattering rate (or inverse of quasiparticle lifetime: $\Delta\varepsilon/\hbar = 1/\tau$). Importantly, we find a sharp OP peak not only in the pristine sample but also in the surface-doped sample, indicating that the latter's surface is not severely damaged to the extent that superconductivity would be destroyed.

By the fitting, we obtained $\Delta\varepsilon$ as $\sim$37 meV and $\sim$49 meV for the pristine and surface-doped samples, respectively (Supplementary Fig. 7f). To confirm the relevance of this value extracted from EDC analysis, we next conducted momentum distribution curve (MDC) analysis, which is commonly used to estimate physical parameters due to its simple Lorentzian shape, enabling easy fitting.

$\Delta\varepsilon = \hbar/\tau = \hbar\Delta k_\mathrm{F} \cdot v_\mathrm{F}$ can be expressed as the product of the MDC peak width at the Fermi level (Δk_F) and the Fermi velocity (v_F), which is determined from the MDC peak dispersion. Estimation of Δk_F and v_F, especially for the surface-doped OP, is demonstrated in Supplementary Figs. 7a and 7b, respectively. The $\Delta\varepsilon$ estimated via the MDC analysis is $\sim$55 meV; this is consistent with the one obtained by the EDC analysis ($\sim$49 meV) within the estimated uncertainty.

From this scattering rate analysis with both EDC and MDC, we acknowledge that the surface-doped OP shows a slight degradation, as characterized by approximately 45% increase in the spectral width compared to the pristine OP. However, a nodal $\Delta\varepsilon$ of $\sim$50 meV falls within the typical range reported in previous ARPES studies of cuprates [24, 29, 30], confirming that the quasiparticles in the surface-doped OP remain well-defined. We therefore conclude that the observed gap closure in OP by surface doping that transformed the OP into a heavily overdoped metallic state originates from heavy-carrier doping rather than surface degradation.

One may notice that the linewidth of the IP spectral peak of the surface-doped sample (Supplementary Fig. 7e) appears broader than that of the pristine sample (Supplementary Fig. 7d). We note that this does not indicate the degradation of IP after the surface-doping. This apparent increase in the IP linewidth is, rather, attributed to the increased energy separation between the IP and OP bands induced by surface doping (see Supplementary Figs. 8a and 8b, ARPES intensity maps measured along the nodal direction).

The momentum point where the EDCs are shown in Supplementary Figs. 7d and 7e corresponds to the Fermi momentum of the OP band ($k^{\mathrm{OP}}_\mathrm{F}$), where the OP spectral peak

is located near the Fermi level, while the IP peak appears at higher binding energy. In the pristine surface, the energy separation between the OP and IP peaks at this momentum is small, whereas surface doping significantly increases this separation. As a result, the IP peak shifts to higher binding energy at k_F^{OP}. In cuprates, spectral linewidths generally become broader with increasing binding energy. Therefore, the IP peak at k_F^{OP} exhibits an apparent broadening, which does not imply enhanced disorder.

Nevertheless, it is still important to compare the spectral line shape at each k_F for the IP bands (k_F^{IP}) before and after surface doping. In Supplementary Fig. 8c, we compare several energy distribution curves (EDCs) around the node of the pristine and surface-doped F0223. Each curve is extracted at k_F^{IP} and overlaid for spectra taken at a similar angle from the node ϕ (see inset of Fig. 4m). Notably, the linewidths of the IP coherence peaks for the pristine and surface-doped samples show no significant difference, indicating that no significant additional disorder is introduced into the IP by surface doping.

NOTE 7: Residual spectral weight near the Fermi level of IP in F0223

In the main Fig. 4k, several EDC curves of IP in F0223 exhibit a "step-like drop" or residual spectral weight near E_F. One may wonder whether it is valid to determine the superconducting gap from the spectral peak position of the EDC. Here, we demonstrate that such a spectral shape with a step-like drop near E_F is observed not only at the k_F point but also at momenta away from k_F, and is therefore attributed to photoemission background intensity rather than to intrinsic spectral features. Therefore, the superconducting energy gap is determined by the position of the coherence-peak maximum.

In Supplementary Figs. 9a-e, we plot EDCs at several k_F points together with a background extracted from momenta slightly away from k_F (see also Supplementary Fig. 7a). Even without intensity normalization, the background curve aligns well with the high-binding-energy tail of the EDC at k_F and exhibits a similar "step-like drop" near E_F. After background subtraction, the residual spectral weight near E_F is significantly reduced (Supplementary Fig. 9f-j).

Given that the remaining spectral weight at E_F after background subtraction is very small compared with the coherence-peak intensity, we conclude that the primary superconducting properties are governed by these coherence peaks. Accordingly, we reliably define the superconducting energy gap by the position of the coherence-peak maximum.

This residual spectral weight near E_F, which is observed over a wide momentum range (or angle range), may arise from inelastic scattering of mobile carriers in a metallic band (*e.g.*, from the outer plane) during the photoemission process. However, we leave identifying

its microscopic origin for a future study.

NOTE 8: Existence of flat-band feature near $(\pi, 0)$ in IP of the pristine F0223

In the main Fig. 5, we demonstrated that a coherent flat band appears in the surface-doped IP. One may consider the possibility that the flat band might be induced by surface degradation and is therefore unrelated to the intrinsic electronic properties of cuprates. Here, we rule out this possibility by demonstrating that the flat band is already present, albeit with weak intensity, in the pristine samples. In the surface-doped samples, this same flat band becomes significantly more coherent, as evidenced by the sharpening and enhanced intensity of its spectral peak.

In Supplementary Fig. 10, we show a comparison between the pristine and surface-doped samples. Each spectrum was measured along the Brillouin zone boundary. Although the intensity is very weak in the pristine sample, a flat-band-like feature similar to that of the surface-doped IP can be identified at the same binding energy (Supplementary Figs. 10a-d). This feature becomes more evident in the curvature plots (Supplementary Figs. 10e and 10f), which enhance the visibility of spectral peaks. Notably, the peak position remains unchanged upon surface doping, while the coherence, reflected by a higher peak intensity and a narrower linewidth, is greatly enhanced. We therefore conclude that the coherent flat band near $(\pi, 0)$ in the surface-doped IP is not an extrinsic effect but an intrinsic feature of cuprates, which becomes particularly pronounced when disorder effects in the CuO_2 layers are suppressed.

One possible origin of the flat band is that it arises from the combined effects of a large superconducting gap and collective mode coupling, both of which can flatten the band dispersion near the Fermi level. However, a more detailed theoretical analysis will be required to fully understand its underlying physics.

References

[1] Kunisada, S. *et al.* Observation of Bogoliubov Band Hybridization in the Optimally Doped Trilayer $Bi_2Sr_2Ca_2Cu_3O_{10+\delta}$. *Phys. Rev. Lett.* **119**, 217001 (2017).

[2] Hirai, M. *et al.* Unconventional Variation of T_c in the Multilayered Cuprate Superconductor (Cu, C) $Ba_2Ca_4Cu_5O_y$. *J. Phys. Soc. Jpn.* **76**, 054701 (2007).

[3] Kotegawa, H. *et al.* NMR study of carrier distribution and superconductivity in multilayered high-T_c cuprates. *J. Phys. Chem. Solids* **62**, 171–175 (2001).

[4] Mori, M., Tohyama, T. & Maekawa, S. Electronic states and superconductivity in multilayer high-T_c cuprates. *Phys. Rev. B* **66**, 064502 (2002).

[5] Ideta, S. *et al.* Enhanced superconducting gaps in the trilayer high-temperature $Bi_2Sr_2Ca_2Cu_3O_{10+\delta}$ cuprate superconductor. *Phys. Rev. Lett.* **104**, 1–4 (2010).

[6] Luo, X. *et al.* Electronic origin of high superconducting critical temperature in trilayer cuprates. *Nat. Phys.* **19**, 1841–1847 (2023).

[7] Horio, M. *et al.* Enhanced Superconducting Gap in the Outer CuO_2 Plane of the Trilayer Cuprate (Hg, Re)$Ba_2Ca_2Cu_3O_{8+\delta}$. *Phys. Rev. Lett.* **135**, 046501 (2025).

[8] Chen, H. *et al.* Doping Evolution of Nodal Electron Dynamics in Trilayer Cuprate Superconductor $Bi_2Sr_2Ca_2Cu_3O_{10+\delta}$ Revealed by Laser-Based Angle-Resolved Photoemission Spectroscopy. *Chin. Phy. B* (2025).

[9] Kivelson, S. A. Making high T_c higher: a theoretical proposal. *Physica B: Condensed Matter* **318**, 61–67 (2002).

[10] Berg, E., Orgad, D. & Kivelson, S. A. Route to high-temperature superconductivity in composite systems. *Phys. Rev. B* **78**, 094509 (2008).

[11] Ideta, S. *et al.* Hybridization of Bogoliubov Quasiparticles between adjacent CuO 2 layers in the triple-layer cuprate $Bi_2Sr_2Ca_2Cu_3O_{10+\delta}$ studied by angle-resolved photoemission spectroscopy. *Phys. Rev. Lett.* **127**, 217004 (2021).

[12] Iyo, A., Tanaka, Y., Tokumoto, M. & Ihara, H. High-pressure synthesis and properties of $Ba_2Ca_{n-1}Cu_nO_{2n}(F,O)_2$ (n=2-5) superconductors. *Physica C: Superconductivity* **366**, 43–50 (2001).

[13] Seah, M. P. & Dench, W. Quantitative electron spectroscopy of surfaces: A standard data base for electron inelastic mean free paths in solids. *Surface and interface analysis* **1**, 2–11 (1979).

[14] Fournier, D. *et al.* Loss of nodal quasiparticle integrity in underdoped $YBa_2Cu_3O_{6+x}$. *Nat. Phys.* **6**, 905–911 (2010).

[15] Palczewski, A. D. *et al.* Controlling the carrier concentration of the high-temperature superconductor $Bi_2Sr_2CaCu_2O_{8+\delta}$ in angle-resolved photoemission spectroscopy experiments. *Phys. Rev. B* **81**, 104521 (2010).

[16] Kyung, W. *et al.* Enhanced superconductivity in surface-electron-doped iron pnictide $Ba(Fe_{1.94}Co_{0.06})_2As_2$. *Nat. Mater.* **15**, 1233–1236 (2016).

[17] Chen, L. *et al.* Doping-controlled transition from excitonic insulator to semimetal in Ta_2NiSe_5. *Phys. Rev. B* **102**, 161116 (2020).

[18] Nakata, Y. *et al.* Dimensionality reduction and band quantization induced by potassium intercalation in 1T-$HfTe_2$. *Phys. Rev. Mater.* **3**, 071001 (2019).

[19] Kunisada, S. *et al.* Observation of small Fermi pockets protected by clean CuO_2 sheets of a high-T_c superconductor. *Science* **369**, 833–838 (2020).

[20] Kurokawa, K. *et al.* Unveiling phase diagram of the lightly doped high-T_c cuprate superconductors with disorder removed. *Nat. Commun.* **14**, 4064 (2023).

[21] Kondo, T. *et al.* Hole-concentration dependence of band structure in $(Bi, Pb)_2(Sr, La)_2CuO_{6+\delta}$ determined by the angle-resolved photoemission spectroscopy. *J. Electron Spectrosc. Relat. Phenom.* **137**, 663–668 (2004).

[22] Hashimoto, M. *et al.* Doping evolution of the electronic structure in the single-layer cuprate $Bi_2Sr_{2-x}La_xCuO_{6+\delta}$: Comparison with other single-layer cuprates. *Phys. Rev. B* **77**, 094516 (2008).

[23] Vishik, I. *et al.* Phase competition in trisected superconducting dome. *Proc. Natl. Acad. Sci. U.S.A.* **109**, 18332–18337 (2012).

[24] Anzai, H. *et al.* Relation between the nodal and antinodal gap and critical temperature in superconducting Bi2212. *Nat. Commun.* **4**, 1–7 (2013).

[25] Zhong, Y. G. *et al.* Continuous doping of a cuprate surface: Insights from in situ angle-resolved photoemission. *Phys. Rev. B* **98** (2018).

[26] Kohsaka, Y. *et al.* How Cooper pairs vanish approaching the Mott insulator in $Bi_2Sr_2CaCu_2O_{8+\delta}$. *Nature* **454**, 1072–1078 (2008).

[27] Kondo, T., Takeuchi, T., Kaminski, A., Tsuda, S. & Shin, S. Evidence for Two Energy Scales in the Superconducting State of Optimally Doped $(Bi, Pb)_2(Sr, La)_2CuO_{6+\delta}$. *Phys. Rev. Lett.* **98**, 267004 (2007).

[28] Ding, H. *et al.* Coherent quasiparticle weight and its connection to high-T_c superconductivity from angle-resolved photoemission. *Phys. Rev. Lett.* **87**, 227001 (2001).

[29] Ideta, S. *et al.* Energy scale directly related to superconductivity in high-T_c cuprates: Universality from the temperature-dependent angle-resolved photoemission of $Bi_2Sr_2Ca_2Cu_3O_{10+\delta}$. *Phys. Rev. B* **85** (2012).

[30] Chatterjee, U. *et al.* Observation of a d-wave nodal liquid in highly underdoped $Bi_2Sr_2CaCu_2O_{8+\delta}$. *Nat. Phys.* **6**, 99–103 (2010).

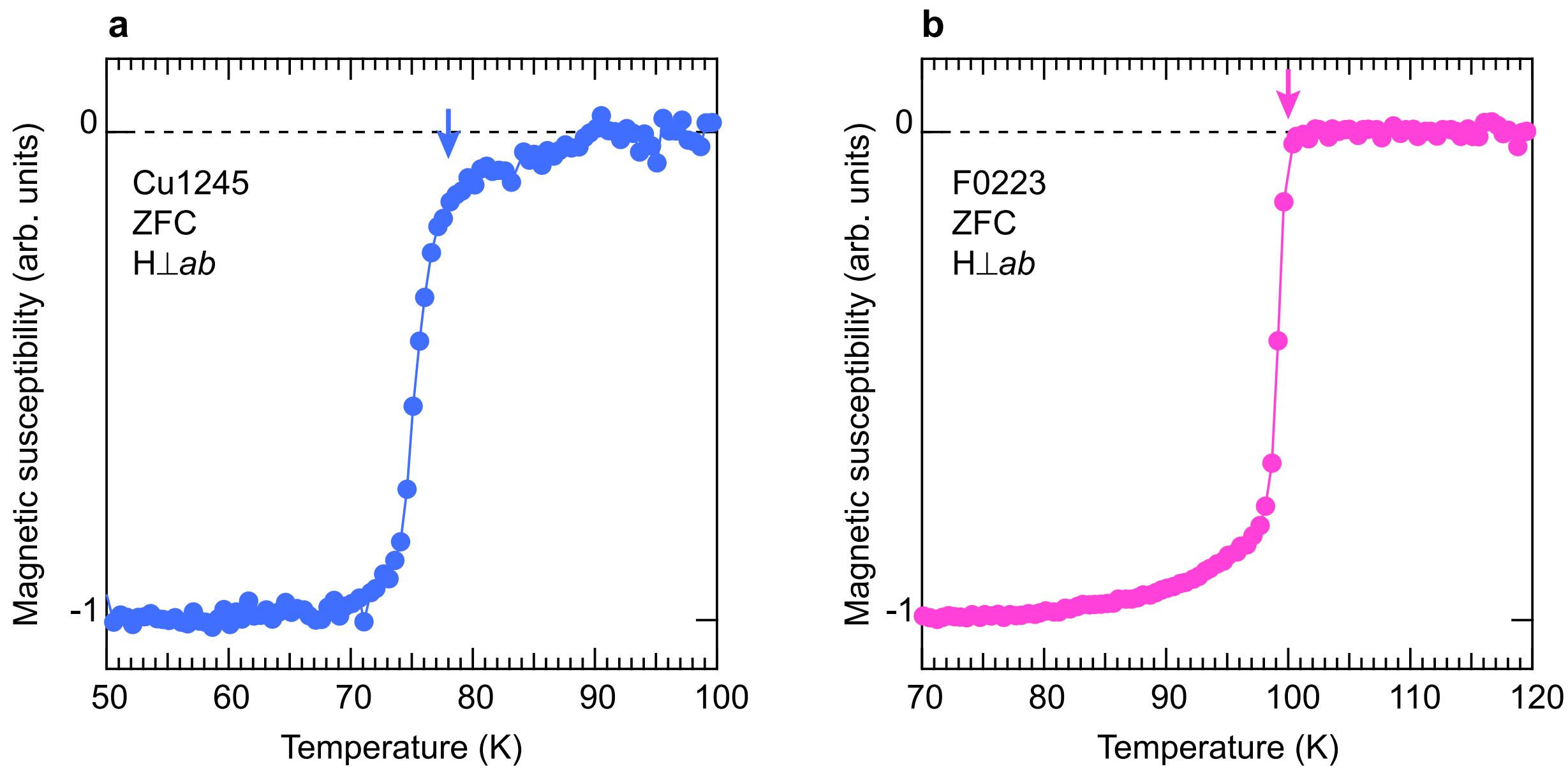

Supplementary Fig. 1. **Superconducting transition temperatures of the single crystal of Cu1245 and F0223.** **a**, Magnetic susceptibility for Cu1245 used in this study, which is normalized to the value at the lowest temperature. The T_c is estimated by the onset of SC diamagnetism (marked by colored arrow). "ZFC" stands for zero-field cooling. **b**, Similar to (**a**) but for the pristine F0223.

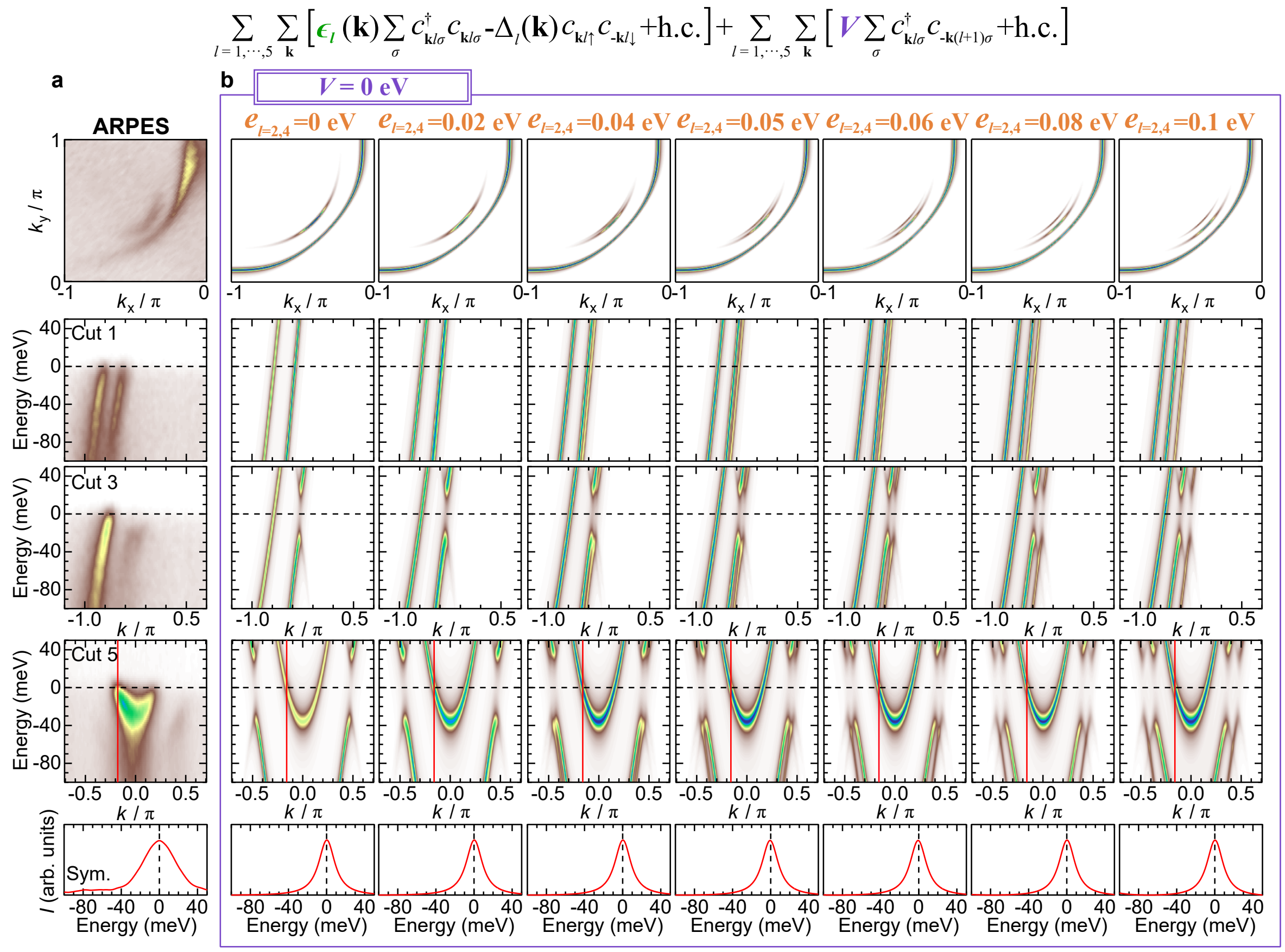

Supplementary Fig. 2. **Model calculations for Cu1245.** **a**, Cu1245 ARPES data measured at the SC state ($T = 7$ K). These are the same data shown in the main text Fig. 1. **b**, Model calculations based on (**a**), by setting a zero interlayer hopping ($V = 0$ eV), and varying IP_1 potential $e_2 = e_4$ from 0 to 0.1 eV. The best fit to the ARPES data is obtained at $e_2 = e_4 = 0.06$ eV.

$$\sum_{l=1,\cdots,5}\sum_{\mathbf{k}}\left[\epsilon_l(\mathbf{k})\sum_{\sigma}c^{\dagger}_{\mathbf{k}l\sigma}c_{\mathbf{k}l\sigma}-\Delta_l(\mathbf{k})c_{\mathbf{k}l\uparrow}c_{-\mathbf{k}l\downarrow}+\text{h.c.}\right]+\sum_{l=1,\cdots,5}\sum_{\mathbf{k}}\left[V\sum_{\sigma}c^{\dagger}_{\mathbf{k}l\sigma}c_{-\mathbf{k}(l+1)\sigma}+\text{h.c.}\right]$$

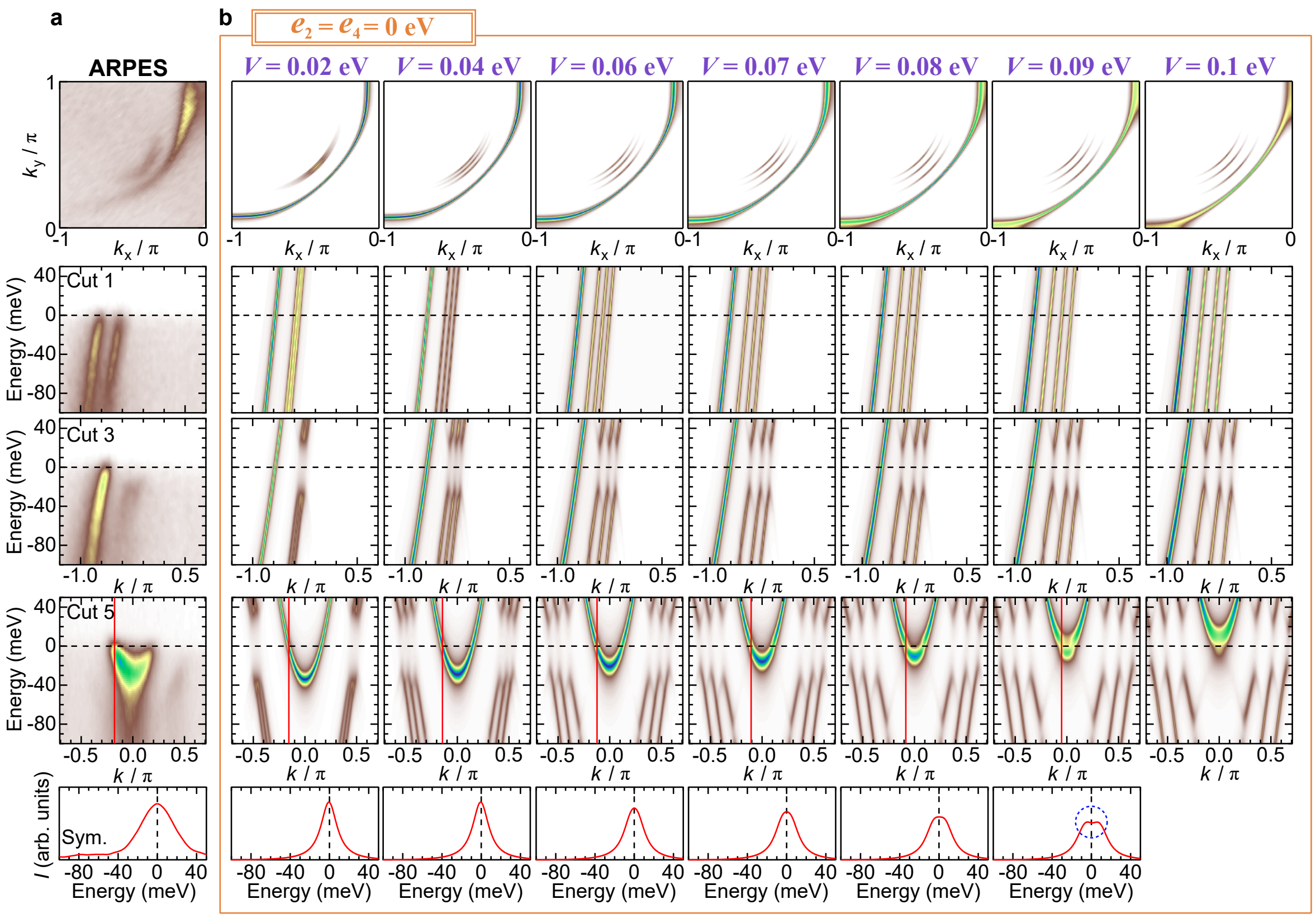

Supplementary Fig. 3. **Model calculations for Cu1245.** **a**, Cu1245 ARPES data measured at the SC state ($T = 7$ K). These are the same data shown in the main text Fig. 1. **b**, Model calculations based on (**a**), by setting a zero IP_1 potential ($e_2 = e_4 = 0$ eV), and varying interlayer hopping V from 0.02 to 0.1 eV. The best fit to the ARPES data is obtained between $V = 0.02$ eV and 0.04 eV. We take a conservative margin for the upper bound of V, to 0.04 eV. By setting $V = 0.09$ eV the OP band starts to show a moderate gap opening (blue dashed circle on the energy distribution curve (EDC) at the Fermi momentum k_{F}). At the same time, the large $V = 0.1$ eV yields a significant band splitting between OP and IP, making the OP band shift to above the Fermi energy.

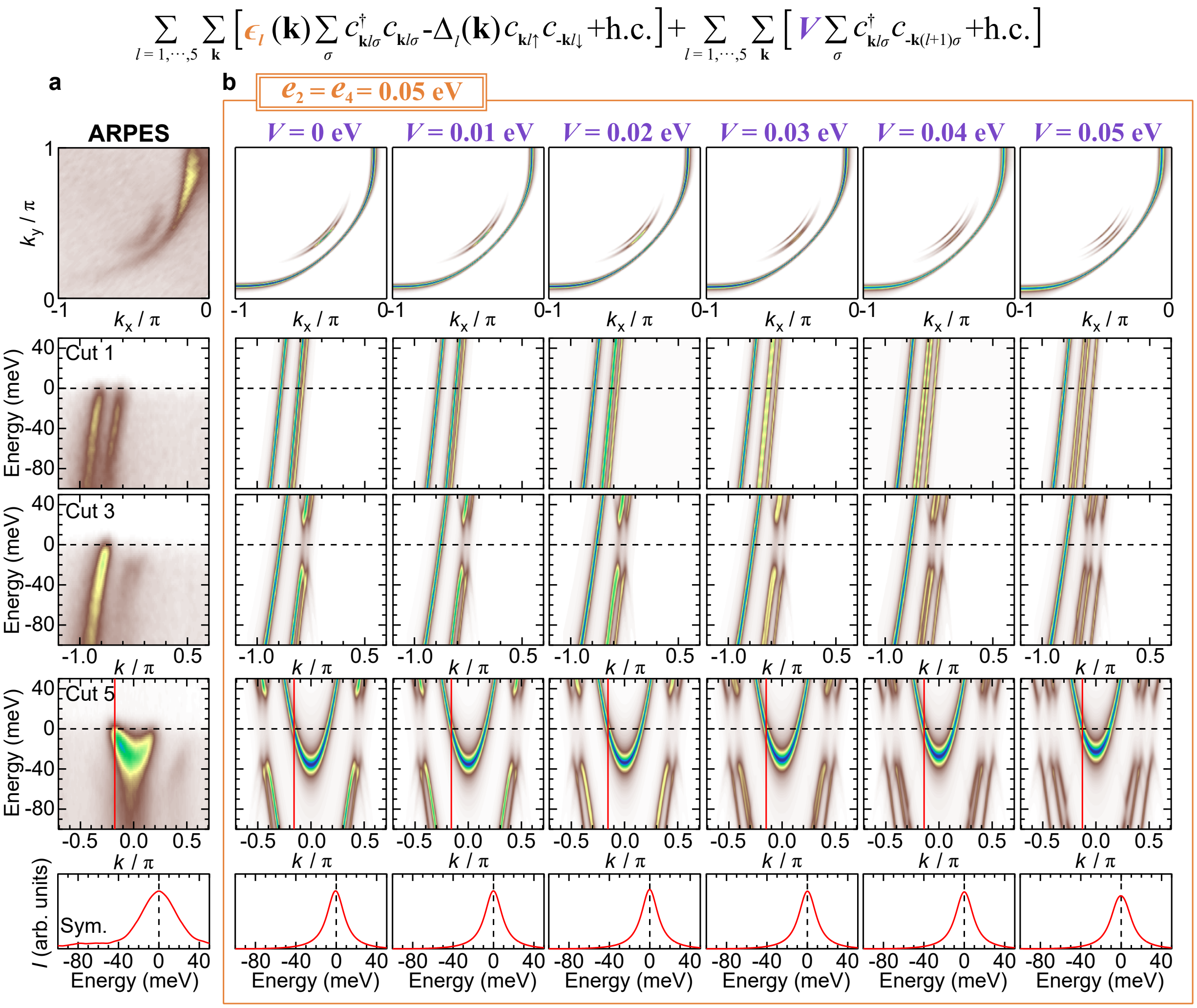

Supplementary Fig. 4. **Model calculations for Cu1245.** **a**, Cu1245 ARPES data measured at the SC state ($T = 7$ K). These are the same data shown in the main text Fig. 1. **b**, Model calculations based on (**a**), by taking an intermediate IP_1 potential below the upper bound determined from the supplementary Fig. 2 ($e_2 = e_4 = 0.05$ eV), and varying V. The best fit to the ARPES data is obtained by $V = 0.01$ eV.

$$\sum_{l=1,\cdots,5}\sum_{\mathbf{k}}\left[\epsilon_l(\mathbf{k})\sum_{\sigma} c^{\dagger}_{\mathbf{k}l\sigma}c_{\mathbf{k}l\sigma} - \Delta_l(\mathbf{k})\, c_{\mathbf{k}l\uparrow}c_{-\mathbf{k}l\downarrow} + \text{h.c.}\right] + \sum_{l=1,\cdots,5}\sum_{\mathbf{k}}\left[V\sum_{\sigma} c^{\dagger}_{\mathbf{k}l\sigma}c_{-\mathbf{k}(l+1)\sigma} + \text{h.c.}\right]$$

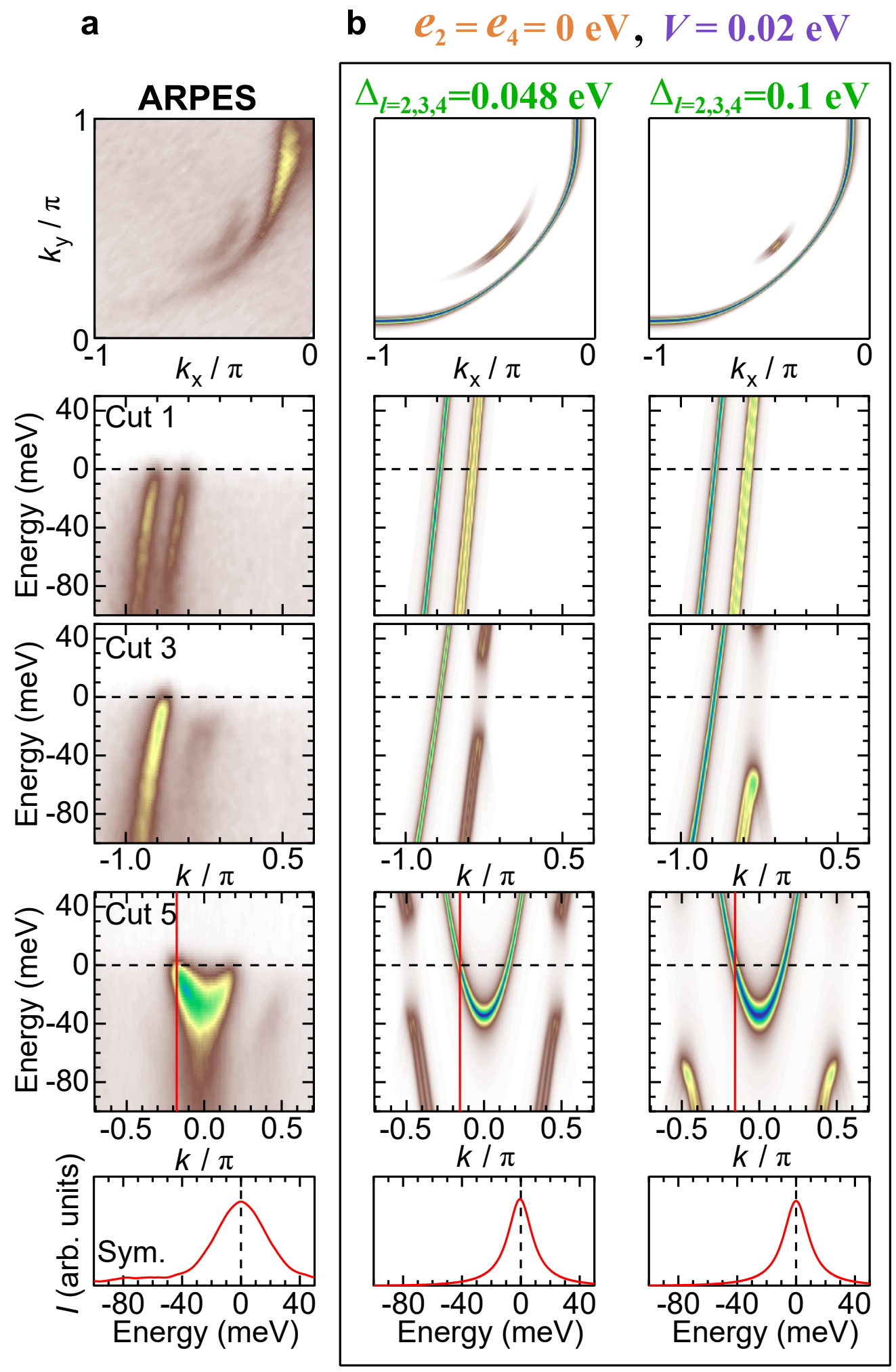

Supplementary Fig. 5. **Superconducting gap dependent calculations for Cu1245. a**, Cu1245 ARPES data measured at the SC state (T = 7 K). These are the same data shown in the main text Fig. 1. **b**, Model calculations based on (**a**), by taking the zero IP potential $e_2 = e_4 = 0$ eV and the interlayer hopping $V = 0.02$ eV, and varying the IP gap size $\Delta_2 = \Delta_3 = \Delta_4$.

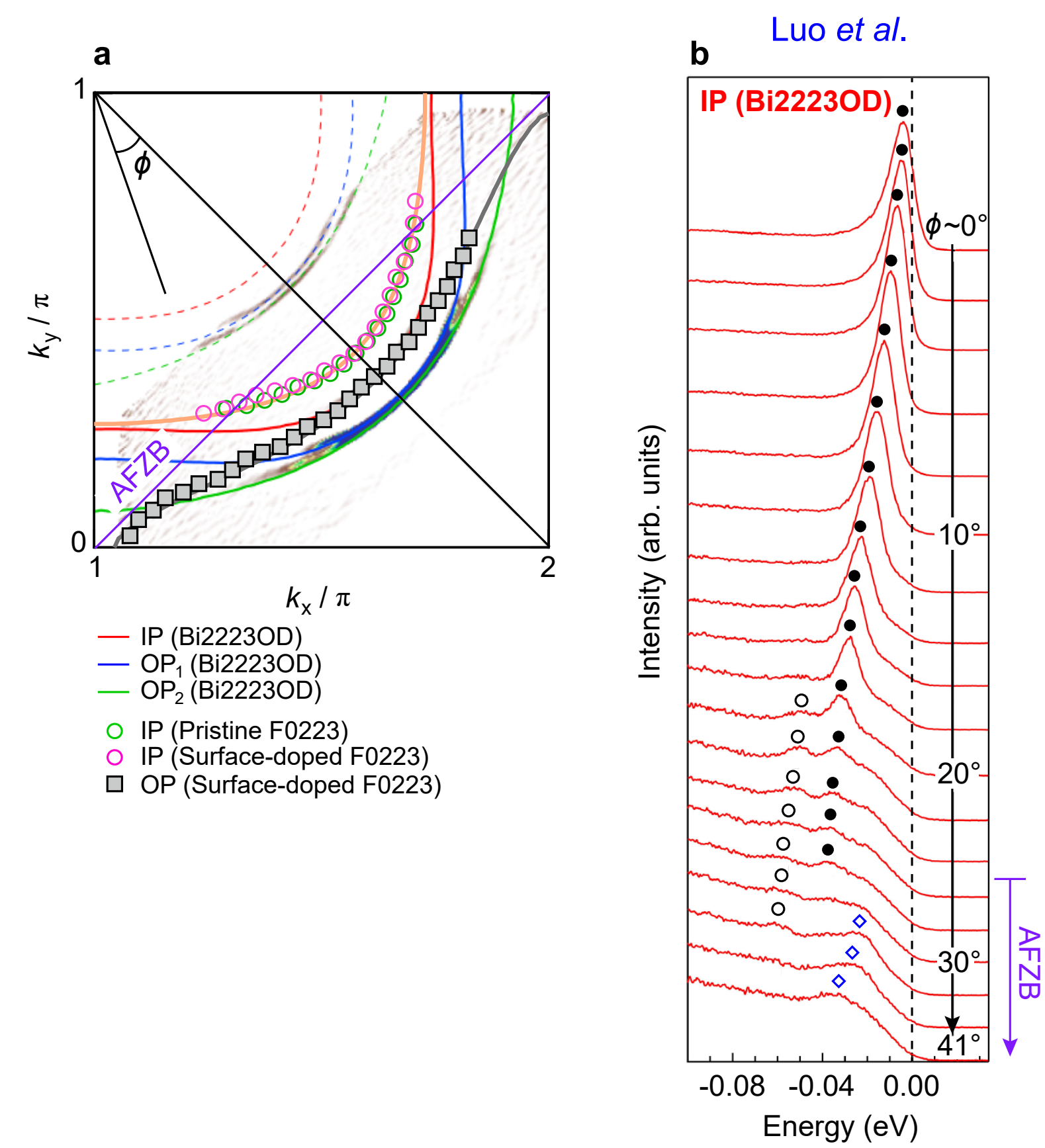

Supplementary Fig. 6. **Comparison of the Fermi surfaces of F0223 and Bi2223 a**, The Fermi surface of overdoped Bi2223 (Bi2223OD) [6], overlaid with the IP k_{F} points of pristine- and surface-doped F0223 (same data shown in the inset of Fig. 4m). The solid curves are the tight-binding fits of each band. Dashed curves represent a shadow band originating from the superstructure of Bi2223. **b**, EDCs of IP in Bi2223OD, from nodal ($\phi \sim 0°$) to antinodal direction ($\phi \sim 45°$). Black circles indicate the peak position of the IP coherence peak. Empty circles indicate the band hybridization of IP with OP. Blue diamonds are the peak positions of OP_1 (adapted from Luo *et al.* [6])

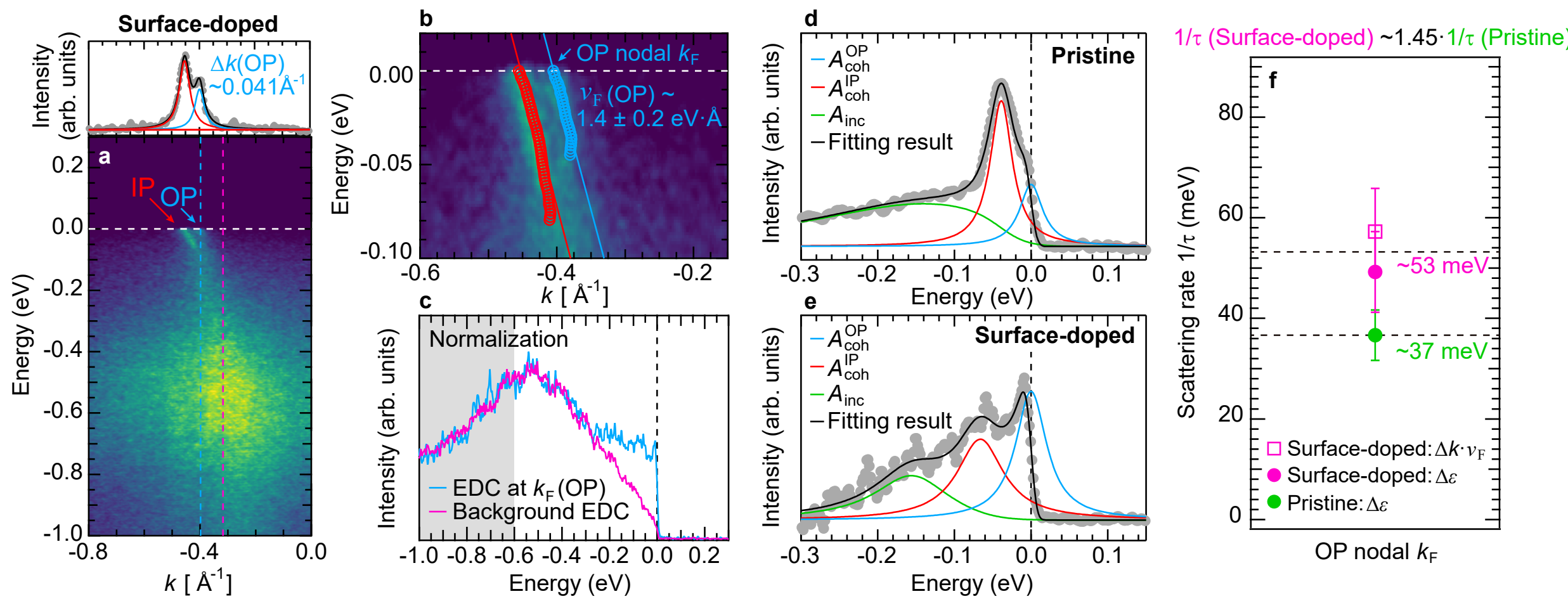

Supplementary Fig. 7. **Scattering rate analysis for OPs of F0223.** **a**, Energy-momentum dispersion of surface-doped F0223, taken along the nodal direction. The upper panel shows a momentum distribution curve (MDC) at E_{F}. **b**, Enlarged plot of (**a**). Band dispersions are obtained by peak positions of MDCs through Lorentzian fitting. The Fermi velocity v_{F} is obtained by linearly fitting the band dispersions near E_{F}. **c**, EDC at k_{F} of the surface-doped OP (blue) and background curve (magenta). Each curve is taken from the dotted line in (**a**), then normalized by the area integrated by an energy range of $[-1, -0.6]$ eV (grey shaded area). **d**, EDC fitting result for the pristine OP at nodal k_{F} point. **e**, Similar to (**d**), but for the surface-doped OP. The EDC is shown after subtracting the background shown in (**c**). **f**, Extracted scattering rates for the pristine and surface-doped OP, obtained from EDC and MDC analysis.

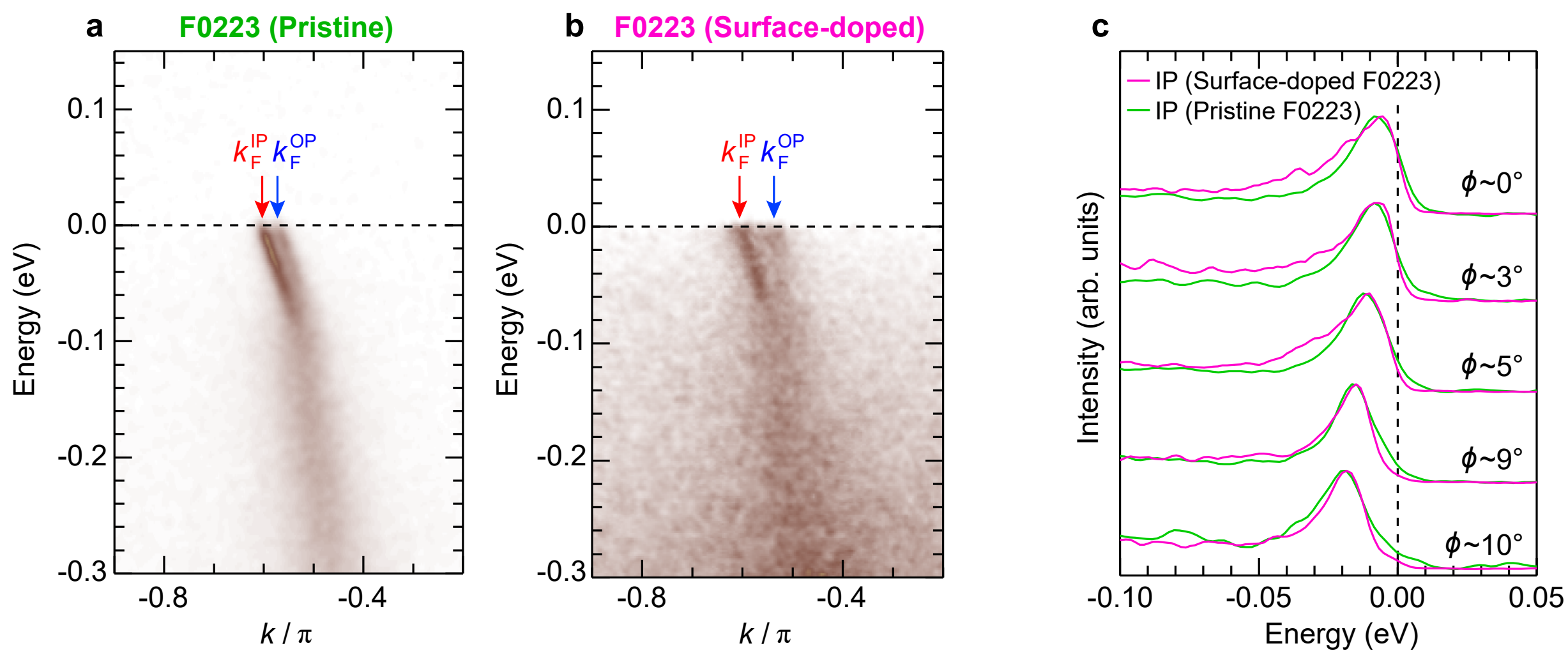

Supplementary Fig. 8. **Comparison of near-nodal EDCs of pristine- and surface-doped IP.** (a) Energy–momentum dispersion of pristine F0223 along the nodal direction. Red and blue arrows indicate the Fermi momentum (k_{F}) points of the IP and OP ($k_{\mathrm{F}}^{\mathrm{IP}}$ and $k_{\mathrm{F}}^{\mathrm{OP}}$, respectively). (b) Similar measurement to panel (a), but for surface-doped F0223. (c) Comparison of near-nodal EDCs of pristine (green curves) and surface-doped (magenta curves) F0223. Each curve was extracted from the k_{F} points of the IP. Here, ϕ is the angle measured from the node (see the inset of Fig. 4m).

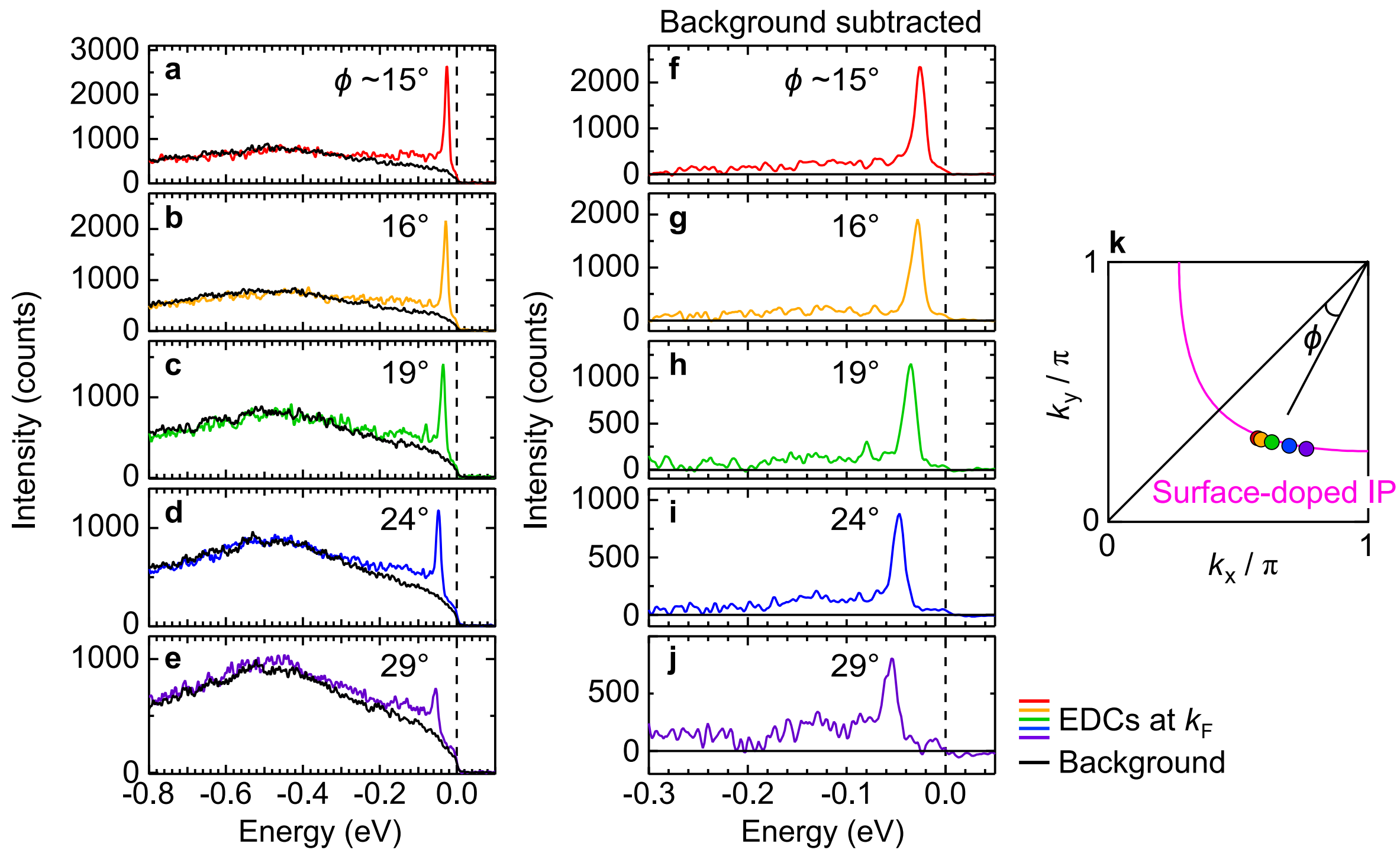

Supplementary Fig. 9. **EDCs after background subtraction for the surface-doped IP of F0223. a-e**, EDCs taken from various k_F points in the surface-doped IP, as indicated by colored circles in (**k**). These EDCs are the same curve shown in the main Fig. 4k. Black curves are backgrounds taken from the momenta slightly off from k_F. **f-j**, The same curves as in (**a-e**), but shown after the background subtraction. **k**, Schematic Fermi surface of surface-doped IP and the k_F points where the colored EDCs in (**a-e**) were taken.

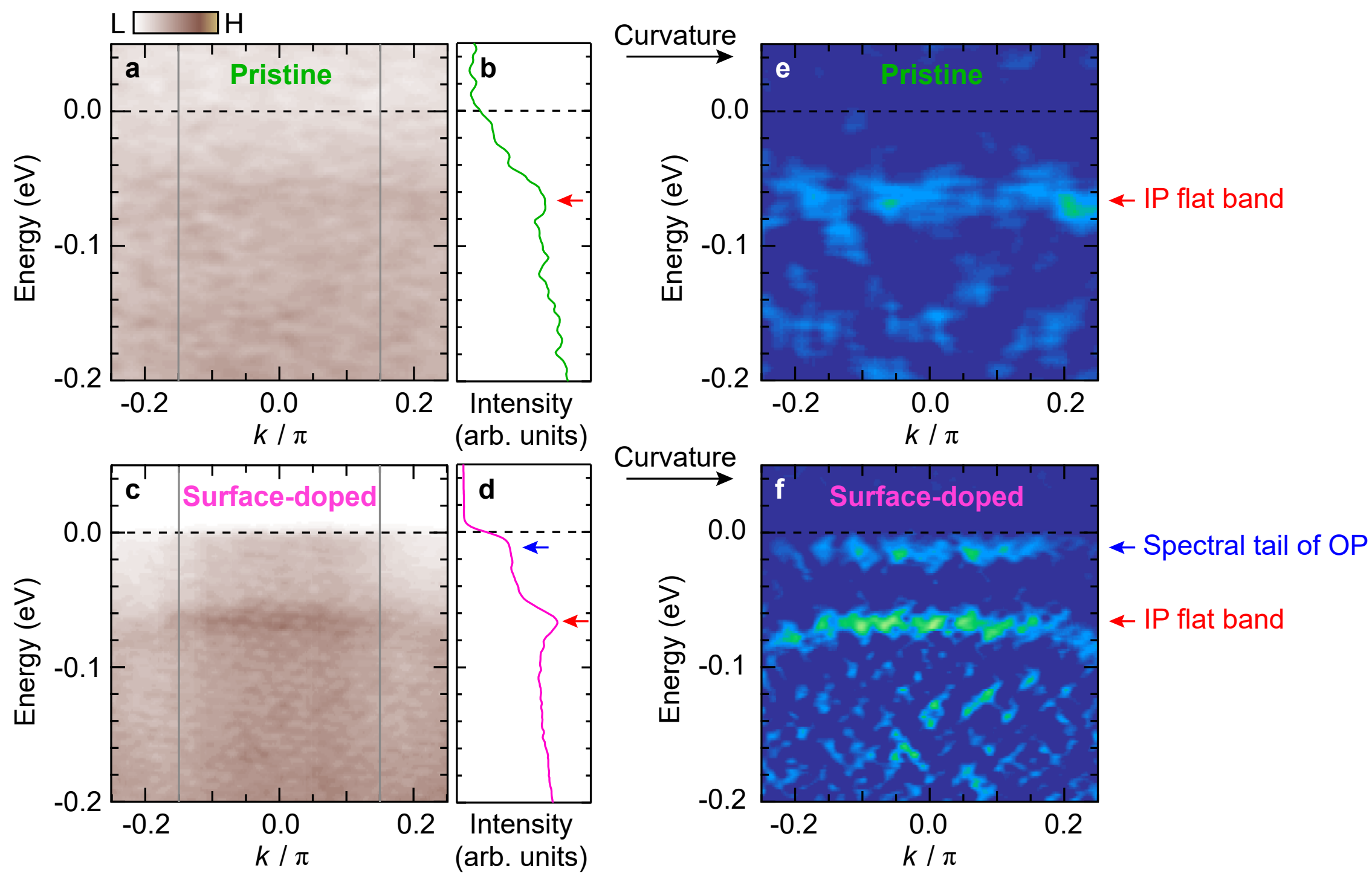

Supplementary Fig. 10. **Flat band for the pristine and surface-doped IPs of F0223. a**, Energy-momentum dispersion of the pristine F0223, taken at the zone boundary. **b**, EDC of (**a**), integrated over a momentum range of $[-0.15, 0.15]$ (k/π) (grey solid line in (**a**)). **c**,**d**, Similar data to (**a**, **b**), but for the surface-doped F0223. The data shown in (**a**-**d**) are the same as those presented in Figs. 5f-5k. **e**,**f**, The curvature plots of (**a**, **c**), revealing the existence of flat-band features (red arrows). In (f), the spectral tail of the unoccupied OP band is visible near E_{F}.
