# Supplementary material for: Superconducting coherence boosted by outer-layer metallic screening in multilayered cuprates
Source: Nat Commun. 2026 Jul 14;17:8630. doi: 10.1038/s41467-026-75295-z (PMC13487180; doi:10.1038/s41467-026-75295-z)
Supplement: Supplementary file 2 [file 41467_2026_75295_MOESM2_ESM.pdf]

nature portfolio

Peer Review File

Superconducting coherence boosted by outer-layer metallic screening in multilayered cuprates

Corresponding Author: Professor Takeshi Kondo

This file contains all reviewer reports in order by version, followed by all author rebuttals in order by version.

Version 0:

Reviewer comments:

Reviewer #1

(Remarks to the Author)
In this manuscript, Jeong et al. investigate the electronic structure of the five-layer $(Cu,C)Ba_2Ca_4Cu_5O_y$ (Cu1245) and tri-layer $Ba_2Ca_2Cu_3O_6(F, O)_2$ (F0223) cuprates—systems that have not been studied by ARPES previously. They find that, in both Cu1245 and surface-doped F0223, the inner CuO_2 plane (IP) is superconducting, whereas the outer planes (OPs) remain non-superconducting metals. In surface-doped F0223, the IP exhibits the largest superconducting gap ($\Delta \approx 60$ meV) together with a coherent Bogoliubov quasiparticle peak near the antinode. Remarkably, the IP doping level is below ~1%. The authors argue that interlayer coupling—and therefore the superconducting proximity effect—is weak, and that the highly doped OPs screen disorder from the dopant layers, rendering the IP an effectively disorder-free superconducting CuO_2 sheet. These interesting results refresh the field and provide a unique platform for probing high-Tc mechanisms in cuprates. On this basis, I believe the work meets the high standards of Nature Communications and recommend publication after minor revision. I outline a few minor comments below that could further strengthen the manuscript.
1. It is not clear why, in F0223, the OPs can be doped to ~30% while the IP remains below ~1%, in contrast to the trilayer cuprate Bi2223 with a similar structure. The authors argue that the large difference in the el reduces the interlayer hopping and enhances the screening. Why does the F0223 have a larger difference in el compared with Bi2223? Is F0223 a special case or other trilayer system, like Bi- and Hg-, that may have a similar effect when doping enough hole carriers?
2. Upon surface dosing, is only the top OP doped, or is the OP below the IP also doped sufficiently to enable effective screening? Did the author observe band splitting of the OPs after surface doping?
3. Can the author quantify the newly introduced “the degree of screening” or connect it to other quantities?
4. The sample temperature during surface doping is not specified in the manuscript.

Reviewer #2

(Remarks to the Author)
The manuscript reports ARPES measurements and theoretical calculations on multilayer-cuprates. In five-layer cuprate Cu1245, the superconducting gap is observed on the IP band, but not on the OP band. In three-layer cuprate F0223, the superconducting gap is identified on both IP and OP bands of the pristine sample. However, after exposing the cleaved surface of the sample to a small air leak in the vacuum chamber, the energy gap disappears on the OP band, but persists on the IP band. A possible surface doping is suggested and a screening effect is discussed. In addition, a coherent flat band is observed near the Brillouin zone edge of the IP. Based on these observations, the manuscript further suggests the existence of interlayer decoupling and clean single CuO2 layers, on which a simultaneous realization of strong pairing and strong phase stiffness can be realized.

I would like to mention at the outset that it is indeed important to investigate multilayer-cuprates. I also agree that multilayer-cuprates provide a special platform to look for the protected clean CuO2 planes and examine the individual roles of interlayer coupling and the presence of clean CuO2 planes, respectively. In this context, the current study may provide important information to the community of high Tc superconductivity. With that being said, I do have serious concerns on both the experiments and interpretations. Therefore, I cannot recommend the publication of this manuscript, at least, not the current version.

First, the so-called surface doping in the current study is achieved by exposing the cleaved surface to a small air leak (~3 x

10-9 Torr) in the vacuum chamber. Typically, the exposure of freshly cleaved sample surface to an air leak would destroy the surface, instead of inducing a simple doping effect. As the authors mentioned, in-situ ozone annealing is typically used to induce hole doping in cuprates. The local oxygen pressure provided by ozone annealing is significantly different from that of a small air leak. In fact, when an overdoped cuprate (e.g. overdoped Bi2212 with Tc~50K) is cleaved in vacuum, the surface layer starts to lose oxygen. Therefore, it is hard to imagine that the exposure of freshly cleaved surface to a small air leak (with the vacuum of ~3 x 10-9 Torr) can induce oxygen to the sample and even dope the sample to the extremely overdoped non-superconducting region. In this sense, it is important to experimentally demonstrate that the absence of an energy gap on the 'surface-doped' OP band is not due to sample aging effect or a degraded sample surface.

Second, the manuscript suggests that the surface OP layer is hole-doped by the oxygen and the IP layer remains nearly unchanged. Following this assumption, one would believe that the OP layer underneath the IP layer remains the same as that in the pristine sample. Therefore, the doping levels of the two OP layers would become significantly different. Thus, one should observe two sets of bands and Fermi surfaces from the OP layers, which is not the case in the current study. It is also hard to reconcile such a problem by assuming that the electronic structure of the other OP layer (underneath the IP layer) cannot be probed by ARPES. Because earlier ARPES results have demonstrated that not only the bands but also the interlayer coupling between the two OP layers can be probed by ARPES in three-layer cuprates.

Third, the current work suggests that both a large superconducting gap and strong phase stiffness are simultaneously realized in the single CuO2 sheet, pointing to an ideal platform for realizing high Tc superconductivity. One can understand that the superconducting Tc of the so-called surface doped sample cannot be easily determined by transport and magnetic measurements. However, it is straightforward to show the temperature evolution of the superconducting gap probed by ARPES. Does the superconducting gap of the surface doped sample persist to a temperature higher than that of the pristine sample?

Fourth, the sharp EDC peaks in the surface doped IP bands are indeed surprising. However, one can clearly see a step like drop at EF (e.g. several EDCs in Fig. 4k). In this case, is it still valid to claim an energy gap by the peak of the EDC?

Fifth, the observation of a flat-band-like feature is interesting. However, the understanding of its origin is important. In particular, possible extrinsic effects induced by degraded surface should be ruled out experimentally.

Sixth, the ARPES results on the five-layer Cu1245 seem to be valid. However, the bands from the IP1 and IP0 are much weaker than that from the OP. It has been reported that the relative intensity of the bands from IP and OP can be changed by different photon energies and polarizations. More systematic measurements would enhance the data quality for the quantitative gap analysis.

Reviewer #3

(Remarks to the Author)
I read with interest the manuscript "Superconducting coherence boosted by outer-layer metallic screening in multilayered cuprates" by Jeong et al, where the authors present detailed ARPES measurements on two multilayered cuprates: the five-layered system Cu1245 and the triple-layered F0223. Both compounds exhibit a novel and quite interesting electronic structure where only the bands assigned to the Inner Planes (IP) show signatures of superconductivity, and those belonging to the Outer Planes (OP) do not. The authors claim that in these systems, contrasting the more familiar and well studied multilayered systems such as Bi2223, there is no hybridization between the distinct FS in the superconducting state. Instead, they propose that the main role of the OP is to screen the underdoped IP from disorder, thereby boosting its superconducting coherence.

I think the data-quality is excellent, the manuscript is well written and the story is compelling. However I do have some concerns regarding the interpretation of the presented data that would need to be sufficiently addressed before recommending publication.

My main concern lies with the claim of the authors that in the three-layered compound F0223, after exposure of the sample surface to air, the OPs are doped to such a degree they are no longer superconducting, while crucially, the IPs remain at the same doping and only gain SC coherence due to the metallic nature of the now overdoped OPs. The authors say that the IP doping is unchanged, based on the near-nodal gapsize and Fermi momenta (as the authors are not able to identify an IP dispersion outside of the AFZB before the air exposure). I do not find this reasoning particularly convincing. While it is clear that the OP nodal k_F has changed, It seems entirely possible, and perhaps even likely for the IP doping to also be different, with this being mainly expressed by a modified gap and dispersion far away from the zone diagonal, similar to what is observed when doping Bi2201 [1](ref. (40)) and 6-layer BaCCO(F,O) [2] (ref. (7)). My issue here is that even a small increase in doping of the IP could very well lead to an increased superfluid density and a growth of the coherence peaks.

Related to this, the quality of the OP band clearly degrades after the surface doping, presumably due to disorder. If the claim is that the IP is completely protected from this disorder due to the above lying OP, one might expect the "second" OP (the one on the bottom of the surface trilayer stack) to also not be affected by this surface disorder. However, there is no sign of a splitting of OP bands after the surface dosing.

Some additional points:
Later on, the authors speculate that a decreasing gap on the OP but increasing IP coherence might raise Tc, however without any experimental evidence. This would be an opposite scenario to what is suggested in [3] (ref. (43)), where it is

actually the surprisingly large gap on the OPs of Hg1223 that is causing an increased Tc compared to for example Bi2223. In general I feel a more comprehensive discussion is warranted about the differences between the compounds presented here and the more commonly studied multilayers Bi2223 and Hg1223, both in terms of crystal and electronic structure because the intrinsic physics seems to a degree different [3,4] (refs (16,43)) as well as recent arXiv papers such as [5] and [6]).

Concerning the modelling of the 5 layer compound, where the authors explore different on-site energies for the distinct layer types to model the band structure, so as to find limiting values of the interlayer hopping V. In the supplementary information the authors say they do not consider cases where V1/=V2/=V3/=V4 for simplicity. I understand that an exhaustive modelling of all different combinations would be a lot, but a discussion of potential consequences of differing interlayer hoppings is warranted, given that the splitting between IP1 and IP0 bands is not observed, and the on-site energies between IP0, IP1 and OP are actually quite different.

[1] Kondo, T., et al., (2004). Journal of Electron Spectroscopy and Related Phenomena, 137–140(SPEC. ISS.), 663–668. https://doi.org/10.1016/j.elspec.2004.02.104
[2] Kurokawa, K. et al., (2023), 14(1), 4064. https://doi.org/10.1038/s41467-023-39457-7
[3] Horio, M., et al. (2025). Physical Review Letters, 135(4), 46501. https://doi.org/10.1103/p4c3-t34b
[4] Luo, X., (2023). Nature Physics, 19(12), 1841–1847. https://doi.org/10.1038/s41567-023-02206-0
[5] Smit, S. et al., arXiv:2506.01448 (2025)
[6] Chen, H. et al., arXiv:2508.09881 (2025)

Version 1:

Reviewer comments:

Reviewer #1

(Remarks to the Author)
In the revised manuscript, the authors have addressed all of my previous comments and suggestions. The work presents a valuable and rather unique platform for studying the intrinsic high-T_c mechanisms in cuprates, and demonstrates an effective in situ approach to tuning the doping level, which may be broadly applicable to other cuprates and complex oxides. I therefore recommend the manuscript for publication in Nature Communications in its current form.

Reviewer #2

(Remarks to the Author)
I have carefully examined all the reviewers' reports and the authors' response. This work is potentially important and the authors have spent efforts to address the issues raised by the reviewers.

The main concern shared by all reviewers is the claim of substantial different doping between the OP and IP layers. This concern is directly triggered by the the absence of band splitting. In the authors' response, two possibilities are discussed. First, the bottom OP may simply be too deep, whereas the probing depth of 55 eV photons is extremely short. Therefore, the bottom OP is not probed in the experiment. Second, both the top and bottom Ops are doped whereas the IP remains unchanged. The first explanation is indeed possible, but it remains a reasonable speculation if not demonstrated by the experiment. The second explanation is counterintuitive. It is really hard to imagine that both OP layers are doped by the surface oxygen, but the IP layer in the middle remains unchanged. Such a counterintuitive explanation really needs experimental evidence.

The authors have carried out additional data analysis to demonstrate that "the peak of the surface-doped OP is only about 45% broader than that of the pristine OP, and the linewidth remains within the range reported for cuprates in the literature. Therefore, it is unlikely that our surface was severely damaged enough to eliminate superconductivity." I appreciate the authors' efforts, and I agree that the data analysis on the OP band makes sense by itself. However, I also need to point out that the linewidth of IP changes much greater than 45% in the same data analysis (Fig. R1 d,e). It is hard to imagine that the surface degradation of IP is stronger than OP on the top.

As I mentioned in the previous report that the investigation of multilayer cuprates is important. In principle, I would be happy to see that the current study can be published in Nature Communications. Nevertheless, I would still suggest the authors to make the discussions self-consistent, and demonstrate the speculated issues, if at all possible.

Reviewer #3

(Remarks to the Author)
I appreciate the authors extensive response to the reports and some of the revisions made to the manuscript, many of which have strengthened paper. However I remain unconvinced about key aspects of the data interpretation, that are crucial to the drawn conclusions. In particular regarding the doping of the IP FS of the trilayer sample - I reiterate here that this is important because small changes in doping can very plausibly sharpen up quasiparticles.

The authors claim less than 1% change of doping on the IP FS after air exposure, based on two things - the near nodal gapsize and Fermi momenta. In their response the authors say that the bands near (pi,0) are insensitive to doping variations at low doping (due to the deep lying van Hove points) without providing evidence for this. Given that correlations can change drastically with doping, especially in this regime of low doping, a rigid band-shift model is not at all obvious to be applicable here. Furthermore the authors claim that the pristine IP FS is large and (pi,pi) centered without being able to follow the dispersions outside the AFBZ. As such it is unclear to me how the tight binding model for this data is determined.

Regarding the gapsize the authors cite two papers (“R5” and “R12”) and say that "the superconducting gap near the node varies significantly with doping in the underdoped region”. This feels exaggerated and not necessarily supported by the data shown in those papers. Additional high resolution laser-ARPES studies (for example Ref 10, [Vishik, I. M. et al. PNAS 109, 18332–18337 (2012)]) make the point that the near-nodal gapsize (for samples with a true node in the gap function) is very consistent across doping.

Version 2:

Reviewer comments:

Reviewer #1

(Remarks to the Author)
I have re-read the revised manuscript, the review reports, and the authors’ rebuttal. Overall, I find that the authors have made substantive revisions addressing the major concerns raised in previous rounds. My assessment is as follows. In my opinion, the experimental findings remain important and merit publication in Nature Communications.
1. A central concern raised by all reviewers was the lack of observable band splitting after surface doping. In the revised version, the authors appropriately acknowledge that their explanation is speculative, and they have also removed the overly strong claim that the IP doping change is below 1%. I consider this an improvement. Although the microscopic mechanism of the surface doping effect is still not fully clarified, the key experimental observations that the IP remains highly underdoped while the OP becomes substantially more doped, appear sufficiently supported and continue to underpin the main conclusions of the manuscript.
2. The comparison with Bi2223 (Fig. R2) is useful in supporting the claim that the IP is highly underdoped. To further strengthen the interpretation, I suggest that the authors also compare the OP Fermi surface after surface doping with the OP Fermi surface of Bi2223 in the same plot (Fig. R2). Such a comparison could provide additional support for the scenario in F0223 that a highly overdoped metallic OP layer screens the highly underdoped IP layer and helps enhance superconducting coherence.

Reviewer #2

(Remarks to the Author)
The authors have tried to address the remaining issues without providing additional experimental evidence. I disagree with the authors' point that the absence of OP band splitting is not essential to the central conclusion of this work. The absence of OP band splitting is directly linked to the central question that whether the doping level of each layer is correctly estimated. I have to point out that the correct estimation of the doping level is the basis for the key conclusion of this paper.

In principle, systematic photon energy dependent measurements would clearly demonstrate the authors' first speculation. If the band splitting is observed, then all the experimental observations would become more reasonable. Nevertheless, if the authors decide not to provide additional experimental evidence, then it is important to state the speculations and clarify the possible uncertainties related to the key conclusion of this paper.

Reviewer #3

(Remarks to the Author)
I appreciate the authors response and think the decision to remove the claim that the doping level difference between the pristine and surface-doped IP is less than 1% is a correct one.
I also appreciate the direct comparison to the IP of Bi2223, which makes it clear that the surface-doped F0223 IP is much more coherent outside of the AFZB, despite having a significantly lower doping level.

At the same time, it is important to recognize that these are two different material systems, potentially with different underlying phenomenologies. Therefore, it is not strictly guaranteed that the two IPs should behave identically as a function of doping. While the comparison provides a useful indication supporting the authors’ interpretation, this limitation should be explicitly acknowledged. In particular, it remains possible that dopants contribute in some way to the enhanced coherence of the F0223 IP.

For this reason, I recommend softening the language in the newly added section and incorporating a more balanced discussion reflecting this caveat. The data remain novel and interesting, and I am in favour of publication provided the discussion is adjusted accordingly.

Version 3:

Reviewer comments:

Reviewer #1

(Remarks to the Author)
I have reviewed the revised manuscript. The authors have softened the language regarding the absence of observable band splitting after surface doping and have added a useful comparison between the OP Fermi surfaces of surface-doped F0223 and overdoped Bi2223. The main experimental observations remain interesting and important. I therefore support publication of the manuscript in Nature Communications.

Reviewer #2

(Remarks to the Author)
The authors have done a good job in the response. I recommend the publication of this work.

Reviewer #3

(Remarks to the Author)
The authors have satisfactorily addressed my comments in the revised manuscript, and with the softened language I recommend publication.

Open Access This Peer Review File is licensed under a Creative Commons Attribution 4.0 International License, which permits use, sharing, adaptation, distribution and reproduction in any medium or format, as long as you give appropriate credit to the original author(s) and the source, provide a link to the Creative Commons license, and indicate if changes were made.
In cases where reviewers are anonymous, credit should be given to 'Anonymous Referee' and the source.
The images or other third party material in this Peer Review File are included in the article's Creative Commons license, unless indicated otherwise in a credit line to the material. If material is not included in the article's Creative Commons license and your intended use is not permitted by statutory regulation or exceeds the permitted use, you will need to obtain permission directly from the copyright holder.
To view a copy of this license, visit https://creativecommons.org/licenses/by/4.0/

Reviewer #1 (Remarks to the Author):

Summary of Reviewer #1

In this manuscript, Jeong et al. investigate the electronic structure of the five-layer $(Cu,C)Ba_2Ca_4Cu_5O_y$ (Cu1245) and tri-layer $Ba_2Ca_2Cu_3O_6(F, O)_2$ (F0223) cuprates—systems that have not been studied by ARPES previously. They find that, in both Cu1245 and surface-doped F0223, the inner CuO_2 plane (IP) is superconducting, whereas the outer planes (OPs) remain non-superconducting metals. In surface-doped F0223, the IP exhibits the largest superconducting gap ($\Delta \approx 60$ meV) together with a coherent Bogoliubov quasiparticle peak near the antinode. Remarkably, the IP doping level is below ~1%. The authors argue that interlayer coupling—and therefore the superconducting proximity effect—is weak, and that the highly doped OPs screen disorder from the dopant layers, rendering the IP an effectively disorder-free superconducting CuO_2 sheet. These interesting results refresh the field and provide a unique platform for probing high-T_c mechanisms in cuprates. On this basis, I believe the work meets the high standards of Nature Communications and recommend publication after minor revision. I outline a few minor comments below that could further strengthen the manuscript.

We thank the reviewer for the concise and accurate summary of our work and for the positive assessment. We are pleased that the reviewer recognizes the significance of our results. In the revised manuscript, we have carefully addressed all comments and suggestions. Below, we provide our detailed point-by-point responses.

Comment 1.1

1. It is not clear why, in F0223, the OPs can be doped to ~30% while of the IP remains below ~1%, in contrast to the trilayer cuprate Bi2223 with a similar structure. The authors argue that the large difference in the e_l reduces the interlayer hopping and enhances the screening. Why does the F0223 have a larger difference in e_l compared with Bi2223? Is F0223 a special case or other trilayer system, like Bi- and Hg-, that may have a similar effect when doping enough hole carriers?

We appreciate the reviewer's question. F0223 has a unique crystal structure that differs from that of Bi2223, leading to a large potential difference.

In Bi-based systems such as Bi2223, the Bi-O layers, which serve as charge-reservoir (dopant) layers, are located above and below the apical oxygen sites of the outer CuO_2 planes (OPs). In contrast, in F0223, the apical oxygen/fluorine layer itself acts as the dopant layer. As a result, the OPs in F0223 are more effectively carrier-doped from the dopant layer than those in Bi2223.

Furthermore, the carrier concentration of each CuO_2 plane increases as its distance from the dopant layer decreases. Therefore, the OPs are generally more heavily doped than the inner planes (IPs). In F0223, this doping imbalance between the OPs and IPs is further enhanced because the OPs are directly bonded to the apical oxygen/fluorine dopant layer. This unique structural configuration naturally explains why F0223 exhibits a larger potential difference between the OP and IP than Bi2223.

That said, we expect that the physics presented in our manuscript is general to all multilayer cuprates, including Bi- and Hg-based compounds. In general, when the OPs—regardless of the compound—are more heavily doped and thus more metallic, their higher carrier mobility results in stronger screening of disorder in the inner planes (IPs). However, in most other cuprates, it is difficult to selectively dope only the outer layers in large amounts without noticeably changing the doping level of the inner layers. In this sense, while the mechanism we discuss is universal to multilayer cuprates, F0223 provides an exceptionally clear manifestation of intrinsic cuprate physics owing to its unique crystal structure.

We added this discussion to the revised version of our Supplementary information.

Comment 1.2

2. Upon surface dosing, is only the top OP doped, or is the OP below the IP also doped sufficiently to enable effective screening? Did the author observe band splitting of the OPs after surface doping?

We appreciate the reviewer's question. From our data, we cannot unambiguously determine whether only the top OP is doped or the OP beneath the IP is also doped. Experimentally, no band splitting between the two OPs was observed.

One might expect that surface doping induces a larger hole concentration in the top OP layer than in the bottom OP layer, and thus that the two OP bands—with different hole concentrations—would appear as distinct split features in ARPES. However, our data show no such splitting. We propose two possible explanations for these observations:

(1) ARPES is a surface-sensitive technique characterized by a short photoelectron escape depth. The bottom OP may simply be too deep to emit photoelectrons into the vacuum. This is plausible because the distance from the cleavage plane to the bottom OP is approximately 10.1 Å [Ref. R1], whereas the probing depth of 55 eV photons is extremely short (~4.4 Å) [Ref. R2]. We note, however, that the actual probing depth can vary depending on the material and the chemical composition, so this estimate should be taken as a rough guide.

(2) It is also possible that oxygen atoms diffuse not only into the top dopant layer but also into the bottom dopant layer beneath the bottom OP. This would lead to overdoping in both the bottom and top OPs. In this case, the electronic bands from the top and bottom OP layers would overlap, consistent with the absence of band splitting in the ARPES data.

We emphasize that, in either scenario, our conclusion of enhanced coherence driven by outer-layer metallic screening remains unchanged and is strongly supported by our data.

We added this discussion to the revised version of our Supplementary Information.

Comment 1.3

3. Can the author quantify the newly introduced "the degree of screening" or connect it to other quantities?

We have not yet established a direct way to quantify "the degree of screening." However, it can be connected to other physical quantities.

One possible quantity is the width of the energy-gap distribution obtained from the gap-map histogram in STM measurements, which is known to be sensitive to disorder. If an inner layer is effectively screened from the random potential induced by the dopant layer, the electronic states in that layer should become more homogeneous. In such a clean layer, the inhomogeneity of the gap state in the local density of states (LDOS) would likely be suppressed or even eliminated. Specifically, the histogram of the gap map would become sharper. The width of this gap distribution, therefore, could serve as a measure reflecting "the degree of screening."

Another relevant quantity is the width of the quasiparticle peak observed by ARPES, which is proportional to the electron scattering rate and thus should be sensitive to disorder. As a more homogeneous state is realized, the scattering rate decreases, leading to a sharper quasiparticle peak. Therefore, the peak width (or scattering rate) provides another possible quantitative indicator of the "degree of screening."

It should be noted, however, that STM mainly probes the topmost OP layer, so it is technically challenging to access the screened inner planes. Similarly, surface contamination may broaden the intrinsic spectral linewidth of the clean IP due to additional electron scattering during the photoemission process. Therefore, ARPES and STM are not ideal for directly quantifying "the degree of screening," and developing a reliable and direct experimental method to extract this quantity remains an important challenge for future studies.

We added this discussion to the revised version of our manuscript.

Comment 1.4

4. The sample temperature during surface doping is not specified in the manuscript.

We apologize for this ambiguity in our manuscript. The sample temperature was maintained at the lowest temperature (~7 K) throughout the surface-doping procedure and all subsequent measurements.

We note that the previous study by Palczewski et al. [Ref. R3] demonstrated that surface doping toward the overdoped regime occurs at low temperatures, whereas the opposite doping tendency toward underdoping appears when the sample is kept at higher temperatures (such as room temperature) due to annealing effects in vacuum, which remove oxygen from the sample surface.

This information has been explicitly added to the revised version of our manuscript.

Reviewer #2 (Remarks to the Author):

Summary of Reviewer #2

The manuscript reports ARPES measurements and theoretical calculations on multilayer-cuprates. In five-layer cuprate Cu1245, the superconducting gap is observed on the IP band, but not on the OP band. In three-layer cuprate F0223, the superconducting gap is identified on both IP and OP bands of the pristine sample. However, after exposing the cleaved surface of the sample to a small air leak in the vacuum chamber, the energy gap disappears on the OP band, but persists on the IP band. A possible surface doping is suggested and a screening effect is discussed. In addition, a coherent flat band is observed near the Brillouin zone edge of the IP. Based on these observations, the manuscript further suggests the existence of interlayer decoupling and clean single CuO_2 layers, on which a simultaneous realization of strong pairing and strong phase stiffness can be realized. I would like to mention at the outset that it is indeed important to investigate multilayer-cuprates. I also agree that multilayer-cuprates provide a special platform to look for the protected clean CuO_2 planes and examine the individual roles of interlayer coupling and the presence of clean CuO_2 planes, respectively. In this context, the current study may provide important information to the community of high T_c superconductivity. With that being said, I do have serious concerns on both the experiments and interpretations. Therefore, I cannot recommend the publication of this manuscript, at least, not the current version.

We appreciate the referee's thoughtful summary of our results and the recognition of the importance of studying multilayer cuprates. We fully agree that the multilayer system and the disorder-screened, clean inner CuO_2 planes provide key platforms for exploring the nature of high-T_c superconductivity. Below, we provide detailed point-by-point responses to all the comments raised by the referee. We have also revised our manuscript accordingly to address these points.

Comment 2.1

First, the so-called surface doping in the current study is achieved by exposing the cleaved surface to a small air leak (~3 x 10^{-9} Torr) in the vacuum chamber. Typically, the exposure of freshly cleaved sample surface to an air leak would destroy the surface, instead of inducing a simple doping effect. As the authors mentioned, in-situ ozone annealing is typically used to induce hole doping in cuprates. The local oxygen pressure provided by ozone annealing is significantly different from that of a small air leak. In fact, when an overdoped cuprate (*e.g.* overdoped Bi2212 with T_c~50 K) is cleaved in vacuum, the surface layer starts to lose oxygen. Therefore, it is hard to imagine that the exposure of freshly cleaved surface to a small air leak (with the vacuum of ~3 x 10-9 Torr) can induce oxygen to the sample and even dope the sample to the extremely overdoped non-superconducting region. In this sense, it is important to experimentally demonstrate that the absence of an energy gap on the 'surface-doped' OP band is not due to sample aging effect or a degraded sample surface.

We acknowledge the reviewer's concern regarding the possible sample aging effect. Indeed, ozone annealing is a well-established method for increasing the hole carrier concentration in cuprates. However, it has been demonstrated that exposing a cleaved surface to a small air leak at low temperatures can also significantly increase the hole concentration, as reported by Palczewski et al. [Ref. R3, also cited in our manuscript as Ref. 38]. They also showed that the hole concentration decreases due to oxygen loss from the surface when the cleaved surface is kept in a vacuum, as correctly pointed out by the reviewer. We emphasize, however, that this reduction occurs at high temperatures, which are different from our experimental conditions.

Importantly, the evolution of the superconducting gap under such surface treatments shows no evidence of severe surface degradation that could destroy superconductivity.

Nevertheless, we acknowledge that the air leak may still cause some degree of surface degradation. To address this, we demonstrate below in detail that well-defined quasiparticle peaks remain observable in the surface-doped OP, indicating that the OP is not severely damaged to a level that would suppress superconductivity.

Furthermore, we quantitatively evaluate the extent to which the "surface-doped" OP was degraded compared with the "pristine" OP by comparing their spectral peak widths, which correspond to the inverse of the quasiparticle lifetime. We find that the peak of the surface-doped OP is only about 45% broader than that of the pristine OP, and the linewidth remains within the range reported for cuprates in the literature. Therefore, it is unlikely that our surface was severely damaged enough to eliminate superconductivity.

Demonstration of well-defined quasiparticles with a sharp spectral peak:

The difficulty in evaluating the spectral peak width in multilayered cuprates arises from the fact that each spectrum is a superposition of multiple states. However, it is possible to extract the intrinsic spectral contribution of each state by fitting a model function, as described below.

The spectral intensity detected by ARPES, $I(k, \omega)$, is proportional to the spectral function $A(k, \omega)$ multiplied by the Fermi-Dirac distribution function $f_{\mathrm{Fermi}}(\omega)$ at measurement temperature, which is then convolved by a Gaussian $f_{\mathrm{Gauss}}(\omega)$ whose width corresponds to the experimental energy resolution. In particular, it is expressed as follows, for the case of three-layer cuprates as F0223, which consists of inner planes (IPs) and outer planes (OPs):

$$I(k,\omega) = [A_{\mathrm{coh}}^{\mathrm{IP}}(k,\omega) + A_{\mathrm{coh}}^{\mathrm{OP}}(k,\omega) + A_{\mathrm{inc}}(k,\omega)] \cdot f_{\mathrm{Fermi}}(\omega) \circledast f_{\mathrm{Gauss}}(\omega)$$

Here, $A_{\mathrm{coh}}^{\mathrm{IP}}$ and $A_{\mathrm{coh}}^{\mathrm{OP}}$ are coherent parts for IP and OP, respectively, and modeled as Lorentzians. A_{inc} represents the overall incoherent contribution from both IP and OP, which is modeled as a broad Lorentzian multiplied by an asymmetric term, following Ref. R4.

Figures R1d and e overlay the EDCs at the nodal k_F point and their corresponding fitting curves for the pristine OP and the surface-doped OP, respectively. We find a background intensity

coming from another band, which is not taken into account in the above equation, at high binding energy. In particular, the background intensity in the surface-doped sample is relatively large. Since this additional band is non-dispersive, the spectrum off the k_F point can be reliably assumed to be the background intensity. This is demonstrated in Figs. R1a and R1c by overlaying the spectra at k_F and off-k_F, taken along the blue and magenta dotted lines in Fig. R1a. The intensity difference between these two spectra is plotted in Fig. R1e, which is used for fitting to the above function.

Figures R1d, e show the fitting results for pristine and surface-doped samples, respectively. The current focus is the spectral peak of OP, $A_{\mathrm{coh}}^{\mathrm{OP}}(k_{\mathrm{F}}, \omega)$, in particular, its energy width ($\Delta\varepsilon$), which is proportional to the scattering rate (or inverse of quasiparticle lifetime: $\Delta\varepsilon/\hbar$=1/τ). Importantly, we find a sharp OP peak not only in the pristine sample but also in the surface-doped sample, indicating that the latter's surface is not severely damaged to the extent that superconductivity would be destroyed.

By the fitting, we obtained $\Delta\varepsilon$ as ~37 meV and ~49 meV for the pristine and surface-doped samples, respectively (Fig. R1f). To confirm the relevance of this value extracted from EDC analysis, we next conducted MDC (momentum distribution curve) analysis, which is commonly used to estimate physical parameters due to its simple Lorentzian shape, enabling easy fitting.

$\Delta\varepsilon=\hbar/\tau= \hbar\Delta k_F \cdot v_{\mathrm{F}}$ can be expressed as the product of the MDC peak width at the Fermi level (Δk_F) and the Fermi velocity (v_{F}), which is determined from the MDC peak dispersion. Estimation of Δk_F and v_{F}, especially for the surface-doped OP, is demonstrated in Fig. R1a and b, respectively. The Δε estimated via the MDC analysis is ~55 meV; this is consistent with the one obtained by the EDC analysis (~49 meV) within the analysis error.

From this scattering rate analysis with both EDC and MDC, we acknowledge that the surface-doped OP shows a slight degradation, as characterized by approximately 45% increase in the spectral width compared to the pristine OP. However, a nodal $\Delta\varepsilon$ of ~50 meV falls within the typical range reported in previous ARPES studies of cuprates [Refs. R5−R7], confirming that the quasiparticles in the surface-doped OP remain well-defined. We therefore conclude that the observed gap closure in OP by surface doping that transformed the OP into a heavily overdoped metallic state originates from heavy-carrier doping rather than surface degradation.

We have added these discussions and Fig. R1 to the revised version of our Supplementary Information.

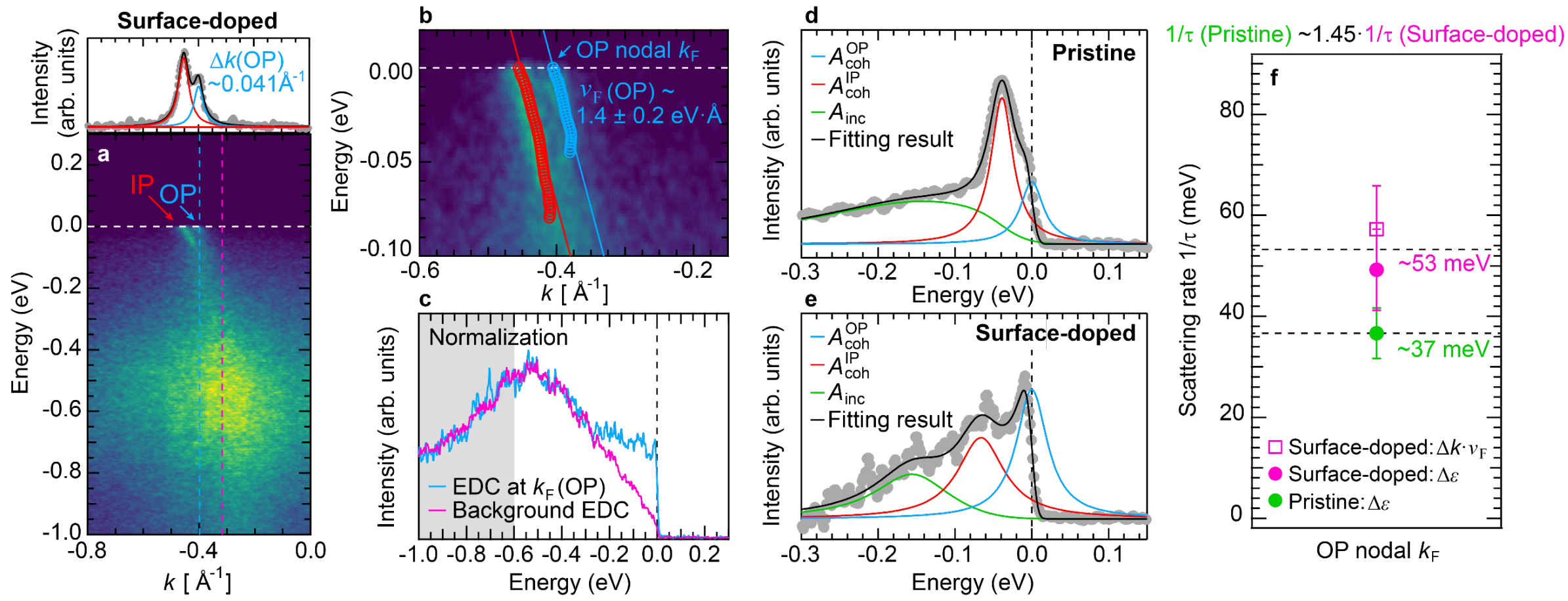

Fig. R1. **a,** Energy-momentum dispersion of surface-doped F0223, taken along the nodal direction. The upper panel shows a momentum distribution curve (MDC) at E_F. **b,** Enlarged plot of (**a**). Band dispersions are obtained by peak positions of MDCs by Lorentzian fitting. The Fermi velocity v_F is obtained by linearly fitting the band dispersions near E_F. **c,** EDC at k_F of the surface-doped OP (blue) and background curve (magenta). Each curves are taken from the dotted line in (**a**), then normalized by the area integrated by an energy range of [-1, -0.6] eV (grey shaded area). **d,** EDC fitting result for the pristine OP at nodal k_F point. **e,** Similar to (**d**), but for the surface-doped OP. The EDC is shown after subtracting the background shown in (**c**). **f,** Extracted scattering rates for the pristine and surface-doped OP, by EDC and MDC analysis.

Comment 2.2

Second, the manuscript suggests that the surface OP layer is hole-doped by the oxygen and the IP layer remains nearly unchanged. Following this assumption, one would believe that the OP layer underneath the IP layer remains the same as that in the pristine sample. Therefore, the doping levels of the two OP layers would become significantly different. Thus, one should observe two sets of bands and Fermi surfaces from the OP layers, which is not the case in the current study. It is also hard to reconcile such a problem by assuming that the electronic structure of the other OP layer (underneath the IP layer) cannot be probed by ARPES. Because earlier ARPES results have demonstrated that not only the bands but also the interlayer coupling between the two OP layers can be probed by ARPES in three-layer cuprates.

We appreciate the reviewer's insightful remark. As pointed out, one might expect that surface doping induces a larger hole concentration in the top OP layer than in the bottom OP layer, and thus that the two OP bands—with different hole concentrations—would appear as distinct split features in ARPES. However, our data show no such splitting. We propose two possible explanations for these observations:

(1) ARPES is a surface-sensitive technique characterized by a short photoelectron escape depth. The bottom OP may simply be too deep to emit photoelectrons into the vacuum. This is plausible because the distance from the cleavage plane to the bottom OP is

approximately 10.1 Å [Ref. R1], whereas the probing depth of 55 eV photons is extremely short (~4.4 Å) [Ref. R2]. We note, however, that the actual probing depth can vary depending on the material and the chemical composition, so this estimate should be taken as a rough guide.

(2) It is also possible that oxygen atoms diffuse not only into the top dopant layer but also into the bottom dopant layer beneath the bottom OP. This would lead to overdoping in both the bottom and top OPs. In this case, the electronic bands from the top and bottom OP layers would overlap, consistent with the absence of band splitting in the ARPES data.

We emphasize that, in either scenario, our conclusion of enhanced coherence driven by outer-layer metallic screening remains unchanged and is strongly supported by our data.

As pointed out by the reviewer, the earlier ARPES study reported an interlayer band splitting of OPs in the trilayer stack [Ref. R8]. However, our data does not show such splitting, not only in the surface-doped samples but also in the pristine samples. We also note that a similar splitting has not been observed in another trilayer cuprate, $HgBa_2Ca_2Cu_3O_{8+\delta}$ (Hg1223) [Ref. R9, also cited in our manuscript as Ref. 43]. These results suggest that the coupling strength between OPs varies among different cuprate compounds. The coupling strength may also be affected by the carrier concentration of the OPs, as it is expected to be enhanced in overdoped states. This is indeed an interesting topic, but it lies beyond the scope of the present study.

We added these discussions to the revised version of our Supplementary Information.

Comment 2.3

Third, the current work suggests that both a large superconducting gap and strong phase stiffness are simultaneously realized in the single CuO_2 sheet, pointing to an ideal platform for realizing high T_c superconductivity. One can understand that the superconducting T_c of the so-called surface doped sample cannot be easily determined by transport and magnetic measurements. However, it is straightforward to show the temperature evolution of the superconducting gap probed by ARPES. Does the superconducting gap of the surface doped sample persist to a temperature higher than that of the pristine sample?

The superconducting transition temperature (T_c) of the surface-doped sample is indeed an interesting subject. However, determining T_c from ARPES measurements is not feasible for the following reasons.

Previous ARPES studies have revealed that the *d*-wave superconducting gap with a single node persists well above T_c [Ref. R10]. Therefore, the temperature at which the gap closes is not an appropriate indicator of T_c, particularly in the underdoped state, as in the IPs.

We also note that temperature-dependent measurements are experimentally impractical under the present setup. In the small air-leak environment, residual gases accumulate over a wide area of the manipulator that is cooled together with the sample. Even a modest temperature increase induces substantial outgassing, leading to surface degradation.

We appreciate the reviewer's understanding that such a specialized setup inherently entails both advantages and limitations for the purpose of our study. Nevertheless, our data convincingly demonstrate that quasiparticle coherence is significantly enhanced by metallic screening from the outer CuO_2 layer, providing an important new insight into the physics of layered perovskite cuprates.

Comment 2.4

Fourth, the sharp EDC peaks in the surface doped IP bands are indeed surprising. However, one can clearly see a step like drop at E_F (*e.g.* several EDCs in Fig. 4k). In this case, is it still valid to claim an energy gap by the peak of the EDC?

We thank the reviewer for pointing this out. The "step-like drop" or residual spectral weight near E_F is observed not only at the k_F point but also at momenta away from k_F, and is therefore attributed to photoemission background intensity rather than intrinsic spectral features.

In Fig. R2a-e, we plot EDCs at several k_F points together with a background extracted from momenta slightly away from k_F (see also Fig. R1a). Even without intensity normalization, the background curve aligns well with the high-binding-energy tail of the EDC at k_F and exhibits a similar "step-like drop" near E_F. After background subtraction, the residual spectral weight near E_F is significantly reduced (Fig. R2f-j).

Given that the remaining spectral weight at E_F after background subtraction is very small compared with the coherence-peak intensity, we conclude that the primary superconducting properties are governed by these coherence peaks. Accordingly, defining the superconducting energy gap by the position of the coherence-peak maximum is physically justified.

The "step-like drop" or residual spectral weight near E_F, which is observed over a wide momentum range (or angle range), may arise from inelastic scattering of mobile carriers in a metallic band (*e.g.*, from the outer plane) during the photoemission process. However, identifying its microscopic origin lies beyond the scope of the present study.

We added this discussion and Fig.R2 to the revised version of our Supplementary Information.

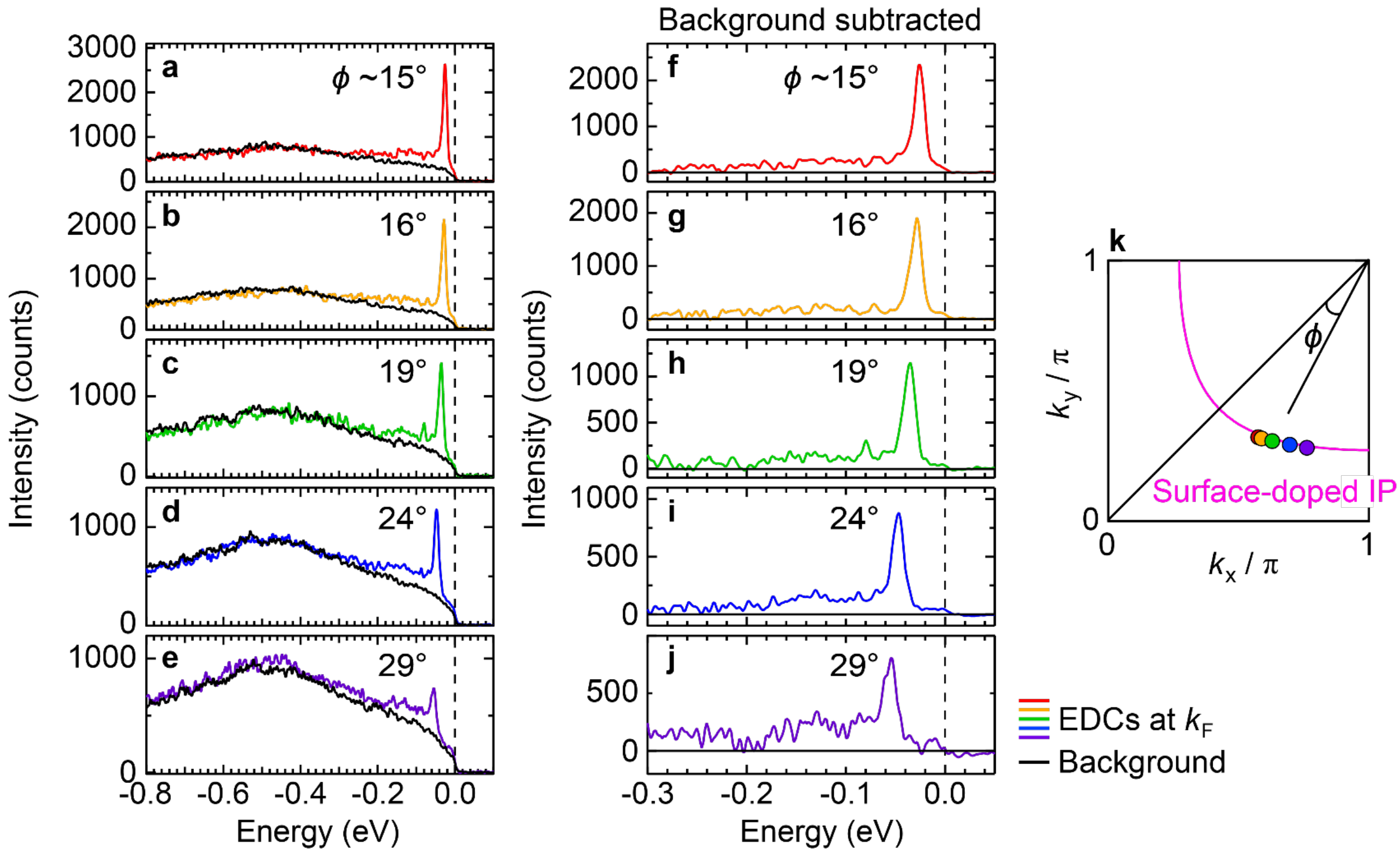

Fig. R2 a-e, EDCs taken from various k_F points in the surface-doped IP, as indicated by colored circles in (**k**). These EDCs are the same curve shown in Fig. 4k. Black curves are backgrounds taken from the momenta slightly off from k_F. **f-g,** The same curves as in (**a-e**), but shown after the background subtraction. **k,** Schematic Fermi surface of surface-doped IP and the k_F points where the EDCs in (a-j) were taken.

Comment 2.5

Fifth, the observation of a flat-band-like feature is interesting. However, the understanding of its origin is important. In particular, possible extrinsic effects induced by degraded surface should be ruled out experimentally.

We agree that it is important to consider the possibility that the flat band might be induced by surface degradation and is therefore unrelated to the intrinsic electronic properties of cuprates. Here, we rule out this possibility by demonstrating that the flat band is already present, albeit with weak intensity, in the pristine samples. In the surface-doped samples, this same flat band becomes significantly more coherent, as evidenced by the sharpening and enhanced intensity of its spectral peak.

In Fig. R3, we show a comparison between the pristine and surface-doped samples. Each spectrum was measured along the Brillouin zone boundary. Although the intensity is very weak in the pristine sample, a flat-band-like feature similar to that of the surface-doped IP can be identified at the same binding energy (Figs. R3a-d). This feature becomes more evident in the curvature plots (Figs. R3e, f), which enhance the visibility of spectral peaks. Notably, the peak

position remains unchanged upon surface doping, while the coherence, reflected by a higher peak intensity and a narrower linewidth, is greatly enhanced. We therefore conclude that the coherent flat band near (π, 0) in the surface-doped IP is not an extrinsic effect but an intrinsic feature of cuprates, which becomes particularly pronounced when disorder effects in the CuO_2 layers are suppressed.

One possible origin of the flat band is that it arises from the combined effects of a large superconducting gap and collective mode coupling, both of which can flatten the band dispersion near the Fermi level. However, a more detailed theoretical analysis will be required to fully understand its underlying physics.

We have added these discussions and Fig. R3 to the revised version of our manuscript and Supplementary Information.

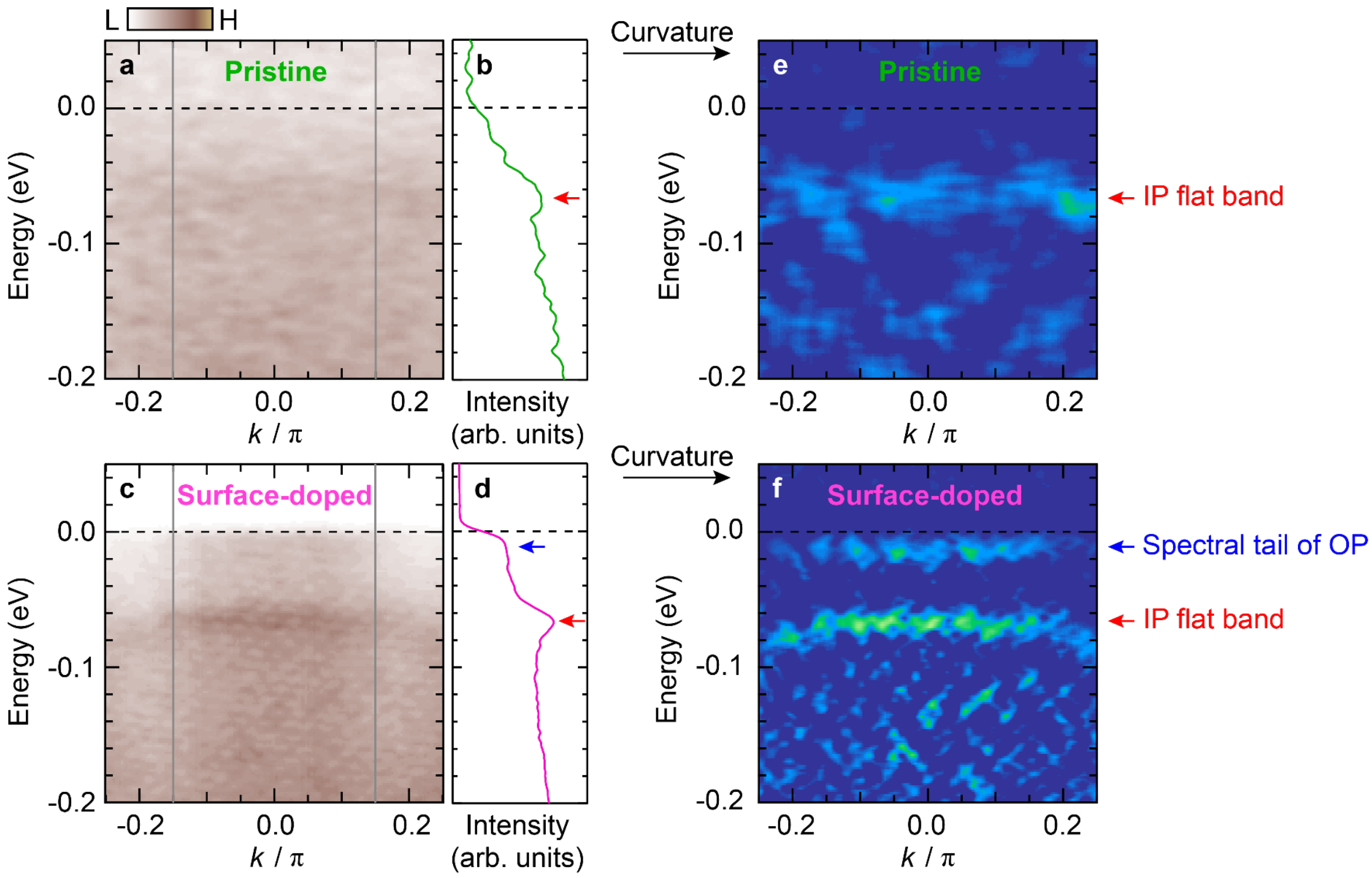

Fig. R3 a, Energy-momentum dispersion of Surface-doped F0223, taken at the zone boundary. **b,** Integrated EDC of (**a**), within the energy range of [-0.15, 0.15] eV (grey solid line in (**a**)). **c, d,** Similar data to (**a, b**), but for the surface-doped F0223. The data shown in (**a-d**) is the same data shown in Fig. 5f, g, k. **e, f,** The curvature plot of (**a, c**), showing the existence of a flat-band (red arrows).

Comment 2.6

Sixth, the ARPES results on the five-layer Cu1245 seem to be valid. However, the bands from the IP_1 and IP_0 are much weaker than that from the OP. It has been reported that the relative intensity of the bands from IP and OP can be changed by different photon energies and polarizations. More systematic measurements would enhance the data quality for the

quantitative gap analysis.

We appreciate the reviewer's suggestion. In our current measurements, the weaker signal from the IPs compared with the OPs is quite natural, considering that the OP is located closer to the cleaved surface and therefore is more efficiently photoexcited. Nevertheless, the spectral intensities of IPs are still strong enough to reach our conclusion reliably.

We agree that the reviewer's proposed experiments would be valuable for optimizing the relative photoemission intensities of the IP and OP. We hope that such detailed studies will be conducted in future follow-up studies.

Reviewer #3 (Remarks to the Author):

Summary of Reviewer #3

I read with interest the manuscript "Superconducting coherence boosted by outer-layer metallic screening in multilayered cuprates" by Jeong et al, where the authors present detailed ARPES measurements on two multilayered cuprates: the five-layered system Cu1245 and the triple-layered F0223. Both compounds exhibit a novel and quite interesting electronic structure where only the bands assigned to the Inner Planes (IP) show signatures of superconductivity, and those belonging to the Outer Planes (OP) do not. The authors claim that in these systems, contrasting the more familiar and well studied multilayered systems such as Bi2223, there is no hybridization between the distinct FS in the superconducting state. Instead, they propose that the main role of the OP is to screen the underdoped IP from disorder, thereby boosting its superconducting coherence. I think the data-quality is excellent, the manuscript is well written and the story is compelling. However I do have some concerns regarding the interpretation of the presented data that would need to be sufficiently addressed before recommending publication.

We thank the reviewer for the positive evaluation of our work and for the clear summary of our results. Below, we provide detailed point-by-point responses to all comments and have revised our manuscript accordingly. We hope that the reviewer will find the revised manuscript suitable for publication.

Comment 3.1

My main concern lies with the claim of the authors that in the three-layered compound F0223, after exposure of the sample surface to air, the OPs are doped to such a degree they are no longer superconducting, while crucially, the IPs remain at the same doping and only gain SC coherence due to the metallic nature of the now overdoped OPs. The authors say that the IP doping is unchanged, based on the near-nodal gap size and Fermi momenta (as the authors are not able to identify an IP dispersion outside of the AFZB before the air exposure). I do not find this reasoning particularly convincing. While it is clear that the OP nodal k_F has changed, It seems entirely possible, and perhaps even likely for the IP doping to also be different, with this being mainly expressed by a modified gap and dispersion far away from the zone diagonal,

similar to what is observed when doping Bi2201 [1](ref. (40)) and 6-layer BaCCO(F, O) [2] (ref. (7)). My issue here is that even a small increase in doping of the IP could very well lead to an increased superfluid density and a growth of the coherence peaks.

We thank the reviewer for the constructive comment. We agree that it is unlikely that carrier doping into the IP due to surface doping is strictly zero. However, we estimate that the variation is very small, within approximately 1%, based on tight-binding fits to the Fermi surfaces. The Fermi surface near (π, 0) cannot be precisely determined from our data. Nevertheless, the Fermi-surface shape near (π, 0) is relatively insensitive to doping variations in the IP, which is heavily underdoped.

The data of Ref. 40 for Bi2201 correspond to doping levels ranging from heavily overdoped to lightly underdoped, where the van Hove singularity lies relatively close to the Fermi level. In that case, the Fermi-surface shape near (π, 0) is highly sensitive to small shifts of the Fermi energy associated with doping variations. This contrasts with the present case of IPs in a heavily underdoped regime, where the van Hove singularity lies deeply below the Fermi level; thus, the Fermi-surface shape near (π, 0) is relatively insensitive to doping variations, even compared to the nodal direction. Therefore, the data excluding the (π, 0) region are sufficient for an accurate comparison of the Fermi-surface volume.

In addition, our current data do not show a small Fermi pocket but rather a conventional large Fermi surface, although it exhibits an arc-like feature. Thus, our data cannot be directly compared with that of the six-layer $Ba_2Ca_5Cu_6O_{12}(F,O)_2$ [Ref. R11], which exhibits small Fermi pockets.

Our conclusion of a very small doping change in IPs by surface doping is further supported by the observation that the superconducting gap near the node remains unchanged. Previous ARPES studies on $Bi_2Sr_2CaCu_2O_{8+\delta}$ (Bi2212) have revealed that the superconducting gap near the node varies significantly with doping in the underdoped region [Refs. R5, R12]. Both single-crystal studies [Ref. R5] and surface treatments via ozone annealing [Ref. R12] similarly show that the antinodal superconducting gap (Δ_0), obtained by extrapolating the near-nodal gap to the antinode, approximately follows the superconducting dome with doping. Therefore, the nearly identical Δ_0 values for the pristine and surface-doped IPs in our F0223 sample (main Fig. 4m) indicate that the doping difference must be very small.

We added this discussion to the revised version of our Supplementary Information.

Comment 3.2

Related to this, the quality of the OP band clearly degrades after the surface doping, presumably due to disorder. If the claim is that the IP is completely protected from this disorder due to the above lying OP, one might expect the “second” OP (the one on the bottom of the surface trilayer stack) to also not be affected by this surface disorder. However, there is no sign of a splitting of OP bands after the surface dosing.

We appreciate the reviewer's insightful remark. As pointed out, one might expect that surface doping induces a larger hole concentration in the top OP layer than in the bottom OP layer, and thus that the two OP bands—with different hole concentrations—would appear as distinct split features in ARPES. However, our data show no such splitting. We propose two possible explanations for these observations:

(1) ARPES is a surface-sensitive technique characterized by a short photoelectron escape depth. The bottom OP may simply be too deep to emit photoelectrons into the vacuum. This is plausible because the distance from the cleavage plane to the bottom OP is approximately 10.1 Å [Ref. R1], whereas the probing depth of 55 eV photons is extremely short (~4.4 Å) [Ref. R2]. We note, however, that the actual probing depth can vary depending on the material and the chemical composition, so this estimate should be taken as a rough guide.

(2) It is also possible that oxygen atoms diffuse not only into the top dopant layer but also into the bottom dopant layer beneath the bottom OP. This would lead to overdoping in both the bottom and top OPs. In this case, the electronic bands from the top and bottom OP layers would overlap, consistent with the absence of band splitting in the ARPES data.

We emphasize that, in either scenario, our conclusion of enhanced coherence driven by outer-layer metallic screening remains unchanged and is strongly supported by our data.

We added this discussion to the revised version of our Supplementary Information.

Comment 3.3

Some additional points:
Later on, the authors speculate that a decreasing gap on the OP but increasing IP coherence might raise T_c, however without any experimental evidence. This would be an opposite scenario to what is suggested in [3] (ref. (43)), where it is actually the surprisingly large gap on the OPs of Hg1223 that is causing an increased T_c compared to for example Bi2223.
In general I feel a more comprehensive discussion is warranted about the differences between the compounds presented here and the more commonly studied multilayers Bi2223 and Hg1223, both in terms of crystal and electronic structure because the intrinsic physics seems to a degree different [3,4] (refs (16,43)) as well as recent arXiv papers such as [5] and [6]).

We agree that it is important to consider the relationship among different trilayer cuprates. The theoretical framework proposed by Kivelson et al. [Ref. R13] suggests that trilayer systems are favorable for achieving high T_c because the weak phase stiffness of underdoped layers and the weak pairing strength of overdoped layers can compensate each other through interlayer coupling. Such coupling has been experimentally observed [Refs. R14, R15] in the form of Bogoliubov hybridization, where band bending in the superconducting state causes a crossing between the OP and IP bands, leading to a mixing of their wavefunctions.

The results of Horio et al. [Ref. R9, also cited in our manuscript as Ref. 43] indicate that the OP in Hg1223 is less disordered than that in Bi2223, resulting in an enhanced superconducting

gap. It is therefore natural that T_c is higher in Hg1223 than in Bi2223. In general, the realization of both large phase stiffness and strong pairing strength through interlayer coupling is a well-accepted framework for achieving high T_c in trilayer cuprates.

In our surface-doped samples, however, the OP is in a normal metallic state. Consequently, no Bogoliubov back-bending occurs in the OP band, and the IP cannot hybridize with the OP. As a result, the IP becomes electronically isolated from interlayer coupling with the OPs. Nevertheless, our results demonstrate that even an isolated single IP can satisfy the favorable conditions—namely, strong phase stiffness and large pairing strength—without the need for interlayer coupling.

Notably, $HgBa_2CuO_{4+\delta}$ (Hg1201), known as a clean single-layer cuprate with minimal disorder, achieves a T_c of about 100 K. Importantly, the IP in our surface-doped samples can be even cleaner. This is supported by the nuclear magnetic resonance (NMR) measurements on Hg1201 [Ref. R16] and F0223 [Ref. R17], showing that the NMR spectral peak of IP for F0223 is sharper than that of Hg1201. This indicates that the pristine IP of F0223 is less disordered than that of Hg1201. Therefore, the surface-doped IP of F0223 is expected to reside in a cleaner environment, as the metallic OP layers provide enhanced screening of disorder.

Moreover, our surface-doped IP exhibits a larger superconducting gap and more enhanced coherence than that of Hg1201 [Ref. R18], as evidenced by sharp quasiparticle peaks observed over the entire Fermi surface. It is thus natural to suggest that such a clean IP could exhibit an enhanced T_c beyond the original ~100 K in the pristine samples.

Finally, we highlight the unique crystal structure of the present compounds, which distinguishes them from the Bi- and Hg-based families. In Bi-based systems such as Bi2223, the Bi-O layers, which serve as charge reservoir (dopant) layers, are located above and below the apical oxygen sites of the outer CuO_2 planes (OPs). In contrast, in F0223, the apical oxygen/fluorine layer itself acts as the dopant layer. As a result, the OPs in F0223 are more effectively carrier-doped from the dopant layer than those in Bi2223.

Furthermore, the carrier concentration of each CuO_2 plane increases as its distance from the dopant layer decreases. Therefore, the OPs are generally more heavily doped than the inner planes (IPs). In F0223, this doping imbalance between the OPs and IPs is further enhanced because the OPs are directly bonded to the apical oxygen/fluorine dopant layer. This unique structural configuration naturally explains why F0223 exhibits a larger potential (or doping level) difference between the OP and IP than Bi2223.

That said, we expect that the physics presented in our manuscript is general to all multilayer cuprates, including Bi- and Hg-based compounds. In general, when the OPs—regardless of the compound—are more heavily doped and thus more metallic, their higher carrier mobility results in stronger screening of disorder in the inner planes (IPs). However, in most other cuprates, it is difficult to selectively dope only the outer layers in large amounts without noticeably changing the doping level of the inner layers. In this sense, while the mechanism we discuss is universal to multilayer cuprates, F0223 provides an exceptionally clear manifestation of intrinsic cuprate physics owing to its unique crystal structure.

We added these discussions to the revised version of our manuscript and Supplementary information.

Comment 3.4

Concerning the modelling of the 5 layer compound, where the authors explore different on-site energies for the distinct layer types to model the band structure, so as to find limiting values of the interlayer hopping V. In the supplementary information the authors say they do not consider cases where V1/=V2/=V3/=V4 for simplicity. I understand that an exhaustive modelling of all different combinations would be a lot, but a discussion of potential consequences of differing interlayer hoppings is warranted, given that the splitting between IP1 and IP0 bands is not observed, and the on-site energies between IP0, IP1 and OP are actually quite different.

[1] Kondo, T., et al., (2004). Journal of Electron Spectroscopy and Related Phenomena, 137–140(SPEC. ISS.), 663–668. https://doi.org/10.1016/j.elspec.2004.02.104
[2] Kurokawa, K. et al., (2023), 14(1), 4064. https://doi.org/10.1038/s41467-023-39457-7
[3] Horio, M., et al. (2025). Physical Review Letters, 135(4), 46501. https://doi.org/10.1103/p4c3-t34b
[4] Luo, X., (2023). Nature Physics, 19(12), 1841–1847. https://doi.org/10.1038/s41567-023-02206-0
[5] Smit, S. et al., arXiv:2506.01448 (2025)
[6] Chen, H. et al., arXiv:2508.09881 (2025)

We thank the reviewer for the constructive comment. The interlayer hopping V_l depends primarily on the distance between the CuO_2 planes. Notably, the interplanar distances in the five-layer compound Cu1245 are all nearly identical to ~3.2 Å [Ref. R19]. Therefore, our assumption that $V_1 = V_2 = V_3 = V_4$ is reasonable.

We added this discussion to the revised version of our Supplementary Information.

In our model calculation, both the interlayer hopping V_l and the on-site energy e_l contribute to the band splitting, whereas only the former leads to interlayer wavefunction mixing. The band splitting between IP1 and IP0 observed by ARPES should therefore result from a combination of finite V_l and e_l. To estimate the upper bound of V_l, we reproduced the observed IP1−IP0 splitting using only a finite V_l while setting e_l = 0. This calculation yields a wavefunction localization within the OPs exceeding 95%. We believe that this argument is straightforward and provides sufficient justification for the simplification adopted in our model.

Reference for Reply letter:

R1. Iyo, A., Tanaka, Y., Tokumoto, M. & Ihara, H. High-pressure synthesis and properties of $Ba_2Ca_{n-1}Cu_nO2_n(O,F)_2$ (n=2–5) superconductors. *Physica C: Superconductivity* **366**, 43–50 (2001).

R2. Seah, M. P. & Dench, W. A. Quantitative electron spectroscopy of surfaces: A standard data base for electron inelastic mean free paths in solids. *Surface and Interface Analysis* **1**, 2–11 (1979).
R3. Palczewski, A. D. *et al.* Controlling the carrier concentration of the high-temperature superconductor $Bi_2Sr_2CaCu_2O_{8+\delta}$ in angle-resolved photoemission spectroscopy experiments. *Phys. Rev. B* **81**.104521 (2010).
R4. Ding, H. *et al.* Coherent Quasiparticle Weight and Its Connection to High-T_c Superconductivity from Angle-Resolved Photoemission. *Phys. Rev. Lett.* **87**, 227001 (2001).
R5. Anzai, H. *et al.* Relation between the nodal and antinodal gap and critical temperature in superconducting Bi2212. *Nat. Commun.* **4**, 1815 (2013).
R6. Ideta, S. *et al.* Energy scale directly related to superconductivity in high-T_c cuprates: Universality from the temperature-dependent angle-resolved photoemission of $Bi_2Sr_2Ca_2Cu_3O_{10+\delta}$. *Phys. Rev. B* **85**, 104515 (2012).
R7. Chatterjee, U. *et al.* Observation of a *d*-wave nodal liquid in highly underdoped $Bi_2Sr_2CaCu_2O_{8+\delta}$. *Nature Phys* **6**, 99–103 (2010).
R8.Luo, X. *et al.* Electronic origin of high superconducting critical temperature in trilayer cuprates. *Nat. Phys.* **19**, 1841–1847 (2023).
R9. Horio, M. et al. Enhanced Superconducting Gap in the Outer CuO_2 Plane of the Trilayer Cuprate (Hg, Re)$Ba_2Ca_2Cu_3O_{8+\delta}$. *Phys. Rev. Lett.* **135**, 046501 (2025).
R10. Kondo, T. *et al.* Point nodes persisting far beyond T_c in Bi2212. *Nat. Commun.* **6**, 7699 (2015).
R11. Kurokawa, K. *et al.* Unveiling phase diagram of the lightly doped high-T_c cuprate superconductors with disorder removed. *Nat. Commun.* **14**, 4064 (2023).
R12. Zhong, Y.-G. *et al.* Continuous doping of a cuprate surface: Insights from *in situ* angle-resolved photoemission. *Phys. Rev. B* **98**, 140507 (2018).
R9. Horio, M. et al. Enhanced Superconducting Gap in the Outer CuO_2 Plane of the Trilayer Cuprate (Hg, Re)$Ba_2Ca_2Cu_3O_{8+\delta}$. *Phys. Rev. Lett.* **135**, 046501 (2025).
R10. Kondo, T. *et al.* Point nodes persisting far beyond T_c in Bi2212. *Nat. Commun.* **6**, 7699 (2015).
R11. Kurokawa, K. *et al.* Unveiling phase diagram of the lightly doped high-T_c cuprate superconductors with disorder removed. *Nat. Commun.* **14**, 4064 (2023).
R12. Zhong, Y.-G. *et al.* Continuous doping of a cuprate surface: Insights from *in situ* angle-resolved photoemission. *Phys. Rev. B* **98**, 140507 (2018).
R13. Kivelson, S. A. Making high T_c higher: a theoretical proposal. *Physica B: Condensed Matter* **318**, 61–67 (2002); Berg, E., Orgad, D. & Kivelson, S. A. Route to high-temperature superconductivity in composite systems. *Phys. Rev.* B **78**, 094509 (2008).
R14. Kunisada, S. *et al.* Observation of Bogoliubov Band Hybridization in the Optimally Doped Trilayer $Bi_2Sr_2Ca_2Cu_3O_{10+\delta}$. *Phys. Rev. Lett.* **119**, 217001 (2017).
R15. Ideta, S. *et al.* Hybridization of Bogoliubov Quasiparticles between Adjacent CuO_2 Layers in the Triple-Layer Cuprate $Bi_2Sr_2Ca_2Cu_3O_{10+\delta}$ Studied by Angle-Resolved Photoemission Spectroscopy. *Phys. Rev. Lett.* **127**, 217004 (2021).
R16. Mukuda, H. *et al.* High-T_c Superconductivity and Antiferromagnetism in Multilayered Copper Oxides –A New Paradigm of Superconducting Mechanism–. *J. Phys. Soc. Jpn.* **81**, 011008 (2012).
R17. Shimizu, S. *et al.* Antiferromagnetism, superconductivity, and pseudogap in three-

layered high-T_c cuprates $Ba_2Ca_2Cu_3O_6(F, O)_2$ probed by Cu-NMR. *Phys. Rev. B* **83**, 214514 (2011).
R18. Vishik, I. M. et al. Angle-resolved photoemission spectroscopy study of $HgBa_2CuO_{4+\delta}$. *Phys. Rev. B* **89**, 195141 (2014).

R19.Hirai, M. *et al.* Unconventional Variation of T_c in the Multilayered Cuprate Superconductor $(Cu,C)Ba_2Ca_4Cu_5O_y$. *J. Phys. Soc. Jpn.* **76**, 054701 (2007).

We thank the Reviewers for carefully considering our responses and suggesting constructive comments. Below, we provide our point-by-point responses to all the Reviewers' comments.

Reviewer #1 (Remarks to the Author):

Summary of Reviewer #1

In the revised manuscript, the authors have addressed all of my previous comments and suggestions. The work presents a valuable and rather unique platform for studying the intrinsic high-T_c mechanisms in cuprates, and demonstrates an effective in situ approach to tuning the doping level, which may be broadly applicable to other cuprates and complex oxides. I therefore recommend the manuscript for publication in Nature Communications in its current form.

We appreciate the Reviewer for accepting our manuscript.

Reviewer #2 (Remarks to the Author):

Summary of Reviewer #2

I have carefully examined all the reviewers' reports and the authors' response. This work is potentially important and the authors have spent efforts to address the issues raised by the reviewers.

We appreciate the Reviewer carefully examining our responses and discussions.

Comment 2.1

The main concern shared by all reviewers is the claim of substantial different doping between the OP and IP layers. This concern is directly triggered by the the absence of band splitting. In the authors' response, two possibilities are discussed. First, the bottom OP may simply be too deep, whereas the probing depth of 55 eV photons is extremely short. Therefore, the bottom OP is not probed in the experiment. Second, both the top and bottom OPs are doped whereas the IP remains unchanged. The first explanation is indeed possible, but it remains a reasonable speculation if not demonstrated by the experiment. The second explanation is counterintuitive. It is really hard to imagine that both OP layers are doped by the surface oxygen, but the IP layer in the middle remains unchanged. Such a counterintuitive explanation really needs experimental evidence.

We thank the Reviewer for the assessment of the two proposed possibilities for the absence of OP band splitting.

Regarding direct experimental verification, we note that identifying the oxygen deposition profile with layer selectivity—particularly for the second CuO_2 plane beneath the surface—is extremely challenging and beyond the scope of the present experiment. Accordingly, in the

revised manuscript, we have clarified that these two scenarios should be regarded as speculative possibilities, rather than experimentally established facts.

Importantly, however, we emphasize that identifying the exact microscopic origin of the absence of OP band splitting is not essential to the central conclusion of this work. Regardless of the detailed status of the bottom OP layer—whether it is not probed due to the limited photoelectron escape depth or is doped in a manner similar to the top OP layer—our primary conclusion remains unchanged. Specifically, our experimental data demonstrate that the observed OP becomes strongly overdoped upon surface doping, whereas the electronic structure associated with the IP shows no clear evidence of a substantial change in doping level within the experimental resolution. This contrast coincides with the emergence of a sharp IP coherence peak and an extended flat band near (π, 0), supporting our conclusion that enhanced IP coherence arises from metallic screening by the OP layers.

Comment 2.2

The authors have carried out additional data analysis to demonstrate that "the peak of the surface-doped OP is only about 45% broader than that of the pristine OP, and the linewidth remains within the range reported for cuprates in the literature. Therefore, it is unlikely that our surface was severely damaged enough to eliminate superconductivity." I appreciate the authors' efforts, and I agree that the data analysis on the OP band makes sense by itself. However, I also need to point out that the linewidth of IP changes much greater than 45% in the same data analysis (Fig. R1 d,e). It is hard to imagine that the surface degradation of IP is stronger than OP on the top.

We thank the Reviewer for pointing out this issue. The apparent increase in the IP linewidth—exceeding 45%, as noted by the Reviewer—can be attributed to the increased energy separation between the IP and OP bands induced by surface doping, rather than the degradation of IP (see Figs. R1a and R1b, and Fig. 4d, which show ARPES intensity maps and curves measured along the nodal direction).

The momentum point highlighted by the Reviewer corresponds to the Fermi momentum of the OP band ($k_{\mathrm{F}}^{\mathrm{OP}}$), where the OP spectral peak is located near the Fermi level, while the IP peak appears at higher binding energy. In the pristine surface, the energy separation between the OP and IP peaks at this momentum is small, whereas surface doping significantly increases this separation. As a result, the IP peak shifts to higher binding energy at $k_{\mathrm{F}}^{\mathrm{OP}}$. In cuprates, spectral linewidths generally become broader with increasing binding energy. Therefore, the IP peak at $k_{\mathrm{F}}^{\mathrm{OP}}$ shows an apparent broadening without implying enhanced disorder.

We hope that the above argument adequately addresses the Reviewer's concern. Nevertheless, it is still important to compare the spectral width at each k_{F} for the IP bands ($k_{\mathrm{F}}^{\mathrm{IP}}$) before and after surface doping. As demonstrated in Fig. R1 and discussed below, we find that the linewidths of the spectral peaks for the pristine and surface-doped IP show no significant difference, indicating that no significant additional disorder is introduced into the IP by surface doping.

In Fig. R1c, we compare near-nodal EDCs of the pristine and surface-doped F0223. Each curve is extracted at k_F^{IP} and overlaid with spectra taken at a similar angle from the node ϕ (see inset of Fig. 4m). Notably, the linewidths of the IP coherence peaks for the pristine and surface-doped samples show no significant difference.

One may notice that the original nodal IP spectrum ($\phi \approx 0°$) of the surface-doped sample shown in Figs. 4k and 4l of the manuscript appear relatively broad (magenta curve at $\phi \approx 0°$ in Fig. R1c). We emphasize that this does not indicate degradation of the IP upon surface doping, but rather arises from experimental conditions. Since ARPES measurements are highly sensitive to the relative alignment between the cleaved sample surface and the incident beam, subtle sample misalignment can lead to apparent spectral broadening. To avoid confusion, we have replaced the nodal EDC of surface-doped IP shown in the revised manuscript (ϕ~0° curves in Figs. 4k, l) with the higher-quality data.

We added all the above discussion to the revised version of our Supplementary Information.

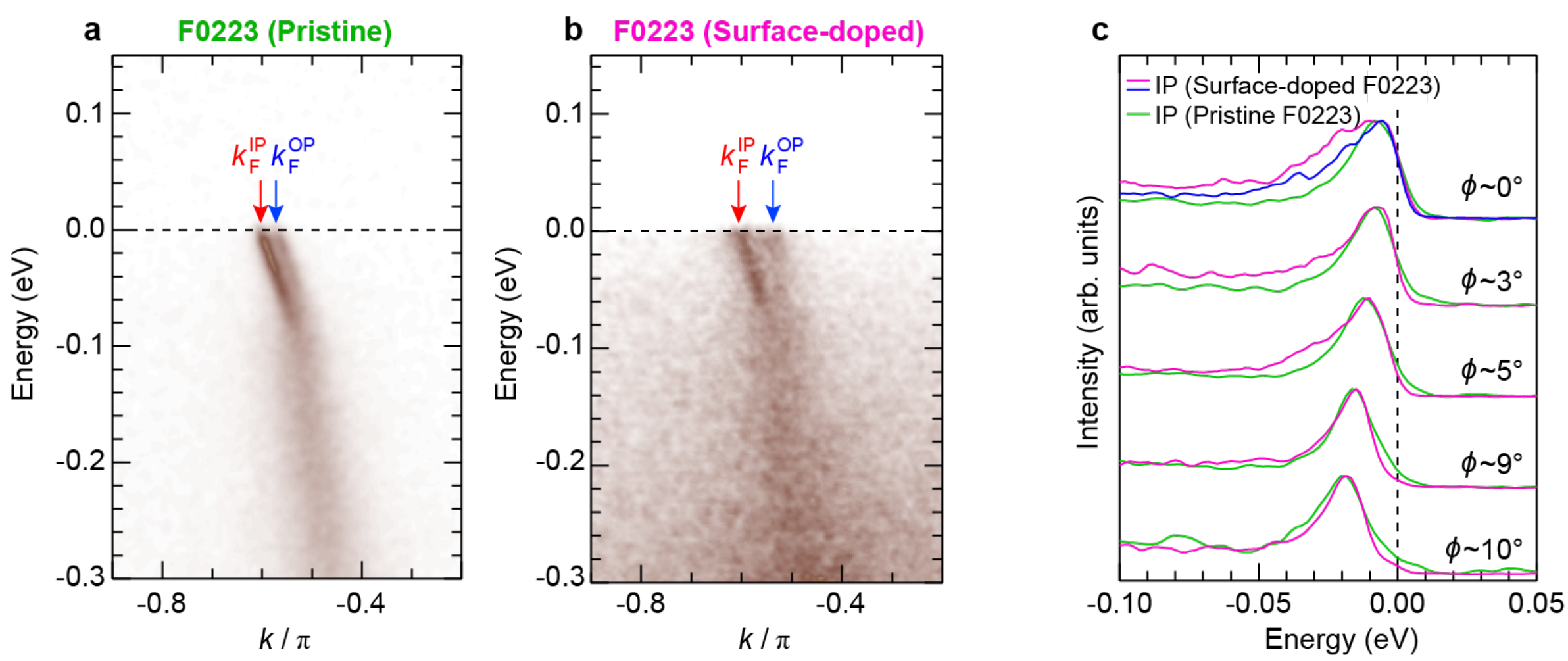

Fig. R1 a, Energy-momentum dispersion of pristine F0223, along the nodal direction. Red and blue arrow indicates the Fermi momentum (k_F) point of IP and OP (k_F^{IP} and k_F^{OP}, respectively), respectively. **b**, Similar measurement with **a**, but for the surface-doped F0223. **c**, Comparison of near-nodal EDCs of pristine- (green curves) and surface-doped (magenta and blue curves) F0223. Blue curve at ϕ~0° is the higher-quality data from the same sample obtained after re-optimizing the sample position. Each curve was extracted from k_F points of IP. ϕ is an angle from the node (see inset of Fig. 4m).

Comment 2.3

As I mentioned in the previous report that the investigation of multilayer cuprates is important. In principle, I would be happy to see that the current study can be published in Nature Communications. Nevertheless, I would still suggest the authors to make the discussions self-consistent, and demonstrate the speculated issues, if at all possible.

We appreciate the Reviewer's positive assessment and agree that multilayer cuprates provide

a unique and important platform for investigating the nature of high-T_c superconductivity. In response to the Reviewer's suggestion, we have carefully revised the manuscript to ensure that the discussions are self-consistent and that all speculative points are clearly identified and supported by additional data analysis. We hope that these revisions address the Reviewer's concerns.

Reviewer #3 (Remarks to the Author):

Summary of Reviewer #3

I appreciate the authors extensive response to the reports and some of the revisions made to the manuscript, many of which have strengthened paper. However I remain unconvinced about key aspects of the data interpretation, that are crucial to the drawn conclusions. In particular regarding the doping of the IP FS of the trilayer sample - I reiterate here that this is important because small changes in doping can very plausibly sharpen up quasiparticles.

We thank the Reviewer for carefully examining our responses and for the constructive summary of the remaining concerns. We fully acknowledge that the issue of IP doping is indeed important for the interpretation of our data and for the central conclusion regarding enhanced coherence via metallic screening by the OP layers. Below, we therefore address this point in detail by clarifying the experimental observations and their implications.

Comment 3.1

The authors claim less than 1% change of doping on the IP FS after air exposure, based on two things - the near nodal gapsize and Fermi momenta. In their response the authors say that the bands near (pi,0) are insensitive to doping variations at low doping (due to the deep lying van Hove points) without providing evidence for this. Given that correlations can change drastically with doping, especially in this regime of low doping, a rigid band-shift model is not at all obvious to be applicable here. Furthermore the authors claim that the pristine IP FS is large and (pi,pi) centered without being able to follow the dispersions outside the AFBZ. As such it is unclear to me how the tight binding model for this data is determined.

We appreciate the Reviewer's insightful comment. We agree that extracting the precise energy position of the band bottom (or van Hove point) from our experimental data is challenging, due to the strong pseudogap effect and the presence of an extended flat band near (π, 0). Accordingly, we have removed our initial claim that the doping level difference between the pristine and surface-doped IP is less than 1% in the revised manuscript.

Nevertheless, our main conclusion—that superconducting coherence is enhanced by metallic screening from the outer layers—remains robust, as discussed below.

First, we note that the pristine IP exhibits a large Fermi surface centered at (π, π), rather than a small Fermi pocket centered at (π/2, π/2), for the following reason. A band forming a small Fermi pocket disperses deeply down to binding energies of ~1 eV at (π, 0), as demonstrated

by Kunisada et al. [Ref. R1]. In contrast, the present IP-band data show spectral features confined to an energy scale that is more than one order of magnitude smaller, on the order of 0.1 eV, at (π, 0). This behavior is consistent with the pseudogap state in cuprates, which is associated with a large Fermi surface.

In the overdoped regime of cuprates (*e.g.*, overdoped Bi2201 reported by Kondo et al. [Ref. R2]), the large Fermi surface centered at (π, π) exhibits a significant shift upon carrier doping around the antinodal region (π, 0), while the shift around the nodal region is much smaller. In contrast, in the heavily underdoped regime, the Fermi surface shows an almost parallel shift upon carrier doping over the entire Brillouin zone (*e.g.*, underdoped Bi2201 reported by Hashimoto et al. [Ref. R3]). Furthermore, it has been demonstrated that doping evolution in this regime is well described by a rigid-band shift. These observations indicate that the heavily underdoped IP in the present sample can also be understood, to first approximation, within a rigid-band picture. Accordingly, the Fermi surface determined around the nodal Fermi-arc region provides a reasonable estimate of the doping variation.

That said, we fully acknowledge the Reviewer's concern. The strong pseudogap effect and the presence of a flat band near (π, 0) make it difficult to determine k_F precisely in the antinodal region, preventing a highly accurate quantification of the doping difference. Consequently, we have removed our initial claim that the doping level difference between the pristine and surface-doped IP is less than 1% in the revised manuscript.

Our main conclusion is that electronic coherence is well established, even in the heavily underdoped regime, in momentum regions beyond the antiferromagnetic zone boundary (AFZB), owing to metallic screening by the outer layers. This finding runs counter to the general notion derived from many previous studies of heavily underdoped cuprates.

This point can be clarified by comparing our ARPES data with those of the more familiar trilayer cuprate Bi2223, recently reported by Luo et al. [Ref. R4]. Figure R2a shows the Fermi surface of overdoped Bi2223 with tight-binding fits (colored solid curves). By overlaying the near-nodal k_F points of IP for the pristine and surface-doped F0223 (also shown in the inset of Fig. 4m), it becomes evident that the doping level of both the pristine and surface-doped IP in F0223 is substantially lower than that of the IP in overdoped Bi2223.

Importantly, the IP of Bi2223 has a much higher doping level; yet its coherence peak rapidly disappears upon crossing the AFZB (Fig. R2b). This behavior stands in stark contrast to the sharp coherence peak observed in the IP of surface-doped F0223 (Fig. 5m). This comparison between F0223 and Bi2223 strongly supports our conclusion that the enhanced coherence of the underdoped IP in F0223 is driven by metallic screening from the OP layers. Unlike Bi2223—where the IP coherence peak is suppressed across the AFZB, similar to the IP in pristine F0223 and other underdoped cuprates [Refs. R5 and R6]—the surface-doped F0223 retains a robust coherence peak beyond the AFZB and even at (π, 0) (Fig. 5m).

We therefore believe that this line of discussion provides more robust support for the main message of our paper than insisting on a quantitative claim such as "the doping difference between pristine- and surface-doped IP is less than 1%", and further clarifies the novelty of our

results. Accordingly, we have added this argument and Fig. R2 to the revised version of our manuscript.

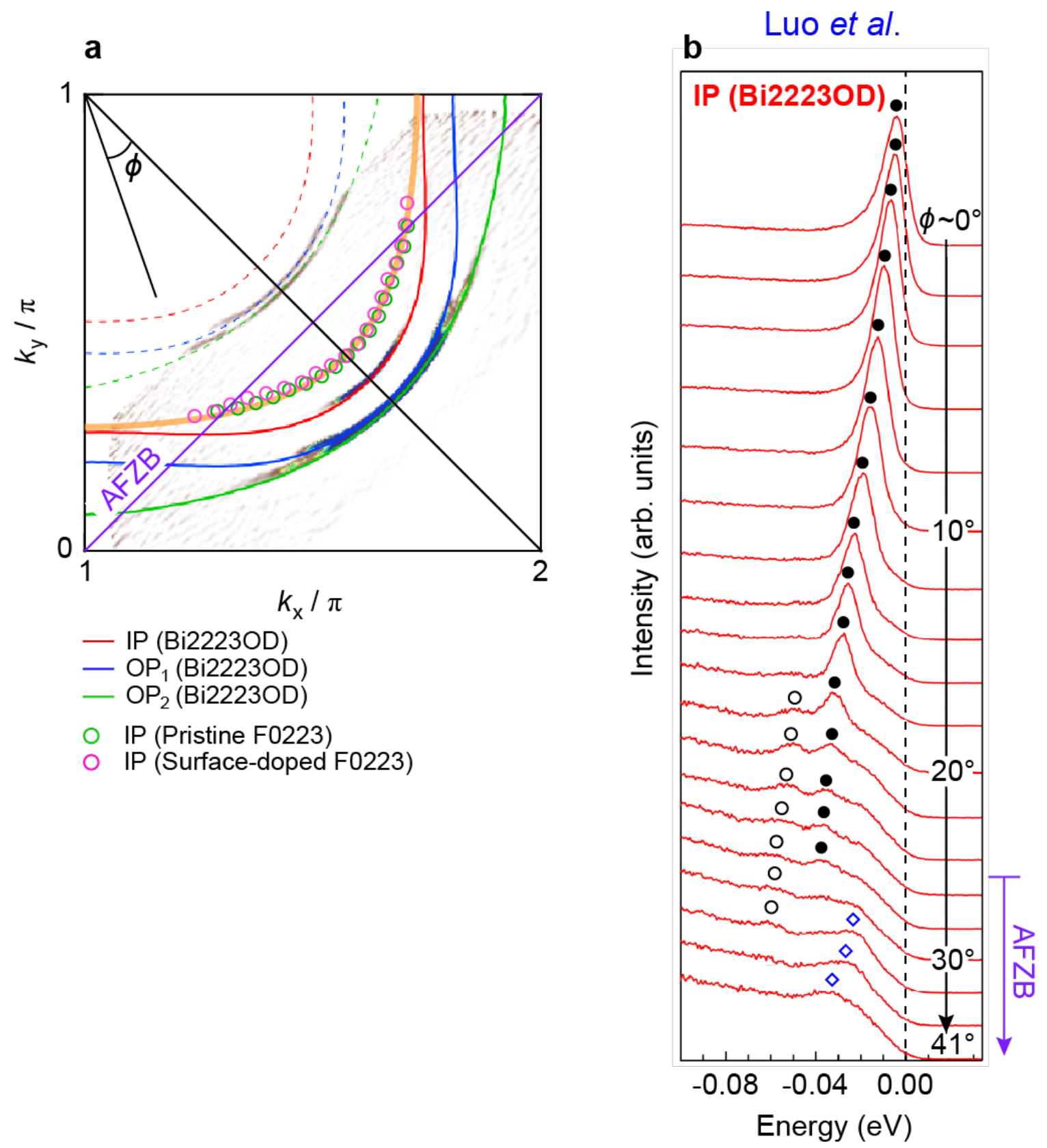

Fig. R2 a, The Fermi surface of overdoped Bi2223 (Bi2223OD) [Ref. R4], overlaid with the IP k_F points of pristine- and surface-doped F0223 (same data shown in the inset of Fig. 4m). The colored solid curves are the tight-binding fits of each band. Dashed curves represent a shadow band originating from the superstructure of Bi2223. **b**, EDCs of IP in Bi2223OD, from nodal (ϕ~0°) to antinodal direction (ϕ~45°). Black circles indicate the peak position of the IP coherence peak. Empty circles indicate the band hybridization of IP with OP. Blue diamonds are the peak positions of OP_1 (adapted from Luo *et al.* [Ref. R4]).

Comment 3.2

Regarding the gapsize the authors cite two papers ("R5" and "R12") and say that "the superconducting gap near the node varies significantly with doping in the underdoped region". This feels exaggerated and not necessarily supported by the data shown in those papers. Additional high resolution laser-ARPES studies (for example Ref 10, [Vishik, I. M. *et al.* PNAS 109, 18332–18337 (2012)]) make the point that the near-nodal gapsize (for samples with a true node in the gap function) is very consistent across doping.

We thank the Reviewer for raising this important issue. We acknowledge that the doping dependence of the superconducting gap near the node remains a subject of debate in the field, particularly in the underdoped regime, where the Fermi arc becomes extremely short.

As the Reviewer pointed out, Vishik et al. [Ref. R7] reported a nearly constant near-nodal gap size across a wide doping range, from underdoped to slightly overdoped samples. On the other hand, in the same year (2012), Anzai et al. [Ref. R8] reported that the superconducting gap magnitude changes significantly with carrier doping, roughly following the T_c value. More recently, Zhong et al. (2018) [Ref. R9] reported results consistent with those of Anzai et al. Taken together, these studies indicate that the evolution of the superconducting gap near the node in cuprates remains a controversial issue.

Given this lack of consensus, we agree that using the "identical gap size" alone is insufficient for making a robust claim regarding the doping difference. To avoid such ambiguity, we have removed our initial claim that the doping level difference between the pristine and surface-doped IP is less than 1%. Instead, we have adopted a more straightforward approach by comparing our results with those of Bi2223, as discussed in our response to **Comment 3.1**. This comparison provides stronger evidence for our conclusion without relying on the contested doping-dependent behavior of the gap size, particularly in the underdoped regime of cuprates.

We added these discussions to the revised version of our manuscript.

References for Reply letter:

R1. Kunisada, S. *et al.* Observation of small Fermi pockets protected by clean CuO2 sheets of a high-T_c superconductor. *Science* **838**, 833–838 (2020).

R2. Kondo, T. *et al.* Hole-concentration dependence of band structure in $(Bi, Pb)_2(Sr, La)_2CuO_{6+\delta}$ determined by the angle-resolved photoemission spectroscopy. *J. Electron Spectrosc. Relat. Phenom.* **137**, 663–668 (2004).

R3. Hashimoto, M. *et al.* Doping evolution of the electronic structure in the single-layer cuprate $Bi_2Sr_{2-x}La_xCuO_{6+\delta}$: Comparison with other single-layer cuprates. *Phys. Rev. B* **77**, 094516 (2008).

R4. Luo, X. *et al*. Electronic origin of high superconducting critical temperature in trilayer cuprates. *Nat. Phys*. **19**, 1841–1847 (2023).

R5. Kohsaka, Y. *et al.* How Cooper pairs vanish approaching the Mott insulator in $Bi_2Sr_2CaCu_2O_{8+\delta}$. *Nature* **454**, 1072–1078 (2008).

R6. Kondo, T. et al. Evidence for Two Energy Scales in the Superconducting State of Optimally Doped $(Bi, Pb)_2(Sr, La)_2CuO_{6+\delta}$. Phys. Rev. Lett. **98**, 267004 (2007).

R7. Vishik, I. *et al*. Phase competition in trisected superconducting dome. *Proc. Natl. Acad. Sci. U.S.A*. **109**, 18332–18337 (2012).

R8. Anzai, H. *et al.* Relation between the nodal and antinodal gap and critical temperature in superconducting Bi2212. *Nat. Commun.* **4**, 1815 (2013).

R9. Zhong, Y.-G. *et al.* Continuous doping of a cuprate surface: Insights from *in situ* angle-resolved photoemission. *Phys. Rev. B* **98**, 140507 (2018).

We thank the Reviewer for carefully considering our responses and suggesting constructive comments. Below, we provide our point-by-point responses to all the Referee's comments.

Reviewer #1 (Remarks to the Author):

Summary of Reviewer #1

I have re-read the revised manuscript, the review reports, and the authors' rebuttal. Overall, I find that the authors have made substantive revisions addressing the major concerns raised in previous rounds. My assessment is as follows. In my opinion, the experimental findings remain important and merit publication in Nature Communications.

We appreciate the Reviewer's positive assessment and support for publication of our manuscript in Nature Communications.

Comment 1.1

1. A central concern raised by all reviewers was the lack of observable band splitting after surface doping. In the revised version, the authors appropriately acknowledge that their explanation is speculative, and they have also removed the overly strong claim that the IP doping change is below 1%. I consider this an improvement. Although the microscopic mechanism of the surface doping effect is still not fully clarified, the key experimental observations that the IP remains highly underdoped while the OP becomes substantially more doped, appear sufficiently supported and continue to underpin the main conclusions of the manuscript.

We thank the Reviewer for the positive assessment of our revised manuscript.

Comment 1.2

2. The comparison with Bi2223 (Fig. R2) is useful in supporting the claim that the IP is highly underdoped. To further strengthen the interpretation, I suggest that the authors also compare the OP Fermi surface after surface doping with the OP Fermi surface of Bi2223 in the same plot (Fig. R2). Such a comparison could provide additional support for the scenario in F0223 that a highly overdoped metallic OP layer screens the highly underdoped IP layer and helps enhance superconducting coherence.

We appreciate the Reviewer's constructive suggestion. Following this, we have added the OP Fermi surfaces (k_F points) of F0223 to the Fermi surface map of Bi2223 [Ref. R1] in the revised supplementary figure (shown as Fig. R1 below). It is now clear that the OP in F0223 is heavily overdoped.

Here, we note that the OP bands in Bi2223 exhibit bilayer splitting, and therefore, the doping level is estimated from the average Fermi surface volume. According to Ref. R1, the doping levels of the two OP bands (OP_1 and OP_2) are estimated to be $p \approx 0.22$ and 0.37, respectively, yielding an average of $p \approx 0.30$, which is comparable to that of the surface-doped F0223 ($p \approx 0.30$). Such a high doping level lies well beyond the edge of the universal T_c dome commonly

observed in cuprates. Nevertheless, the OP in Bi2223 remains superconducting even in this heavily overdoped regime. Furthermore, unlike in surface-doped F0223, the IP of Bi2223 does not show enhanced coherence beyond the AFZB. This contrast may originate from the significant interlayer hybridization between IP and OP in Bi2223 [Refs. R1, R2], which can transmit disorder from the OP to the IP. In contrast, the interlayer coupling in apical-F multilayer cuprates is much weaker, isolating the IP from disorder effects and thereby promoting enhanced coherence.

We have added this discussion, along with the revised Fig. R1, to the Supplementary Information.

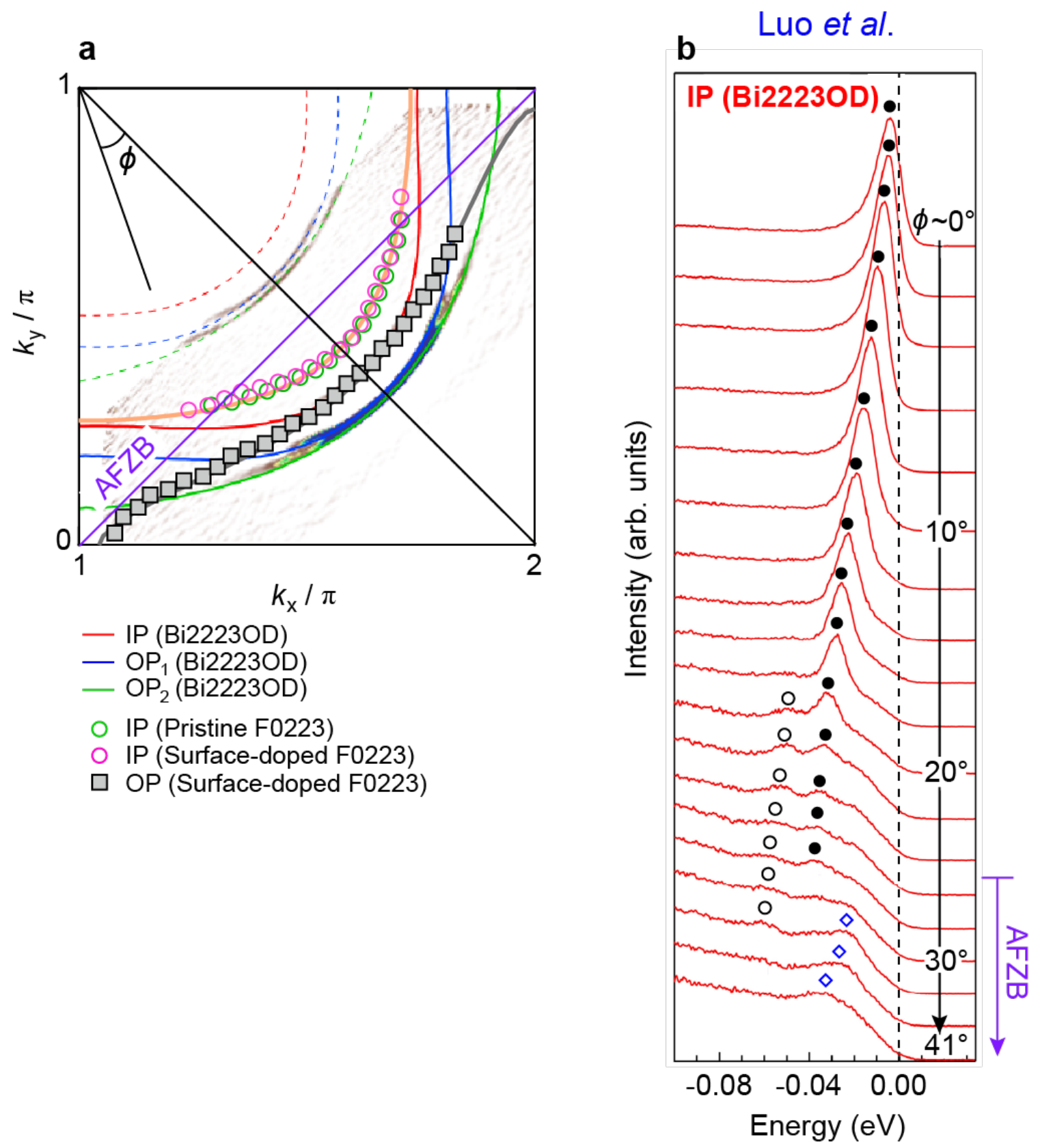

Fig. R1 a, The Fermi surface of overdoped Bi2223 (Bi2223OD) [Ref. R1], overlaid with the k_F points of pristine- and surface-doped F0223 (same data shown in the inset of Fig. 4m). The colored solid curves are the tight-binding fits of each band. Dashed curves represent a shadow band originating from the superstructure of Bi2223. **b**, EDCs of IP in Bi2223OD, from nodal (ϕ~0°) to antinodal direction (ϕ~45°). Black circles indicate the peak position of the IP coherence peak. Empty circles indicate the band hybridization of IP with OP. Blue diamonds are the peak positions of OP_1 (adapted from Luo *et al.* [Ref. R1]).

Reviewer #2 (Remarks to the Author):

Summary of Reviewer #2

> The authors have tried to address the remaining issues without providing additional experimental evidence. I disagree with the authors' point that the absence of OP band splitting is not essential to the central conclusion of this work. The absence of OP band splitting is directly linked to the central question that whether the doping level of each layer is correctly estimated. I have to point out that the correct estimation of the doping level is the basis for the key conclusion of this paper.

We appreciate the Reviewer's important comment. We agree that the estimation of the layer-dependent doping level is central to our conclusions. In this regard, we emphasize that our determination of the doping level does not rely on resolving multiple OP bands, but is instead based on the directly measured Fermi surface, which provides a robust and widely established measure of carrier concentration in cuprates.

Our data clearly show that the OP is strongly overdoped, well beyond the T_c dome, whereas the IP does not show a substantial change in doping level within the experimental resolution. This contrast is directly supported by the observed Fermi surface and is independent of whether additional OP bands from deeper layers are resolved.

Furthermore, the comparison with Bi2223 introduced in the revised manuscript reinforces this conclusion. Therefore, while we acknowledge that resolving additional OP bands would provide further insight, the central conclusion of our work does not depend on this point.

Comment 2.1

> In principle, systematic photon energy dependent measurements would clearly demonstrate the authors' first speculation. If the band splitting is observed, then all the experimental observations would become more reasonable. Nevertheless, if the authors decide not to provide additional experimental evidence, then it is important to state the speculations and clarify the possible uncertainties related to the key conclusion of this paper.

We thank the Reviewer for this important comment. We agree that systematic photon-energy-dependent measurements would be valuable to further clarify the layer-resolved electronic structure. At the same time, we note that in many surface-doping ARPES studies, including those using alkali-metal deposition, distinct band contributions from deeper layers have not been observed, to the best of our knowledge, despite clear changes in carrier concentration.

Importantly, however, our central conclusion does not rely on resolving these additional bands but is based on the directly measured Fermi surface topology, which robustly demonstrates that the OP is strongly overdoped, while the IP does not show a clear change in doping level within the experimental resolution. Therefore, while our conclusion does not depend on resolving these additional bands, we consider that the absence of OP band splitting should be regarded as a source of uncertainty. While photon-energy-dependent measurements may provide further insight, our main conclusions remain valid within the current experimental

framework.

Here, we refer, in more detail, to related ARPES studies on surface-doped samples, which show that doping changes the band but does not introduce an additional band from the deeper layers.

Over the years, numerous ARPES studies have utilized surface treatments (such as alkali metal or oxygen deposition) to tune carrier concentrations in various classes of layered materials, including cuprates [Refs. R3, R4], iron-based superconductors [Ref. R5], semiconductors [Ref. R6], and transition-metal dichalcogenides (TMDs) [Ref. R7]. However, distinct band splitting originating from the differently doped deeper layers has not, to the best of our knowledge, been observed in the vast majority of these studies.

In particular, in a systematic surface potassium-deposition study on the $YBa_2Cu_3O_{6+x}$ (YBCO) system [Ref. R3], band splitting from the deeper CuO chain is not observed with 110 eV photons, although a clear change in the FS volume is detected. We note that the distance of the deeper CuO chain from the surface in YBCO (~11.7 Å) is comparable to our F0223 case. Additionally, in a study utilizing surface oxygen doping on Bi2212, band splitting from the bottom CuO_2 layer (or deeper unit cell) is not observed with 21 eV photons [Ref. R4]. Lastly, in a potassium-deposition study on the surface of $HfTe_2$, a layered TMD, it has been suggested that the surface-deposited potassium atoms diffuse into the bulk (intercalation) [Ref. R7]. A similar diffusion process may also occur in the oxygen surface-doped case, which could account for the absence of band splitting in *in-situ* surface-doping experiments using potassium and oxygen.

The exact mechanism behind this ubiquitous absence—whether governed by short probing depth, complex matrix element effects, surface disorder broadening, or rapid dopant diffusion—remains an open question in the field of surface-doping ARPES. While we agree with the Reviewer that systematic photon energy-dependent measurements would be valuable to investigate this specific surface physics problem, fully resolving this methodological open question is not the focus of our study. Instead, our work focuses on the many-body physics and layer-dependent electronic structures of multilayer cuprates.

We have added the summary above of previous surface-doping studies, including the absence of signals from deeper layers. We expect that this clarification will help readers beyond the ARPES community recognize this common issue and motivate future studies to address it.

That said, we agree that it is important to maintain a balanced discussion in the manuscript. While the unique properties of the IP in our material, together with the comparison with Bi2223 introduced in the previous revision, strongly support our conclusion, we acknowledge that some uncertainty remains due to the comparison between different material systems. Therefore, we have added a new paragraph in the main text as follows:

"While this comparison supports our interpretation, F0223 and Bi2223 are distinct material systems, and their IPs may not behave identically with doping. A more definitive test would involve devising a method to tune the IP doping while keeping the OP carrier concentration

fixed within a single material and tracking the resulting evolution of coherence. However, such control is currently challenging with our surface treatment technique and remains a subject for future study."

We also revised the relevant paragraph in the supplementary material as follows:

Before:
"This line of discussion provides robust support for our main conclusion in this article that electronic coherence is well established, even in the heavily underdoped regime, in momentum regions beyond the antiferromagnetic zone boundary (AFZB), owing to metallic screening by the outer layers. This finding runs counter to the general notion derived from many previous studies of heavily underdoped cuprates."

After:
"This line of discussion supports our main conclusion in this article that electronic coherence is well established, even in the heavily underdoped regime, in momentum regions beyond the antiferromagnetic zone boundary (AFZB), owing to metallic screening by the outer layers. However, it is also true that F0223 and Bi2223 are different materials. To fully clarify this point, further studies would be required; for example, by devising a method to vary IP doping while keeping the OP carrier concentration fixed, and examining the corresponding evolution of coherence in the IP."

Here, we note that, in multilayer cuprates, it is well established that the OPs are more strongly doped than the IP, as they are directly adjacent to the charge reservoir layers. Surface doping primarily modifies these outer layers, leading to a significant change in OP doping but a much weaker effect on IP.

In the revised manuscript, we have also modified the Conclusion section to more clearly emphasize the significance of the results obtained from the five-layer cuprates. In particular, the five-layer system exhibits an unprecedented state in which only the inner CuO$_2$ planes are superconducting, while the outer planes remain metallic. This finding is unique among cuprate systems and provides the key physical basis for our study.

Based on this result, we developed a strategy to further explore the underlying physics using the three-layer system. The central concept is interlayer decoupling, achieved through excess doping of the outer layers, which creates a large doping-level (or potential) difference between layers and suppresses wavefunction mixing.

The investigation of the three-layer system was thus motivated by the intrinsic decoupling revealed in the five-layer system and serves as an ideal realization of the same mechanism. In this way, we have reorganized the presentation to make the underlying line of reasoning—from the initial observation, to the physical mechanism, to the experimental strategy, and finally to its realization—more explicit. By clarifying this point, we aim to make the logical structure and the central physical insight of our work more explicit and robust in the revised manuscript.

We believe that these revisions address the remaining uncertainties and improve the overall

balance of the manuscript.

Reviewer #3 (Remarks to the Author):

Summary of Reviewer #3

I appreciate the authors response and think the decision to remove the claim that the doping level difference between the pristine and surface-doped IP is less than 1% is a correct one. I also appreciate the direct comparison to the IP of Bi2223, which makes it clear that the surface-doped F0223 IP is much more coherent outside of the AFZB, despite having a significantly lower doping level.

We thank the Reviewer for the positive assessment of our revised manuscript.

Comment 3.1

At the same time, it is important to recognize that these are two different material systems, potentially with different underlying phenomenologies. Therefore, it is not strictly guaranteed that the two IPs should behave identically as a function of doping. While the comparison provides a useful indication supporting the authors' interpretation, this limitation should be explicitly acknowledged. In particular, it remains possible that dopants contribute in some way to the enhanced coherence of the F0223 IP.

For this reason, I recommend softening the language in the newly added section and incorporating a more balanced discussion reflecting this caveat. The data remain novel and interesting, and I am in favour of publication provided the discussion is adjusted accordingly.

We thank the Reviewer for the positive assessment of our results and for pointing out this important limitation. It is true that F0223 and Bi2223 are different material systems, which limits a completely fair comparison between them. Accordingly, we softened the language in the relevant section and added a new paragraph in the main text as follows:

"While this comparison supports our interpretation, F0223 and Bi2223 are distinct material systems, and their IPs may not behave identically with doping. A more definitive test would involve devising a method to tune the IP doping while keeping the OP carrier concentration fixed within a single material and tracking the resulting evolution of coherence. However, such control is currently challenging with our surface treatment technique and remains a subject for future study."

We also revised the relevant paragraph in the supplementary material as follows:

Before:

"This line of discussion provides robust support for our main conclusion in this article that electronic coherence is well established, even in the heavily underdoped regime, in momentum regions beyond the antiferromagnetic zone boundary (AFZB), owing to metallic screening by the outer layers. This finding runs counter to the general notion derived from many previous

studies of heavily underdoped cuprates."

After:
"This line of discussion supports our main conclusion in this article that electronic coherence is well established, even in the heavily underdoped regime, in momentum regions beyond the antiferromagnetic zone boundary (AFZB), owing to metallic screening by the outer layers. However, it is also true that F0223 and Bi2223 are different materials. To fully clarify this point, further studies would be required; for example, by devising a method to vary IP doping while keeping the OP carrier concentration fixed, and examining the corresponding evolution of coherence in the IP."

While we acknowledge this limitation, the significantly enhanced coherence observed in the CuO_2 layer in such a heavily underdoped regime is highly unusual and has not been reported in the extensively studied standard cuprates. Our results from new material and a rather unique doping control method will promote further research in condensed matter physics.

References for the Reply letter:

R1. Luo, X. *et al*. Electronic origin of high superconducting critical temperature in trilayer cuprates. *Nat. Phys*. **19**, 1841–1847 (2023).

R2. Kunisada, S. *et al*. Observation of Bogoliubov band hybridization in the optimally doped trilayer $Bi_2Sr_2Ca_2Cu_3O_{10+\delta}$. *Phys. Rev. Lett.* **119**, 217001 (2017).

R3. Fournier, D. *et al.* Loss of nodal quasiparticle integrity in underdoped $YBa_2Cu_3O_{6+x}$. *Nat. Phys.* **6**, 11 (2010).

R4. Palczewski, A. D. *et al.* Controlling the carrier concentration of the high-temperature superconductor $Bi_2Sr_2CaCu_2O_{8+\delta}$ in angle-resolved photoemission spectroscopy experiments. *Phys. Rev. B* **81**.104521 (2010).

R5. Kyung, W. S. *et al.* Enhanced superconductivity in surface-electron-doped iron pnictide $Ba(Fe_{1.94}Co_{0.06})_2As_2$. *Nat. Mater.* **15**, 12 (2016).

R6. Chen, L. *et al.* Doping-controlled transition from excitonic insulator to semimetal in Ta_2NiSe_5. *Phys. Rev. B* **102**, 161116 (2020).

R7. Nakata, Y. *et al*. Dimensionality reduction and band quantization induced by potassium intercalation in 1*T*-$HfTe_2$. Phys. Rev. Mater. **3**, 071001 (2019).
